# Poly Caprolactam Supported Hexaethylene Glycolic Imidazolium Ionic Liquid as a Heterogeneous Promoter for Nucleophilic Fluorination

**DOI:** 10.3390/molecules28186747

**Published:** 2023-09-21

**Authors:** Mudumala Veeranarayana Reddy, Keun Heok Park, Dong Wook Kim

**Affiliations:** Department of Chemistry and Chemical Engineering, Inha University, 100 Inha-ro, Nam-gu, Incheon 402-751, Republic of Korea; drvnreddym@gmail.com (M.V.R.); p.22211388@inha.edu (K.H.P.)

**Keywords:** ionin liquid, polymer supported ionic liquid, nucleophilic fluorination, heterogeneous promoter

## Abstract

Hexaethylene glycolic vinyl imidazolium (hexaEGVIM) was supported on *N*-vinyl caprolactam via covalent bonds through simple copolymerization to form poly caprolactam-supported hexaethylene glycol-substituted imidazolium salts (PCLS-hexaEGIM). The resulting heterogeneous PCLS-hexaEGIM promoter was active, selective, and stable for aliphatic nucleophilic substitution reactions using alkali metal salts. The alkali metal salts dramatically enhanced the reactivity of this heterogeneous catalyst with easily isolable higher product yields, reducing the formation of by-products. Therefore, nucleophilic fluorination and other substitution reactions can act as highly efficient catalysts in various sulfonyloxyalkanes and haloalkanes with regard to their corresponding fluorinated products.

## 1. Introduction

A range of materials in the chemical industry of organic molecules containing fluorine atom(s) in agrochemicals and pharmaceuticals have widespread applications, leading to significant changes in their physical, chemical, and biological properties [1,2,3,4,5,6]. Alkali metal fluorides are economical and potent sources of fluoride [7,8], but phase transfer catalysts (PTC) are needed to overcome their insolubility and low nucleophilicity [9,10].

For functional group transformation in organic synthesis, ionic liquids (ILs) have attracted increasing attention recently, and have succeeded to some extent [11,12,13]. In particular, imidazolium-based ILs are a well-known IL series, with potential phase transfer catalytic activity that improves the solubility of alkali metal salts, resulting in easy nucleophilic substitution reactions of fluorination [14,15,16]. On the other hand, for the preparation of fluorinated molecules available, a range of direct C–F bond formation methods have integrated technologies for the molecular incorporation of other heteroatoms [2,3,4].

Organic reactions using catalysis are more efficient and selective, eliminating by-products and significantly reducing pollution [17]. Thereby, catalysis is a vital process in the chemical industry used to synthesize an enormous range of products and fine chemicals. Therefore, it is considered the most preferred and relevant technology to reduce waste from chemical processes and is one of the fundamental pillars of green chemistry [18].

Science and technology have recently shifted towards eco-friendly processes, encouraging reusable catalysts and natural product resources. Thus, researchers have paid attention to well-recognized polymer-supported compounds as supported catalysts that are non-toxic, economical, non-volatile, easily produced via the simple separation of insoluble catalysts from products, and easily compatible with industrial processes. Hence, they have emerged as versatile supporting materials for deploying various catalysts [19,20]. Among them, polymer-supported ionic liquids (PSILs) are an emerging interdisciplinary area that can be applied to tackle controversial scientific issues and have played crucial roles in different fields of science [21,22,23]. Therefore, the synthesis, chemistry, and capabilities of these materials using catalysts and reagents for different synthetic and industrial applications have been widely investigated and reported. These processes involve various nucleophilic substitution reactions and follow the fundamentals of green chemistry, the versatility of PSILs, and the easy isolation of products [24,25].

**Figure 1:**
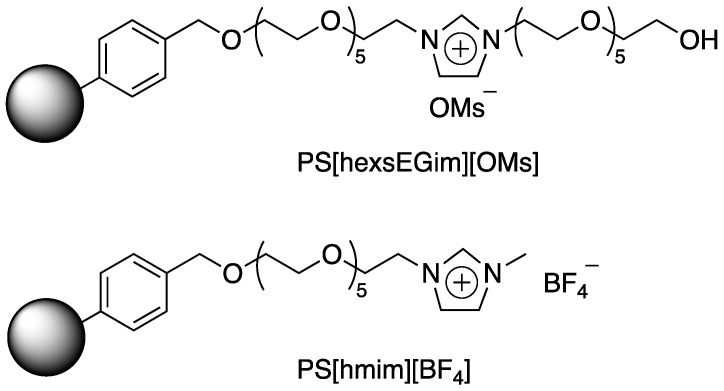
Polymer-supported imidazolium salts.

A previous study reported remarkable nucleophilic fluorination with higher reactivity and selectivity using polystyrene-supported hexaethylene glycol-substituted imidazolium salts (PS[hexaEGim][OMs]) and polymer-supported imidazolium salts (PS[hmim][BF_4_]), which are provided by the reduced basicity of the fluoride ion. In addition, the solubility and reactivity of the anions can be increased by the polar hydroxyl group of [hexaEGim][OMs] (Figure 1). This process’ catalytic activity was limited to homogeneous conditions, but it showed superior PTC activity in various nucleophilic substitution reactions under heterogeneous protocols with alkyl sulfonates and alkyl halides [26,27]. Herein, we introduce a heterogenous catalyst consisting of poly caprolactam-supported hexaethylene glycol-substituted imidazolium salts (PCLS-hexaEGIM). In addition, the efficiency of its catalytic activity in nucleophilic fluorination reactions with various substrates of sulfonate and halo-leaving groups was investigated using alkali metal fluorides (MFs).

## 2. Result and Discussion

**Scheme 1:**
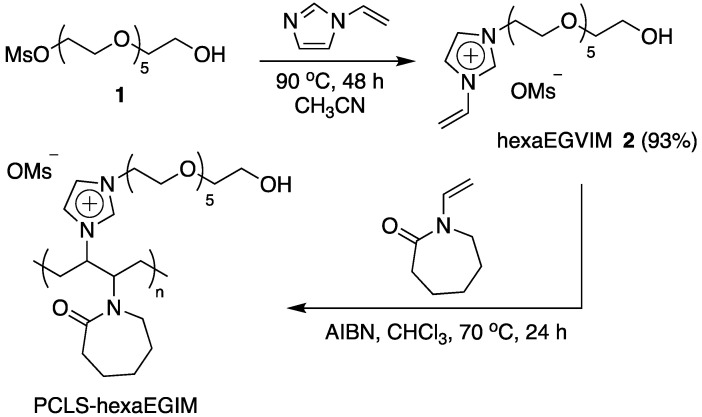
Preparation of PCLS-hexaEGIM.

The imidazolium-based PCLS-hexaEGIM was prepared using a simple and suitable copolymerization reaction using a procedure reported elsewhere (Scheme 1) [28]. The vinyl ionic liquid compound **2** was prepared by reacting 1-vinylimidazole with hexaethylene glycolic mesylate (**1**). The PCLS-hexaEGIM was developed by means of the copolymerization of hexaethylene glycolic vinyl imidazolium mesylate salts (hexaEGVIM **2**) with *N*-vinyl caprolactam in the presence of 2,2′-azobis(2-methylpropionitrile) (AIBN). After synthesis, PCLS-hexaEGIM was characterized using elemental analysis (EA), solid state NMR (see Supplementary Materials, Figure S1), Fourier transform infrared spectroscopy (FT-IR, Figure S2 in Supplementary Materials), X-ray photoelectron spectroscopy (XPS), and thermogravimetric analysis (TGA). The amount of attached IL moiety calculated from the EA result (N 6.38, C 46.26, H 7.24, and S 3.86%) was 3.9 mmol per gram of polymer-supported product obtained, suggesting the successful connection of the caprolactam ring and imidazole rings in polymerization.

**Figure 2:**
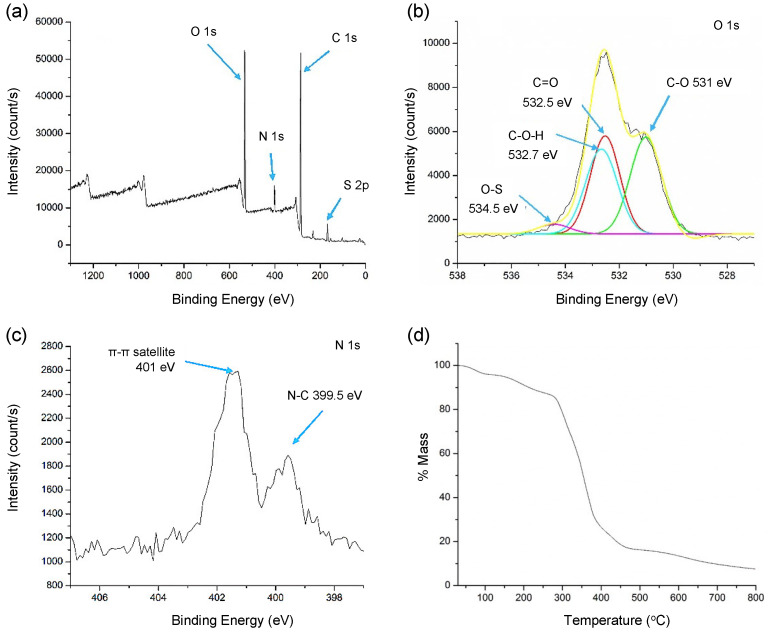
(**a**–**c**) XPS pattern and (**d**) TGA patterns of PCLS-hexaEGIM.

The chemical bonding of PCLS-hexaEGIM was investigated through XPS (Figure 2). The elemental peaks are dominated by O, C, S, and N of PCLS-hexaEGIM (Figure 2a). In particular, Figure 2b shows complete binding between the O atoms and other atoms, such as C, H, and S. Chemical bonding between the monomer brush vinyl ionic liquid hexaEGVIM **2** and monomer *N*-vinyl caprolactam was proven through their peaks. The strong peaks at 532.7 eV (C–O–H bonding) and 531 eV (C–O bonding) were observed for the hexaEGVIM **2** polymer brush. The peak at 532.5 eV (C=O bonding) revealed *N*-vinyl caprolactam after polymerization. The success of this reaction is shown by the new peak at 399.5 eV, which is attributed to N–C bonding (Figure 2c). The peak for N bonding with an aromatic ring (C=C) at 401 eV was attributed to a π–π satellite.

TGA was used to investigate the thermal properties of the final product PCLS-hexaEGIM (Figure 2d). The TGA trace showed that PCLS-hexaEGIM is stable up to 284 °C, which is a high decomposing temperature for ionic catalyst materials. In particular, from 30 °C to 284 °C, the PCLS-hexaEGIM polymer lost 14% of its mass because of the evaporation of absorbed water and mesylate groups. Interestingly, the brushed structure of the polymer (PCLS-hexaEGIM) leads to the high decomposition temperature of mesylate groups, which started from 105 °C. Subsequently, from 284 to 470 °C, the sample lost approximately 70% of its mass due to the high-molecular-weight polymer brush PCLS-hexaEGIM.

To explore the promotive activity of heterogeneous PCLS-hexaEGIM, we investigated the nucleophilic fluorination reaction using mesylate substrate **3** with MFs. This investigation was conducted in the presence of different promoters or catalysts, as detailed in Table 1. First, this study examined the nucleophilic fluorination reaction of **3** in the presence of PCLS-hexaEGIM (0.3 equiv.) with 3 equiv. of CsF in CH_3_CN for 2 h at 90 °C, which provided the desired fluoro-product **4a** with an excellent yield (97%) without forming any appreciable amounts of by-products within 2 h (entry 1). In contrast, the same reaction barely occurred without PCLS-hexaEGIM (entry 2). These findings imply that the terminal hydroxyl group of PCLS-hexaEGIM is regulated through hydrogen bonding with the fluoride from MF. This interaction potentially enhances nucleophilicity through the “flexible” fluoride effect in two ways: (i) the strength of the MF ionic bond might decrease due to hydrogen bonding between the *tert*-alcohol and fluoride in the MF lattice, leading to the selective solvation of fluoride into reaction media. (ii) Limited solvation of fluoride coordinated with bulky *tert*-alcohols may render fluoride an especially potent nucleophile, facilitated by the initial interaction between PCLS-hexaEGIM and CsF [29,30]. Additionally, the PTC effect of the imidazolium salt ionic liquid segment in PCLS-hexaEGIM might further contribute to this process [31]. Subsequently, the effects of various MFs, such as NaF, KF, and RbF, on the nucleophilic fluorination reaction were examined using 0.3 equiv of PCLS-hexaEGIM (entries 3–5, respectively). NaF produced a lower observable product yield, whereas PCLS-hexaEGIM activated KF for the fluorination reaction with an excellent yield, but a prolonged reaction time was required. RbF produced a slightly lower yield than KF.

Next, this study examined the effect of solvents on the model reaction with PCLS-hexaEGIM using polar protic and aprotic solvents. The use of non-polar solvents, such as benzene and 1,4-dioxane, resulted in a lower yield even after a long reaction time (entries 6 and 7, respectively). Conducting the fluorination reaction in polar aprotic DMF provided a good yield (90%) with the formation of an alcohol by-product **4b** (entry 8). In *t*-amyl alcohol, the presence of PCLS-hexaEGIM significantly improved the reaction rate, resulting in a higher yield of the product **4a** (entry 9, 98%) within just 45 min, as compared to the same reaction conducted in the absence of PCLS-hecaEGIM (entry 10). This result indicates that the combined influence of the terminal hydroxyl groups of PCLS-hexaEGIM with *tert*-alcohol media facilitates the formation of “flexible” fluoride, thereby further enhancing the nucleophilicity of fluoride in the reaction. In addition, the efficiency of the promotive activity of PCLS-hexaEGIM was examined with other conventional PTC systems, such as 18-crown-6 in CH_3_CN. The reaction in the presence of 18-crown-6 produced 65% of the desired fluorination product **4a** with a large amount of alkene by-product **4c** formed by an elimination side-reaction facilitated by the “naked” fluoride effect (entry 11) [29,30]. The other heterogeneous catalyst PS[hmim][BF_4_] showed poorer performance than PCLS-hexaEGIM under the same reaction conditions (entry 12). Lastly, the fluorination reaction was performed in the presence of intermediate hexaEGVIM **2** (0.5 equiv.), offering a good yield, but a promoter soluble in solvents and a long reaction time were required (entry 13).

The nucleophilic fluorination of various substrates containing various leaving groups, such as alkyl sulfonate and halides, was performed to expand the scope of the heterogeneous PCLS-hexaEGIM catalyst (Table 2). Despite the challenging nature of effecting nucleophilic fluorination on base-sensitive secondary alkyl bromides using a “naked” fluoride source, the current methodology exhibited favorable results in the synthesis of *sec*-fluoroalkane **5** in CH_3_CN, yielding 75% (entry 1). The *sec*-alkyl fluoride **5** was also obtained from the corresponding *sec*-alkyl mesylate by the PCLS-hexaEGIM-promoted fluorination reaction with CsF in CH_3_CN with an 80% yield (entry 3). Another base-sensitive substrate, 1-(2-mesyloxyethyl)naphthalene, could be converted to 1-(2-fluoroethyl)naphthalene (**6**) with an 80% yield and a smaller amount of the alkene by-products (entry 5). Moreover, these base-sensitive substrates were converted to the corresponding fluoro-products with excellent yields (94–95%) by the PCLS-hexaEGIM promoted fluorination reaction in *t*-amyl alcohol solvent with the minimized formation of alkene by-products (entries 2, 4, and 6). Subsequently, this optimized protocol was explored on various bromo-, iodo-, and mesylate substrates and observed the corresponding fluorinated products **7–9** with a 90–96% yield (entries 7–11). In these reactions, mesylate substrates exhibited slightly faster reaction rates compared to halo-substrates. Interestingly, within 30 min, the nitro-imidazolyl bromide was converted to the desired nitro-imidazolyl fluoride **10** with a 90% yield (entry 12). The fluorination of the sugar mesylate proceeded at 80 °C within 1 h, affording a fluoro-sugar **11** with a 96% yield (entry 13). A 3-fluoro-picoline-*N*-oxide **12** was obtained from the corresponding chloro-substrate with a 92% yield (entry 14). α-Fluoroacetonaphthone **13** was obtained in good yield from the corresponding α-bromoacetonaphthone (entry 15). Finally, a fluoro-estrone **14** was also successfully produced from the corresponding mesylate precursor using this fluorination protocol with a 95% yield (entry 16).

## 3. Materials and Methods

Preparation of 3-(17-hydroxy-3,6,9,12,15-pentaoxaheptadecyl)-1-vinyl-1*H*-imidazol-3-ium methanesulfonate (hexaEGVIM 2). Vinyl-1*H*-imidazole (376 mg, 4.00 mmol) was added dropwise to the solution of 1.44 g (4.00 mmol) of 17-hydroxy-3,6,9,12,15-pentaoxaheptadecyl methanesulfonate in CH_3_CN (50 mL). The reaction mixture was stirred for 24 h at 90 °C. After CH_3_CN was removed under reduced pressure, the residue was washed several times with ethyl acetate (10 mL × 10) and dried under high vacuum overnight at room temperature to obtain 1.6 g (1.87 mmol, 93%) of hexaEGVIM **2** as a white liquid. ^1^H NMR (400 MHz, CDCl_3_) δ 9.54 (s, 1H), 7.73 (dt, *J* = 23.8, 1.8 Hz, 2H), 7.21 (dd, *J* = 15.6, 8.7 Hz, 1H), 5.78 (dd, *J* = 15.6, 2.7 Hz, 1H), 5.24 (dd, *J* = 8.7, 2.7 Hz, 1H), 4.44 (t, *J* = 4.6 Hz, 2H), 3.77 (t, *J* = 4.8 Hz, 2H), 3.65 (s, 1H), 3.56–3.41 (m, 20H), 2.61 (s, 3H); ^13^C NMR (100 MHz, CDCl_3_) δ: 135.7, 128.4, 124.0, 118.6, 108.8, 72.4, 70.1, 70.0, 69.9, 69.8, 69.7, 68.5, 60.8, 49.5, 39.2. HRMS (EI, *m*/*z*): calcd for C_18_H_34_N_2_O_9_S 454.1985, found: 454.1980.

Preparation of poly caprolactam supported hexaethylene glycol substituted imidazolium salts (PCLS-hexaEGIM). The mixture solution of *N*-vinyl caprolactam (5.0 g, 35 mmol), hexaEGVIM **2** (2.0 g, 4.4 mmol), and AIBN (100 mg, 0.6 mmol) in CH_2_Cl_2_ (50 mL) was placed in a round-bottom flask. The polymerization reaction was performed at 70 °C for 24 h under N_2_. After completion of the reaction, the PCLS-hexaEGIM (4.2 g) was collected by means of simple filtration, washing with acetone (250 mL × 3) and methanol (250 mL × 3), and drying under high vacuum at 50 °C overnight.

Typical procedure of nucleophilic fluorination in Table 1 (entry 1). CsF (456 mg, 3 mmol) was added to the mixture of 2-(3-methanesulfonyloxypropoxy)naphthalene (**3**, 281 mg, 1.0 mmol) and PCLS-hexaEGIM (279 mg, 0.3 mmol) in CH_3_CN (4 mL) in a reaction vial. The reaction mixture was stirred for 2 h at 90 °C. The reaction time was determined by checking TLC. The reaction mixture was filtered and washed with diethyl ether, and the filtrate was evaporated under reduced pressure. Flash column chromatography (10% EtOAc/hexanes) of the filtrate afforded 192 mg (0.94 mmol, 94%) of 2-(3-fluoropropoxy)naphthalene (**4a**) as a white solid; ^1^H NMR (400 MHz, CDCl_3_) ^1^H NMR (400 MHz, CDCl_3_) δ 7.73 (q, *J* = 8.2 Hz, 3H), 7.42 (td, *J* = 7.5, 1.1 Hz, 1H), 7.32 (td, *J* = 7.5, 1.4 Hz, 1H), 7.15–7.11 (m, 2H), 4.68 (dt, *J* = 47.2, 5.7 Hz, 2H), 4.21 (t, *J* = 6.2 Hz, 2H), 2.29–2.14 (m, 2H); ^13^C NMR (100 MHz, CDCl_3_) δ 156.6, 134.5, 129.4, 129.0, 127.6, 126.7, 126.3, 123.6, 118.7, 106.6, 80.7 (d, *J* = 163.8 Hz), 63.5 (d, *J* = 5.8 Hz) 30.4 (d, *J* = 19.2 Hz). HRMS (EI, *m*/*z*): calcd for C_13_H_13_FO 204.0950, found: 204.0949.

### 3.1. Procedure of Nucleophilic Fluorinations in Table 1

Procedure of entry 2. Prepared according to the typical procedure of fluorination except for not using a promoter. This reaction provided 20 mg of **4a** with a 10% yield after 10 h.

Procedure of entry 3. Prepared according to the typical procedure of fluorination except for using NaF (126 mg, 3 mmol) for 24 h instead of using CsF. This reaction provided 41 mg of **4a** with a 20% yield.

Procedure of entry 4. Prepared according to the typical procedure of fluorination except for using KF (174 mg, 3 mmol) instead of using CsF. This reaction provided 192 mg of **4a** with a 94% yield after 10 h.

Procedure of entry 5. Prepared according to the typical procedure of fluorination except for using RbF (314 mg, 3 mmol) instead of using CsF. This reaction provided 188 mg of **4a** with a 92% yield after 9 h.

Procedure of entry 6. Prepared according to the typical procedure of fluorination except for using benzene (4 mL) instead of using CH_3_CN. This reaction provided 174 mg of **4a** with a 85% yield after 12 h.

Procedure of entry 7. Prepared according to the typical procedure of fluorination except for using 1,4-dioxane (4 mL) instead of using CH_3_CN. This reaction provided 180 mg of **4a** with a 88% yield after 12 h.

Procedure of entry 8. Prepared according to the typical procedure of fluorination except for using DMF (4 mL) instead of using CH_3_CN. This reaction provided 184 mg of **4a** with a 90% yield after 3 h

Procedure of entry 9. Prepared according to the typical procedure of fluorination except for using *tert*-amyl alcohol (4 mL) instead of using CH_3_CN. This reaction provided 200 mg of **4a** in 98% yield within 45 min

Procedure of entry 11. Prepared according to the typical procedure of fluorination except for using 18-crown-6 (264 mg, 1.0 mmol) for 8 h instead of using PCLS-hexaEGIM (279 mg, 0.3 mmol). This reaction provided 133 mg of **4a** with a 65% yield.

Procedure of entry 12. Prepared according to the typical procedure of fluorination except for using PS[hmim][BF_4_] (415 mg, 0.55 mmol) for 5 h instead of using PCLS-hexaEGIM. This reaction provided 192 mg of **4a** with a 94% yield.

Procedure of entry 13. Prepared according to the typical procedure of fluorination except for using [hexaEGvim][OMs] (457 mg, 1.0 mmol) for 5 h instead of using PCLS-hexaEGIM. This reaction provided 188 mg of **4a** with a 92% yield.

### 3.2. Procedure of Fluorination of Various Substrates and Analytical Data of Fluorination Products in Table 2

2-(2-fluoropropoxy)naphthalene (5, entry 1). Prepared according to the typical procedure of fluorination (entry 1 in Table 1) except for using 2-(2-bromopropoxy)naphthalene (264 mg, 1.0 mmol) instead of using mesylate **3**. This reaction provided 154 mg (0.75 mmol, 95%) of 2-(2-fluoropropoxy)naphthalene (**5**) in 4 h; ^1^H-NMR (400 MHz, CDCl_3_) δ 7.78–7.69 (m, 3H), 7.47–7.40 (m, 1H), 7.37–7.31 (m, 1H), 7.19 (dd, *J* = 8.9, 2.5 Hz, 1H), 7.12 (d, *J* = 2.3 Hz, 1H), 5.18–4.97 (m, 1H), 4.24–4.06 (m, 2H), 1.50 (dd, *J* = 23.6, 6.4 Hz, 3H); ^13^C NMR (100 MHz, CDCl_3_) δ 156.4, 134.4, 129.5, 129.1, 127.6, 126.7, 126.4, 123.8, 118.8, 106.7, 88.4 (d, *J* = 168.7 Hz), 70.7 (d, *J* = 23.0 Hz), 17.4 (d, *J* = 22.0 Hz). HRMS (EI, *m*/*z*): calcd for C_13_H_13_FO 204.0950 found: 204.0952.

1-(2-Fluoroethyl)naphthalene (6, entry 5). Prepared according to the typical procedure of fluorination except for using 2-(naphthalen-1-yl)ethyl methanesulfonate (250 mg, 1.0 mmol) instead of using mesylate **3**. This reaction provided 140 mg (0.80 mmol, 80%) of 1-(2-fluoroethyl)naphthalene (**6**) in 2 h;^1^H-NMR (400 MHz, CDCl_3_) δ 8.03 (d, *J* = 8.2 Hz, 1H), 7.88 (d, *J* = 7.8 Hz, 1H), 7.78 (d, *J* = 7.8 Hz, 1H), 7.58–7.38 (m, 4H), 4.77 (dt, *J* = 46.8, 7.0 Hz, 2H), 3.51 (td, *J* = 13.7, 6.7 Hz, 2H);^13^C NMR (100 MHz, CDCl_3_) δ 133.8, 132.8, 132.7, 131.9, 128.8, 127.5, 127.1, 126.1, 125.6, 125.5, 123.3, 83.5, (d, *J* = 168 Hz), 33.8, (d, *J* = 20 Hz). HRMS (EI, *m*/*z*): calcd for C_12_H_11_F 174.0845, found: 174.0843.

4-(3-fluoropropoxy)-2*H*-chromen-2-one (7, entry 8). Prepared according to the typical procedure of fluorination except for using 3-((2-oxo-2H-chromen-4-yl)oxy)propyl methanesulfonate (298 mg, 1.0 mmol) instead of using mesylate **3**. This reaction provided 207 mg (0.93 mmol, 93%) of 4-(3-fluoropropoxy)-2*H*-chromen-2-one (**7**) in 2 h;^1^H-NMR (400 MHz, CDCl_3_) δ 7.79 (dd, *J* = 8.0, 1.6 Hz, 1H), 7.58–7.51 (m, 1H), 7.33–7.23 (m, 2H), 5.69 (s, 1H), 4.67 (dt, *J* = 47.2, 5.7 Hz, 2H), 4.27 (t, *J* = 5.9 Hz, 2H), 2.36–2.22 (m, 2H); ^13^C NMR (100 MHz, CDCl_3_) δ 165.2, 162.6, 132.3, 123.8, 122.7, 116.7, 115.4, 90.6, 90.6, 80.4, (d, *J* = 165 Hz), 65.0, (d, *J* = 4.8 Hz), 29.6, (d, *J* = 20 Hz). HRMS (EI, *m*/*z*): calcd for C_12_H_11_FO_3_ 222.0692, found: 222.0693.

1,17-difluoro-3,6,9,12,15-pentaoxaheptadecane (8, entry 10). Prepared according to the typical procedure of fluorination except for using 1,17-dibromo-3,6,9,12,15-pentaoxaheptadecane (408 mg, 1.0 mmol) instead of using mesylate **3**. This reaction provided 267 mg (0.94 mmol, 94%) of 1,17-difluoro-3,6,9,12,15-pentaoxaheptadecane (**8**) in 1 h; ^1^H-NMR (400 MHz, CDCl_3_) δ 4.62–4.57 (m, 2H), 4.50–4.45 (m, 2H), 3.77–3.74 (m, 1H), 3.70–3.60 (m, 19H);^13^C NMR (100 MHz, CDCl_3_) δ 83.0, (d, *J* = 167 Hz), 70.7, 70.6, 70.5, 70.4, 70.2. HRMS (FAB, *m*/*z*): calcd for C_12_H_25_F_2_O_5_ (M + H^+^) 287.1670, found: 287.1672 (M + H^+^).

1-(3-fluoropropoxy)-4-nitrobenzene (9, entry 11). Prepared according to the typical procedure of fluorination except for using 3-(4-nitrophenoxy)propyl methanesulfonate (275 mg, 1.0 mmol) instead of using mesylate **3**. This reaction provided 191 mg (0.96 mmol, 96%) of 1-(3-fluoropropoxy)-4-nitrobenzene (**9**) in 45 min; ^1^H-NMR (400 MHz, CDCl_3_) δ 8.20–8.14 (m, 2H), 6.94 (dt, *J* = 10.2, 2.7 Hz, 2H), 4.63 (dt, *J* = 46.8, 5.7 Hz, 2H), 4.18 (t, *J* = 6.2 Hz, 2H), 2.26–2.11 (m, 2H); ^13^C NMR (100 MHz, CDCl_3_) δ 163.7, 141.5, 125.8, 114.6, 114.3, 81.2 (d, *J* = 164 Hz), 64.3 (d, *J* = 5 Hz), 30.0 (d, *J* = 20 Hz). HRMS (EI, *m*/*z*): calcd for C_9_H_10_FNO_3_ 199.0645, found: 199.0646.

1-(3-Fluoropropyl)-4-nitroimidazole (10, entry 12). Prepared according to the typical procedure of fluorination except for using 1-(3-bromopropyl)-4-nitroimidazole (234 mg, 1.0 mmol) instead of using mesylate **3**. This reaction provided 156 mg (0.90 mmol, 90%) of 1-(3-fluoropropyl)-4-nitroimidazole (**10**) in 30 min; ^1^H-NMR (400 MHz, CDCl_3_) δ 7.79 (d, *J* = 1.4 Hz, 1H), 7.45 (d, *J* = 1.4 Hz, 1H), 4.45 (dt, *J* = 47.2, 5.5 Hz, 2H), 4.20 (t, *J* = 6.9 Hz, 2H), 2.28–2.13 (m, 2H); ^13^C NMR (100 MHz, CDCl_3_) δ 148.2, 136.1, 119.2, 79.6, (d, *J* = 166 Hz), 44.3, (d, *J* = 164 Hz), 31.2, (d, *J* = 20 Hz). HRMS (EI, *m*/*z*): calcd for C_6_H_8_FN_3_O_2_ 173.0601, found: 173.0603.

1,2:3,4-Di-*O*-isopropylidene-6-fluoro-6-deoxy-α-D-galactopyranose (11, entry 13). Prepared according to the typical procedure of fluorination except for using 1,2:3,4-Di-*O*-isopropylidene-6-methanesulfonate-6-deoxy-α-D-galactopyranose (338 mg, 1.0 mmol) instead of using mesylate **3**. This reaction provided 252 mg (0.96 mmol, 96%) of 1,2:3,4-Di-*O*-isopropylidene-6-fluoro-6-deoxy-α-D-galactopyranose (**11**) in 1 h; ^1^H-NMR (400 MHz, CDCl_3_) δ 5.53 (d, *J* = 5.0 Hz, 1H), 4.65–4.54 (m, 2H), 4.53–4.41 (m, 1H), 4.32 (q, *J* = 2.6 Hz, 1H), 4.27–4.22 (m, 1H), 4.09–4.00 (m, 1H), 1.52 (s, 3H), 1.42 (s, 3H), 1.32 (s, 6H); ^13^C NMR (100 MHz, CDCl_3_) δ 109.6, 108.7, 96.1, 82.0 (d, *J* = 167 Hz), 70.5 (d, *J* = 9Hz), 66.4 (d, *J* = 23 Hz), 26.0, 25.9, 24.8, 24.4. HRMS (FAB, *m*/*z*): calcd for C_12_H_20_FO_5_ (M + H^+^) 263.1295, found: 263.1293 (M + H^+^).

3-Fluoro-picoline *N*-oxide (12, entry 14). Prepared according to the typical procedure of fluorination except for using 3-chloro-picoline *N*-oxide (143 mg, 1.0 mmol) instead of using mesylate **3**. This reaction provided 117 mg (0.92 mmol, 92%) of 3-fluoro-picoline *N*-oxide (**12**) in 1 h; ^1^H-NMR (400 MHz, CDCl_3_) δ 8.21 (d, *J* = 0.9 Hz, 1H), 8.15 (t, *J* = 5.7 Hz, 1H), 7.31–7.20 (m, 2H), 5.34 (d, *J* = 47.2 Hz, 2H); ^13^C NMR (100 MHz, CDCl_3_) δ 139.0, 137.5, 137.4, 135.9, 135.7, 125.9, 124.0, 123.9, 80.4, (d, *J* = 171 Hz). HRMS (EI, *m*/*z*): calcd for C_6_H_6_FNO 127.0433, found: 127.0431.

2-Fluoro-2′-acetonaphthone (13, entry 15). Prepared according to the typical procedure of fluorination except for using 2-bromo-1-(naphthalen-2-yl)ethan-1-one (247 mg, 1.0 mmol) instead of using mesylate **3** (281 mg, 1.0 mmol). This reaction provided 173 mg (0.92 mmol, 92%) of 2-fluoro-2′-acetonaphthone (**13**) in 1 h; ^1^H-NMR (400 MHz, CDCl_3_) δ 8.39 (s, 1H), 7.97–7.85 (m, 4H), 7.59 (m, 2H), 5.64 (d, *J* = 46.4 Hz, 2H); ^13^C NMR (100 MHz, CDCl_3_) δ 193.0, 135.9, 132.3, 129.8, 129.6, 129.0, 128.9, 127.9, 127.1, 123.2, 83.5, (d, *J* = 181 Hz), HRMS (EI, *m*/*z*) calcd for C_12_H_9_FO 188.0637, found: 188.0638.

3-*O*-(3-Fluoropropyl)estrone (14, entry 16). Prepared according to the typical procedure of fluorination except for using 3-*O*-(3-methanesulfonylpropyl)estrone (390 mg, 1.0 mmol) instead of using mesylate **3**. This reaction provided 316 mg (0.95 mmol, 95%) of 3-*O*-(3-fluoropropyl)estrone (**14**) in 3 h; ^1^H-NMR (400 MHz, CDCl_3_) δ 7.21–7.16 (m, 1H), 6.70 (dd, *J* = 8.7, 2.7 Hz, 1H), 6.64 (d, *J* = 2.7 Hz, 1H), 4.62 (t, *J* = 47.2, 5.9 Hz, 2H), 4.06 (t, *J* = 6.2 Hz, 2H), 2.88 (m, 2H), 2.47–2.30 (m, 2H), 2.28–1.89 (m, 7H), 1.66–1.36 (m, 6H), 0.89 (s, 3H); ^13^C NMR (100 MHz, CDCl_3_) δ 220.8, 156.7, 132.7, 132.2, 126.3, 114.5, 112.1, 80.75 (d, *J* = 164 Hz), 63.4, (d, *J* = 4.8 Hz) 50.3, 47.9, 43.9, 38.3, 35.8, 31.5, 30.4 (d, *J* = 20.2 Hz), 29.6, 26.5, 21.5, 13.8. HRMS (EI) *m*/*z* calcd for C_21_H_27_FO_2_, 330.1995, found: 330.1992.

## 4. Conclusions

In summary, PCLS-hexaEGIM was developed as a polymer-grafted heterogeneous PTC promoter and demonstrated its high efficacy in S*_N_*2 fluorination of various substrates using alkali metal fluorides. In this protocol, PCLS-hexaEGIM enhances the nucleophilicity of MFs significantly while also offering practical advantages such as straightforward product purification. These factors are technically attractive when considering the use of this protocol for industrial processes. Moreover, significant efficacy was observed via the synergistic combination of PCLS-hexaEGIM with *tert*-alcohol solvent in the chemo-selective fluorinations of base-sensitive substrates. This protocol can be suitable for other IL syntheses on highly cross-linked polymer matrices, which are stable and insoluble in organic solvents with the desired functional groups.

## Figures and Tables

**Table 1:**
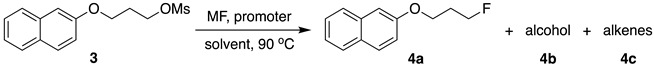
Nucleophilic fluorination of mesylate **3** with MF in the presence of promoter under various reaction conditions ^a^.

Entry	Promoter (Equiv)	Solvent	MF	Time (h)	Yield ^b^			
					3	4a	4b	4c
1	PCLS-hexaEGIM (0.3)	CH_3_CN	CsF	2	-	97 (94) ^c^	-	-
2	-	CH_3_CN	CsF	10	90	10	-	-
3	PCLS-hexaEGIM (0.3)	CH_3_CN	NaF	24	75	20	5	-
4	PCLS-hexaEGIM (0.3)	CH_3_CN	KF	10	-	94	5	-
5	PCLS-hexaEGIM (0.3)	CH_3_CN	RbF	9	3	92	5	-
6	PCLS-hexaEGIM (0.3)	Benzene	CsF	12	-	85	15	-
7	PCLS-hexaEGIM (0.3)	1,4-dioxane	CsF	12	-	88	12	-
8	PCLS-hexaEGIM (0.3)	DMF	CsF	3	-	90	10	-
9	PCLS-hexaEGIM (0.3)	t-amyl alcohol	CsF	45 min	-	98	-	-
10 ^d^	-	t-amyl alcohol	CsF	2.5	-	94	-	-
11	18-crown-6 (0.5)	CH_3_CN	CsF	8	10	65	5	25
12	PS[hmim][BF_4_] (0.5)	CH_3_CN	CsF	5		94	-	6
13	hexaEGVIM **2** (0.5)	CH_3_CN	CsF	5	-	92	-	8

^a^All reactions were carried out on a 1.0 mmol scale of mesylate **3** using 3 equiv. of MF in the presence of promoter in 4.0 mL of solvent at 90 °C. ^b^ Yields were determined by means of ^1^H NMR spectroscopy. ^c^ Isolated yield. ^d^ Reference [29].

**Table 2:**
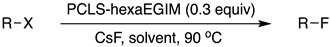
Substrate scope of PCLS-hexaEGIM-promoted nucleophilic fluorination reactions ^a^.

Entry	Structure of Product	X	Solvent	Time (h)	Yield (%) ^b^	Comments
1	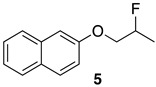	Br	CH_3_CN	4	75 ^c^	15% alkenes 10% alcohol
2 ^d^		Br	t-amyl alcohol	2	94 ^c^	3% alkenes trace alcohol
3		OMs	CH_3_CN	3	80 ^c^	20% alkenes
4		OMs	t-amyl alcohol	1.5	95 ^c^	5% alkenes
5	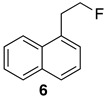	OMs	CH_3_CN	2	80 ^c^	15% alkenes 5% alcohol
6		OMs	t-amyl alcohol	0.5	95 ^c^	5% alkenes
7	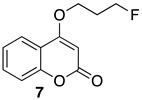	OMs	CH_3_CN	2	93	4% alcohol
8		Br	CH_3_CN	3	90	6% alkenes
9		I	CH_3_CN	3	91	4% alkenes
10	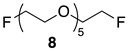	Br	CH_3_CN	1	94	-
11	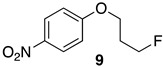	OMs	CH_3_CN	0.75	96	-
12	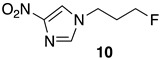	Br	CH_3_CN	0.5	90	4% alcohol
13 ^e^	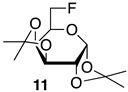	OMs	CH_3_CN	1	96	-
14 ^e^	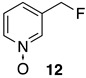	Cl	CH_3_CN	1	92	trace alcohol
15	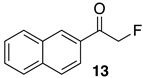	Br	CH_3_CN	1	92	4% alcohol
16	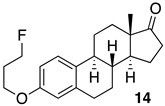	OMs	CH_3_CN	3	95	-

^a^ All reactions were carried out on a 1.0 mmol scale of substrate using 3 equiv. of CsF in the presence of PCLS-hexaEGIM (0.3 equiv) in 4.0 mL of solvent at 90 °C. ^b^ Yield of isolated product. ^c^ Yields determined by means of ^1^H NMR spectroscopy. ^d^ The reaction was carried out at 100 °C and ^e^ at 80 °C.

## Data Availability

Not applicable.

## Supplementary Materials

The following supporting information can be downloaded at: https://www.mdpi.com/article/10.3390/molecules28186747/s1, Figure S1: Solid state 1H NMR spectrum of PCLS-hexaEGIM. Figure S2: FT-IR spectrum of PCLS-hexaEGIM.
