# Supplementary material for: Poly Caprolactam Supported Hexaethylene Glycolic Imidazolium Ionic Liquid as a Heterogeneous Promoter for Nucleophilic Fluorination
Source: Molecules. 2023 Sep 21;28(18):6747. doi: 10.3390/molecules28186747 (PMC10535173; doi:10.3390/molecules28186747)

Supplementary Information

Poly Caprolactam Supported Hexaethylene Glycolic Imidazolium Ionic Liquid as a Heterogeneous Promoter for Nucleophilic Fluorination

1. *Preparation of PCLS-hexaEGIM*

Preparation of 3-(17-hydroxy-3,6,9,12,15-pentaoxaheptadecyl)-1-vinyl-1*H*-imidazol-3-ium methanesulfonate (hexaEGVIM 2). Vinyl-1*H*-imidazole (376 mg, 4.00 mmol) was added dropwise to the solution of 1.44 g (4.00 mmol) of 17-hydroxy-3,6,9,12,15-pentaoxaheptadecyl methanesulfonate in CH_3CN (50 mL). The reaction mixture was stirred for 24 h at 90 °C. After CH_3CN was removed under reduced pressure, the residue was washed several times with ethyl acetate (10 mL × 10) and dried under high vacuum overnight at room temperature to obtain 1.6 g (1.87 mmol, 93%) of hexaEGVIM **2** as a white liquid. ^{1}H-NMR (400 MHz, $CDCl_3$) δ 9.54 (s, 1H), 7.73 (dt, *J* = 23.8, 1.8 Hz, 2H), 7.21 (dd, *J* = 15.6, 8.7 Hz, 1H), 5.78 (dd, *J* = 15.6, 2.7 Hz, 1H), 5.24 (dd, *J* = 8.7, 2.7 Hz, 1H), 4.44 (t, *J* = 4.6 Hz, 2H), 3.77 (t, *J* = 4.8 Hz, 2H), 3.65 (s, 1H), 3.56-3.41 (m, 20H), 2.61 (s, 3H); ^{13}C NMR (100 MHz, $CDCl_3$) δ: 135.7, 128.4, 124.0, 118.6, 108.8, 72.4, 70.1, 70.0, 69.9, 69.8, 69.7, 68.5, 60.8, 49.5, 39.2. HRMS (EI, m/z): calcd for $C_{18}H_{34}N_2O_9S$ 454.1985, found: 454.1980.

Preparation of poly caprolactam supported hexaethylene glycol substituted imidazolium salts (PCLS-hexaEGIM). The mixture solution of *N*-vinyl caprolactam (5.0 g, 35 mmol), hexaEGVIM **2** (2.0 g, 4.4 mmol), and AIBN (100 mg, 0.6 mmol) in CH_2Cl_2 (50 mL) was placed in around bottom flask. The polymerization reaction was performed at 70 °C for 24 h under N_2. After completion of the reaction, the PCLS-hexaEGIM (4.2 g) was collected by

simple filtration, washing with acetone (250 mL × 3) and methanol (250 mL × 3), and drying under high vacuum at 50 °C overnight.

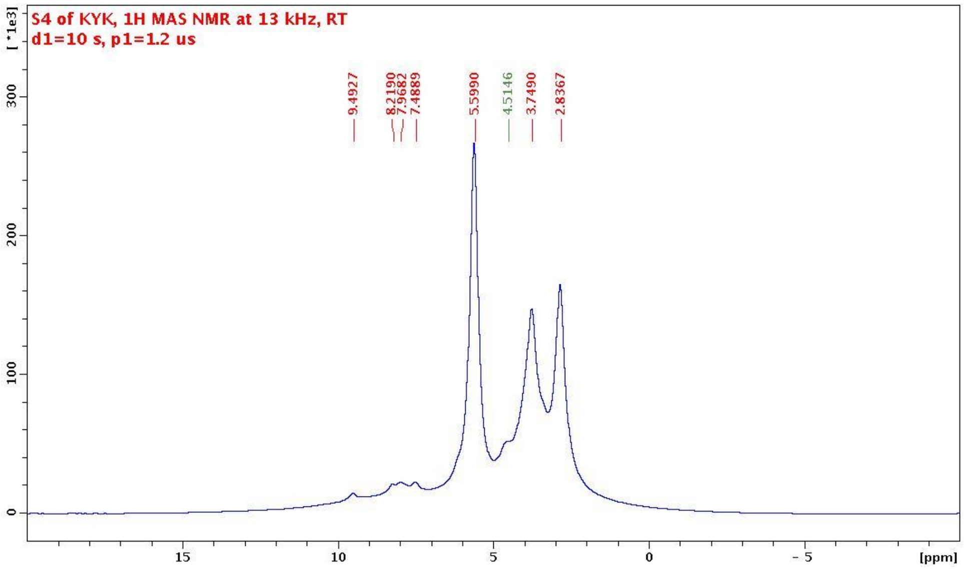

Figure S1. Solid state 1H NMR spectrum of PCLS-hexaEGIM

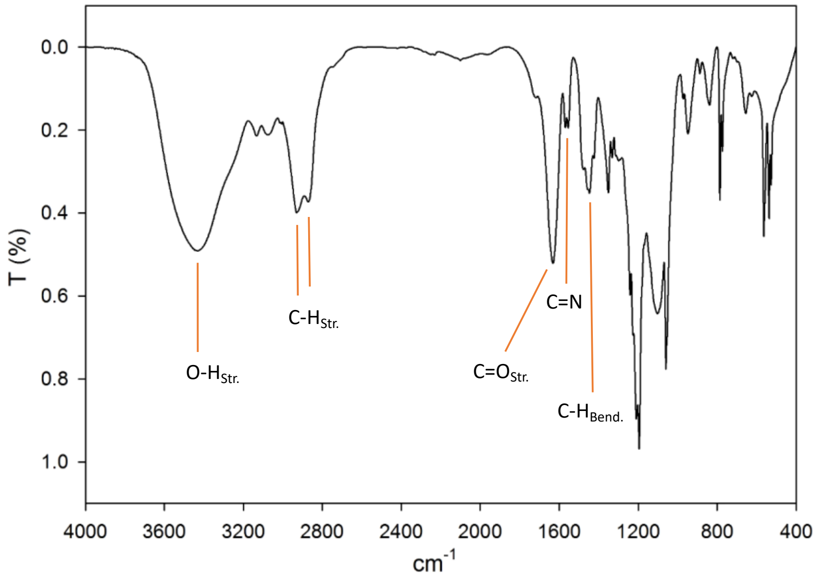

Figure S2. FT-IR spectrum of PCLS-hexaEGIM.

In the FT-IR spectrum of PCLS-hexaEGIM (Figure 2), the hydroxy group of ethylene glycol at the side chain was observed as a broad peak at 3429.7 cm^{-1}. All the C–H symmetric and asymmetric stretching and bending vibrations appeared at 2927.2, 2890.4, and 1449.7

cm^{-1}, respectively. In this spectrum, the lactone N–H vibration disappeared, and the carbonyl (C=O) stretching vibrations of the lactone ring and the imidazole C=N stretching vibrations were observed at 1629.7 and 1562.8 cm^{-1}, respectively, indicating the successful connection of the lactone ring and imidazole rings in polymerization. All the remaining absorption peaks, such as C–N, mesylate S=O, and S–O stretching vibrations, appear at their relative absorption area.

2. *Typical procedure of the fluorination.*

Typical procedure of nucleophilic fluorination in Table 1 (entry 1). CsF (456 mg, 3 mmol) was added to the mixture of 2-(3-methanesulfonyloxypropoxy)naphthalene (**3**, 281 mg, 1.0 mmol) and PCLS-hexaEGIM (279 mg, 0.3 mmol) in CH_3CN (4 mL) in a reaction vial. The reaction mixture was stirred over 2 h at 90 ºC. The reaction time was determined by checking TLC. The reaction mixture was filtered and washed with diethyl ether, and the filtrate was evaporated under reduced pressure. Flash column chromatography (10% EtOAc/hexanes) of the filtrate afforded 192 mg (0.94 mmol, 94%) of 2-(3-fluoropropoxy)naphthalene (**4a**) as a white solid; ^{1}H NMR (400 MHz, $CDCl_3$) ^{1}H-NMR (400 MHz, $CDCl_3$) δ 7.73 (q, *J* = 8.2 Hz, 3H), 7.42 (td, *J* = 7.5, 1.1 Hz, 1H), 7.32 (td, *J* = 7.5, 1.4 Hz, 1H), 7.15-7.11 (m, 2H), 4.68 (dt, *J* = 47.2, 5.7 Hz, 2H), 4.21 (t, *J* = 6.2 Hz, 2H), 2.29-2.14 (m, 2H); ^{13}C NMR (100 MHz, $CDCl_3$) δ 156.6, 134.5, 129.4, 129.0, 127.6, 126.7, 126.3, 123.6, 118.7, 106.6, 80.7 (d, *J* = 163.8 Hz), 63.5 (d, *J* = 5.8 Hz) 30.4 (d, *J* = 19.2 Hz). HRMS (EI, m/z): calcd for $C_{13}H_{13}FO$ 204.0950, found: 204.0949.

Spectral data of intermediates, HexaEGVIM 2.

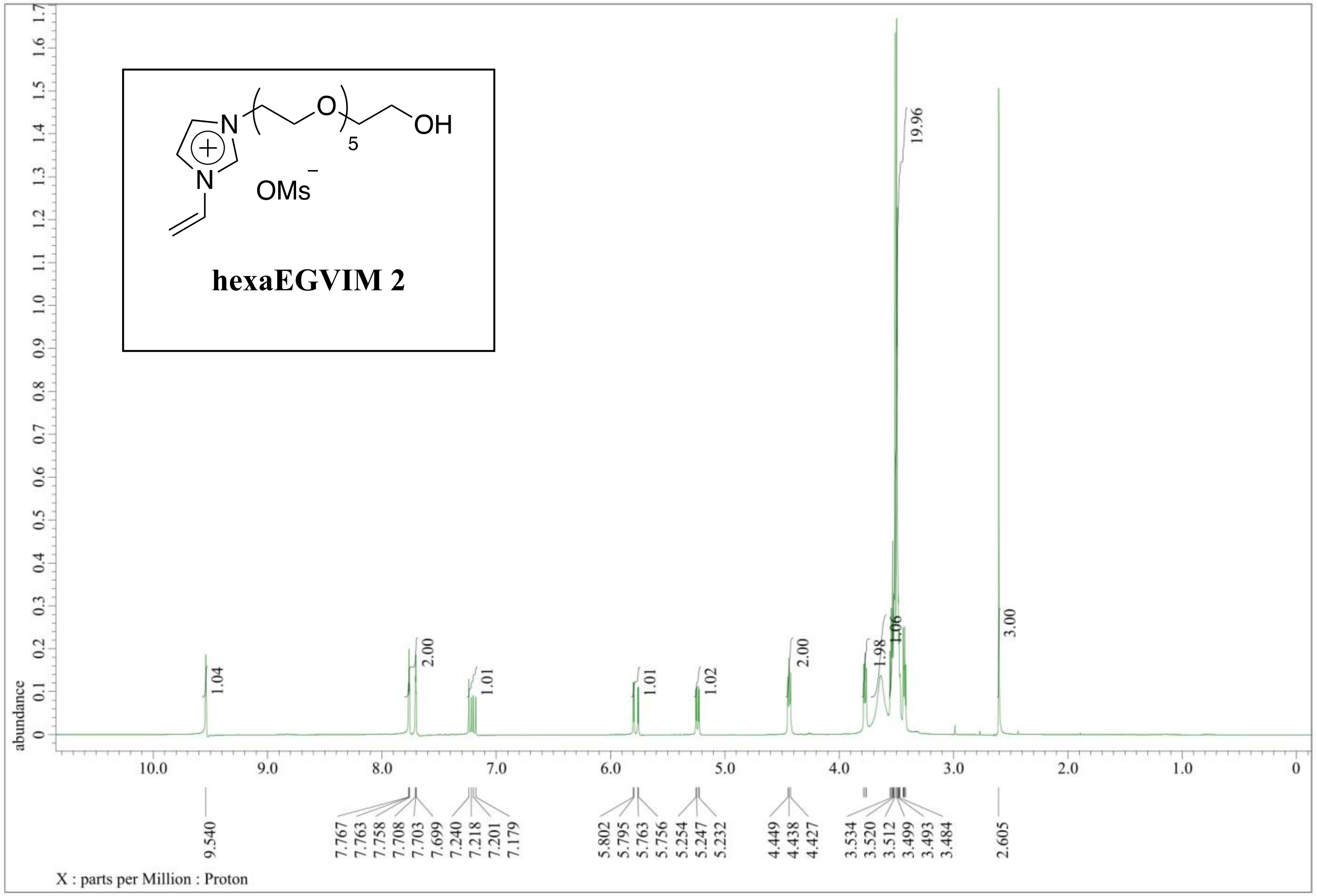

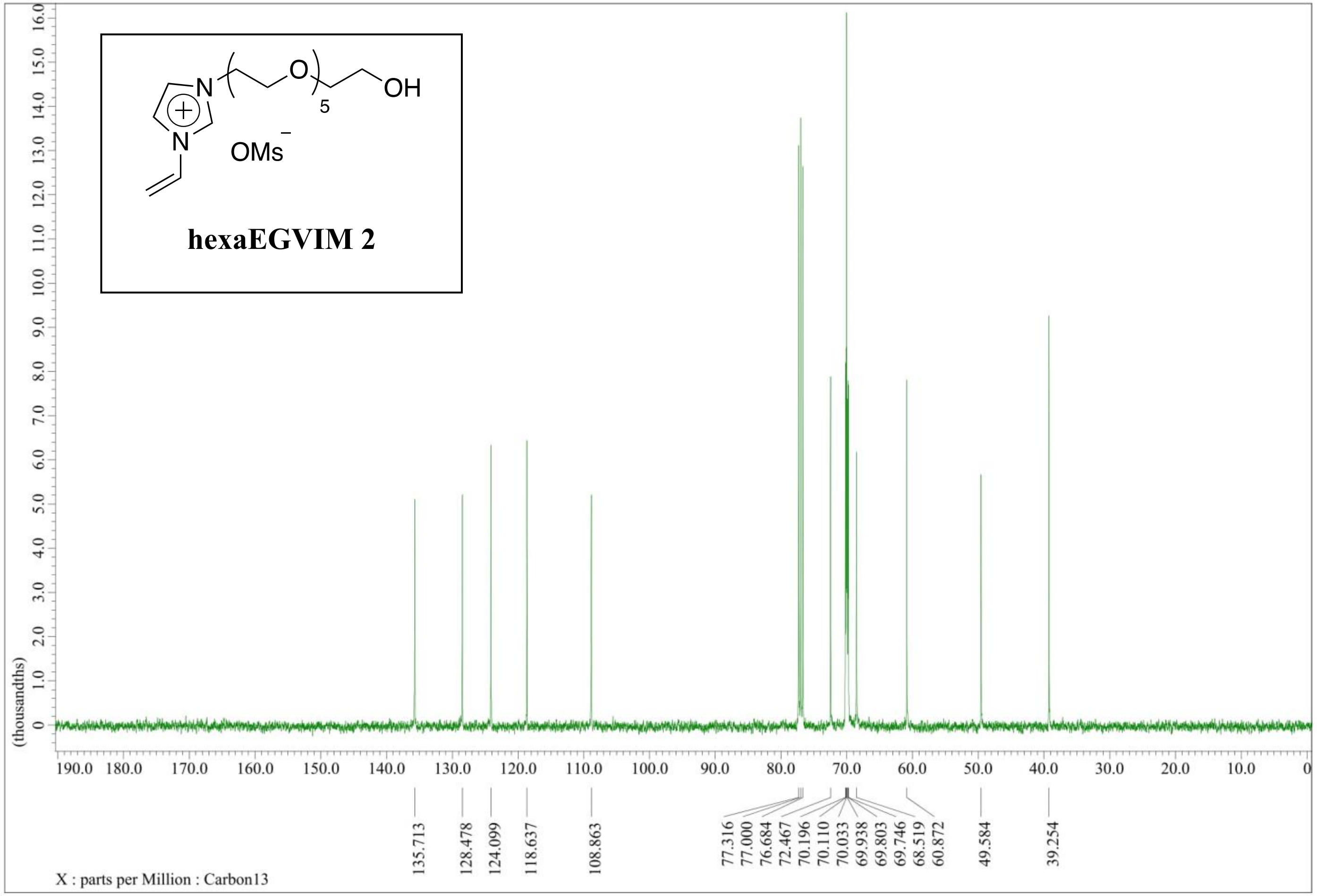

N
O
5
OH
N
OMs⁻
hexaEGVIM 2
(thousandths)
X : parts per Million : Carbon13
135.713
128.478
124.099
118.637
108.863
77.316
77.000
76.684
72.467
70.196
70.110
70.033
69.938
69.803
69.746
68.519
60.872
49.584
39.254

*^{1}H-NMR- spectra of 2-(3-Fluoropropoxy)naphthalene (**4a**).*

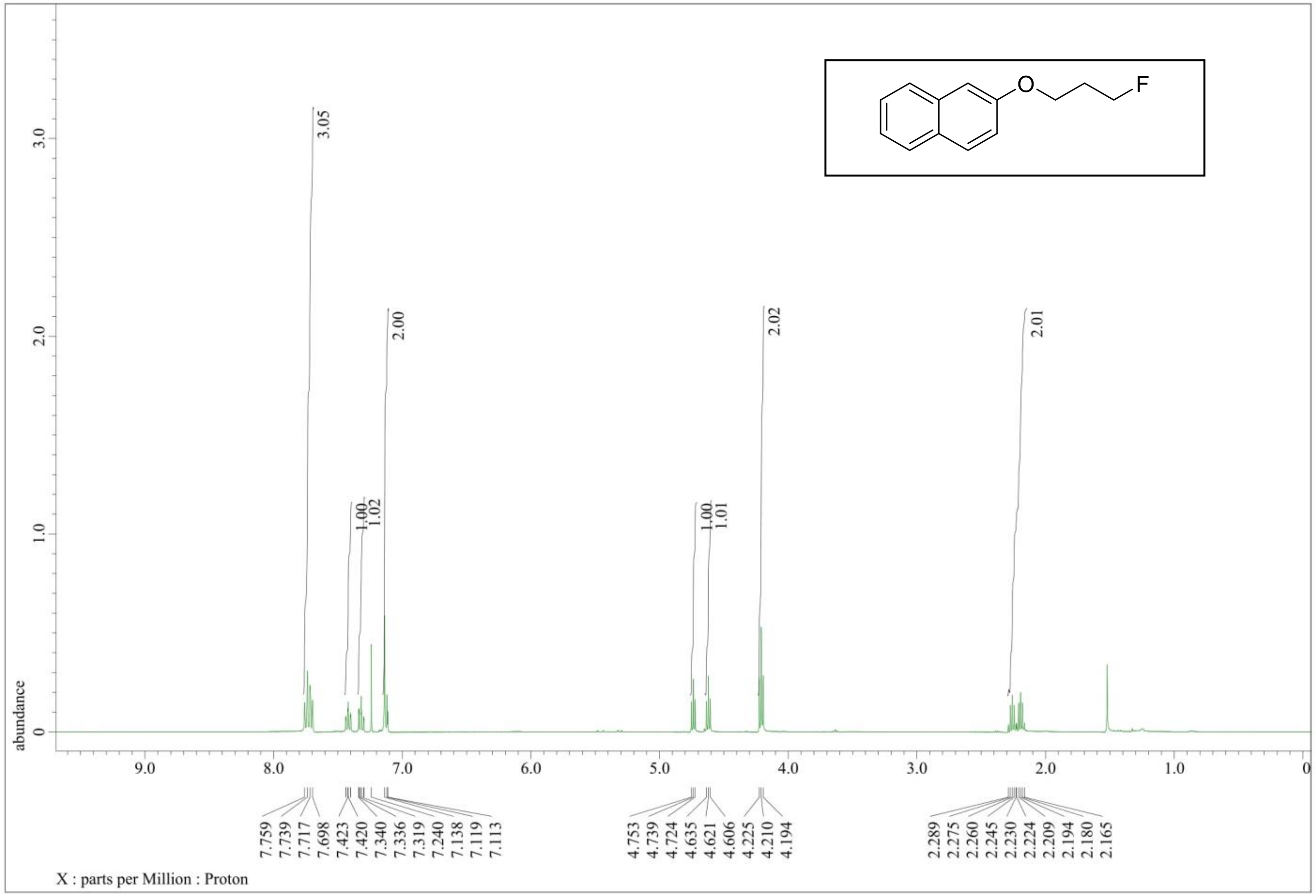

*[31]C –NMR-spectra of 2-(3-Fluoropropoxy)naphthalene (**4a**).*

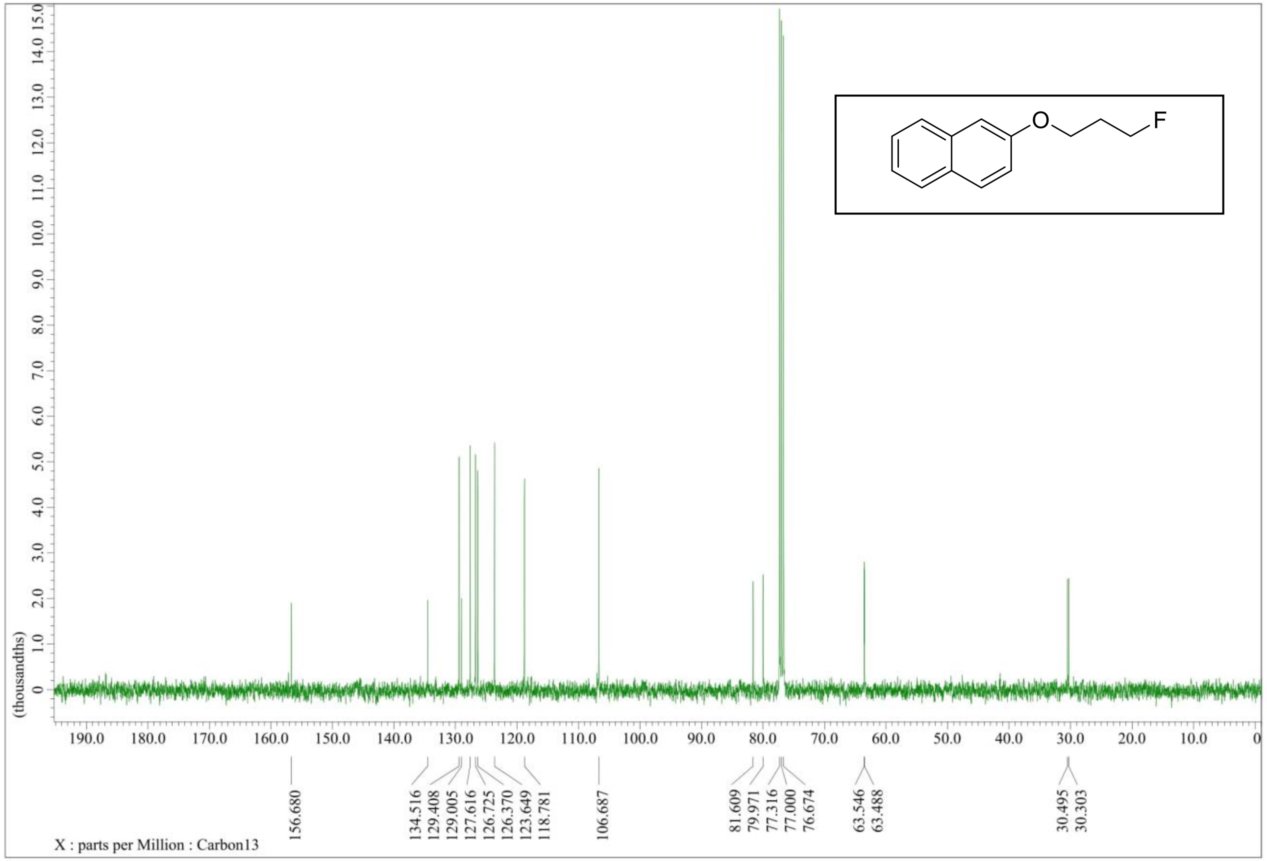

^{1}H and ^{13}C NMR spectra of products in Table 2.

2-(2-fluoropropoxy)naphthalene (5).

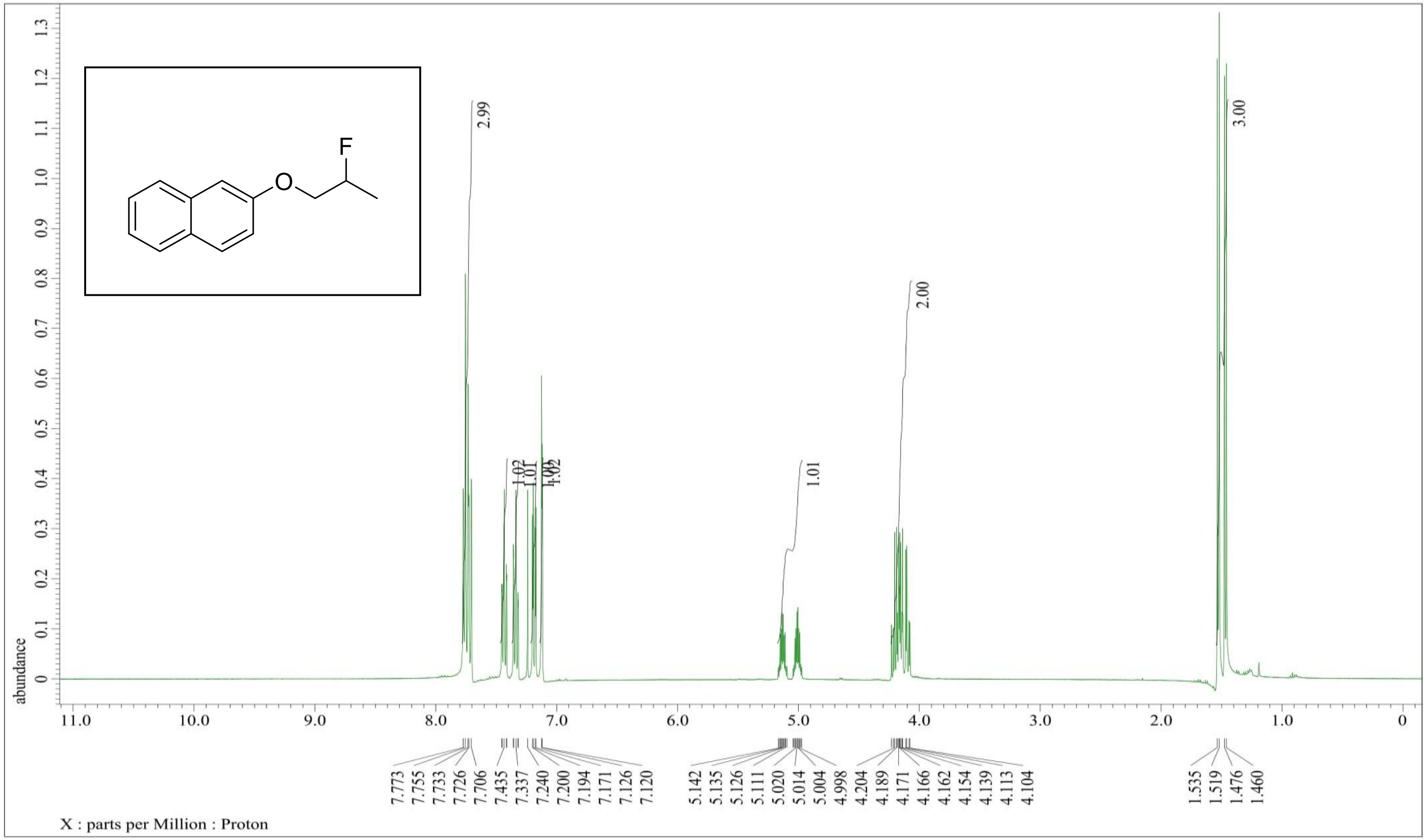

2-(2-fluoropropoxy)naphthalene (5).

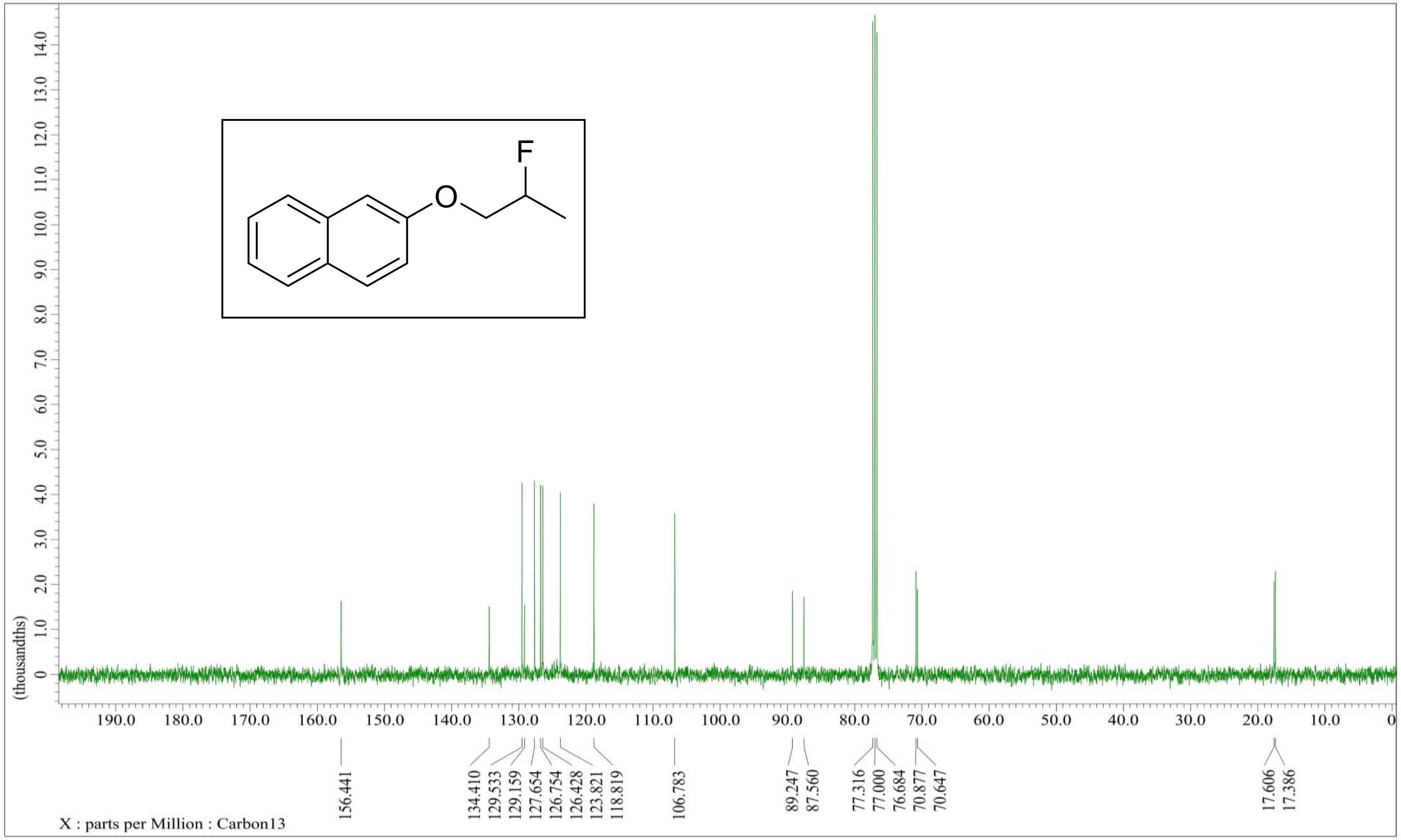

1-(2-Fluoroethyl)naphthalene (6).

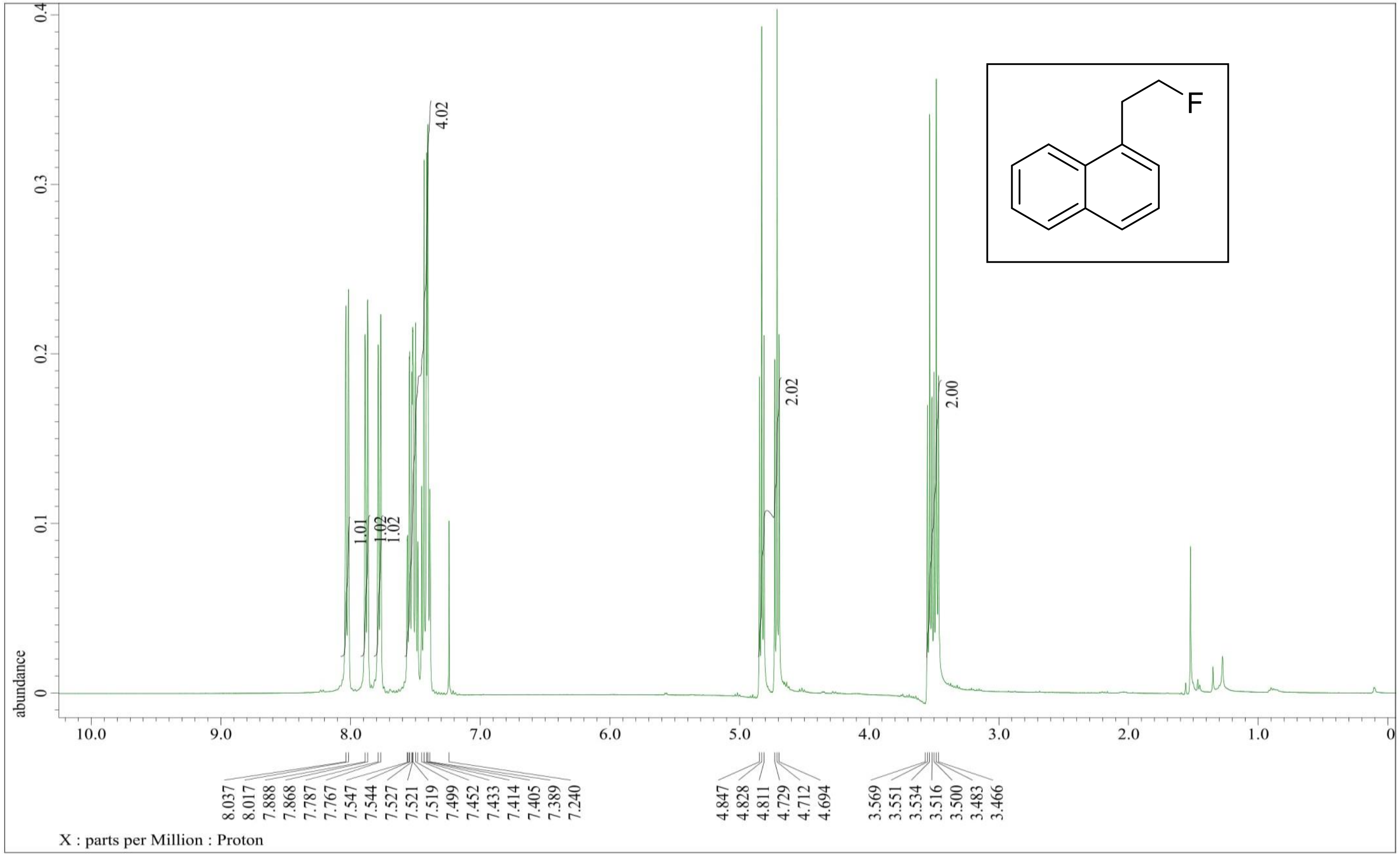

1-(2-Fluoroethyl)naphthalene (6).

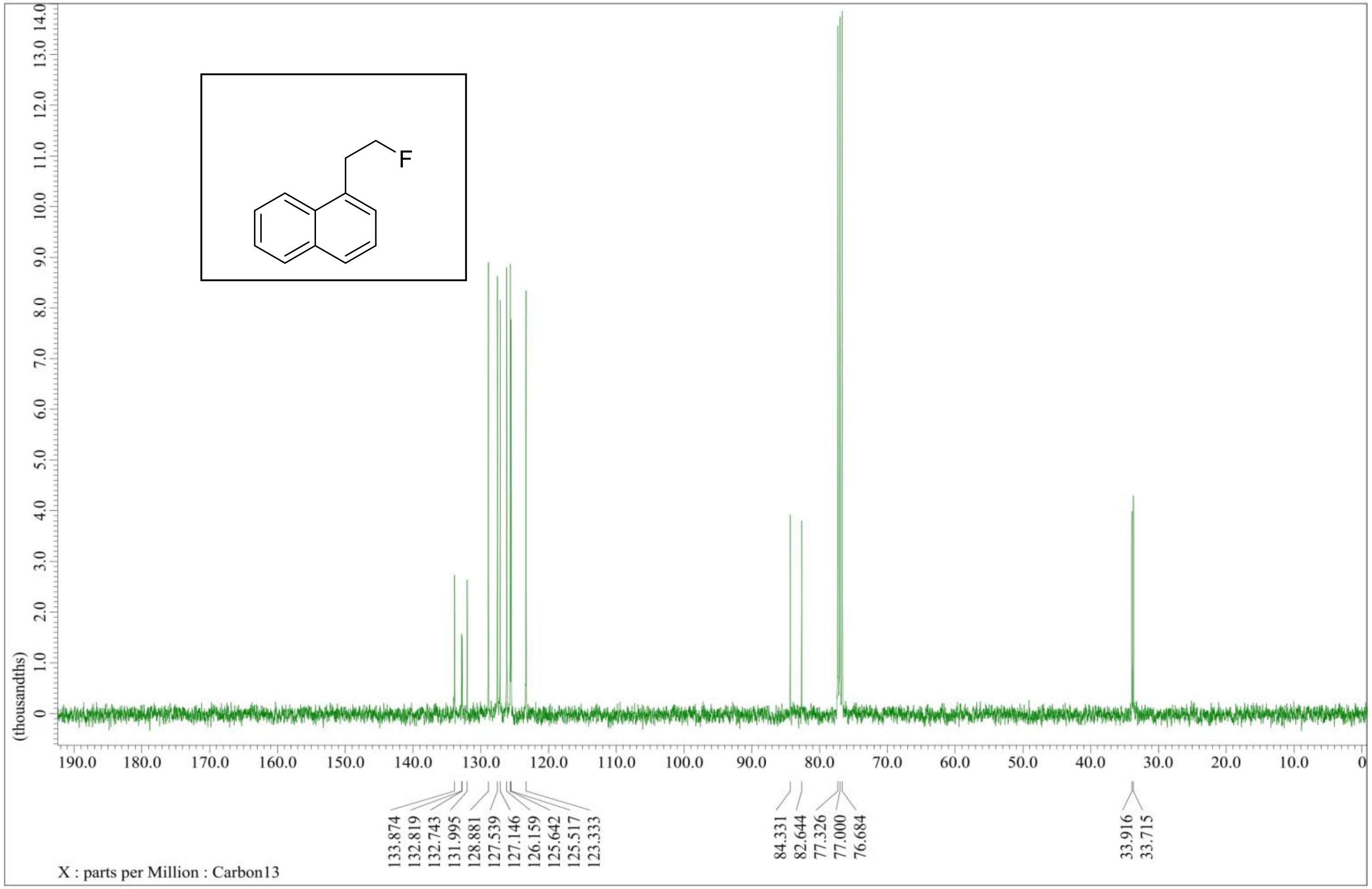

4-(3-fluoropropoxy)-2H-chromen-2-one (7).

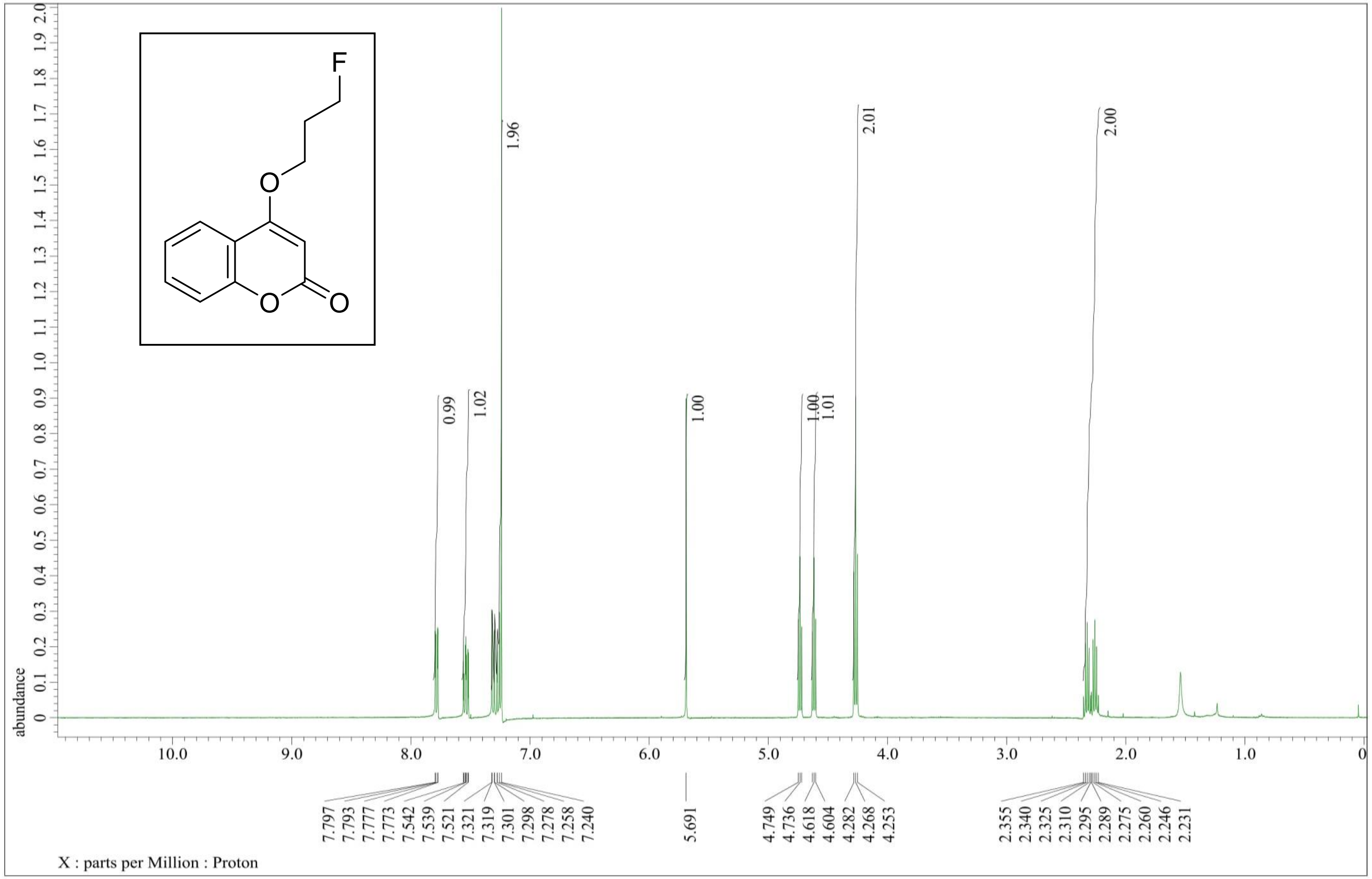

4-(3-fluoropropoxy)-2H-chromen-2-one (7).

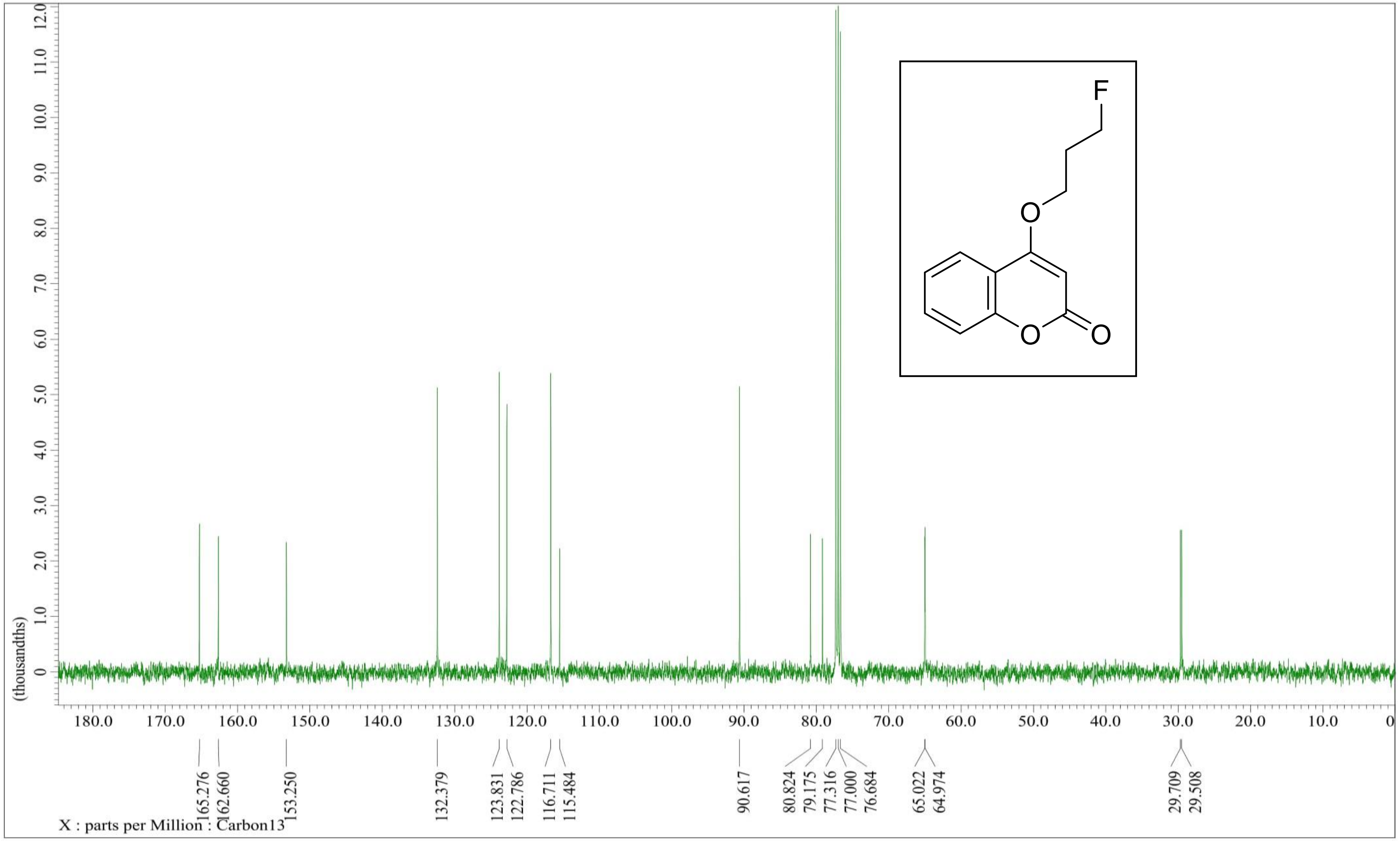

1,17-difluoro-3,6,9,12,15-pentaoxaheptadecane (8).

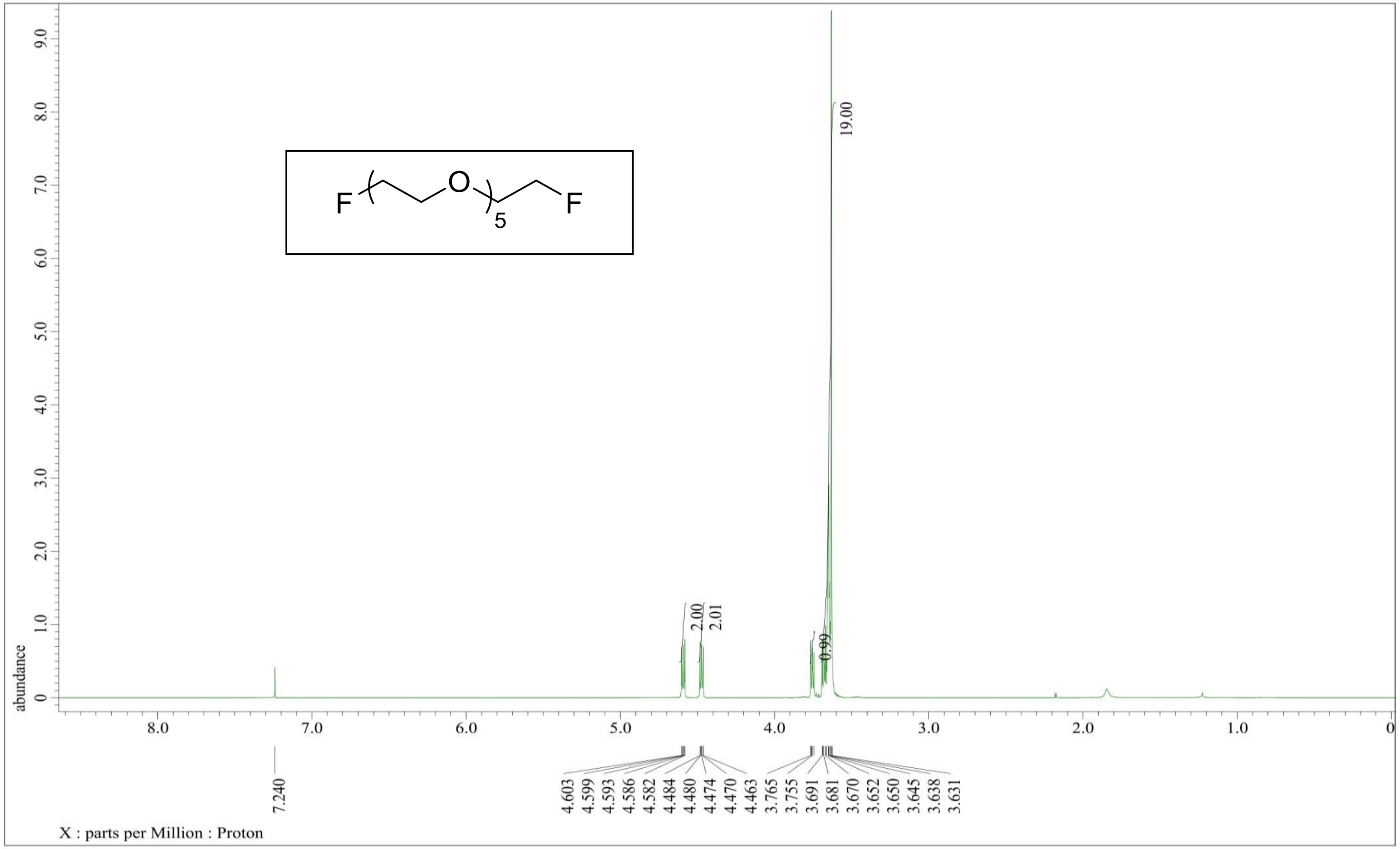

1,17-difluoro-3,6,9,12,15-pentaoxaheptadecane (8).

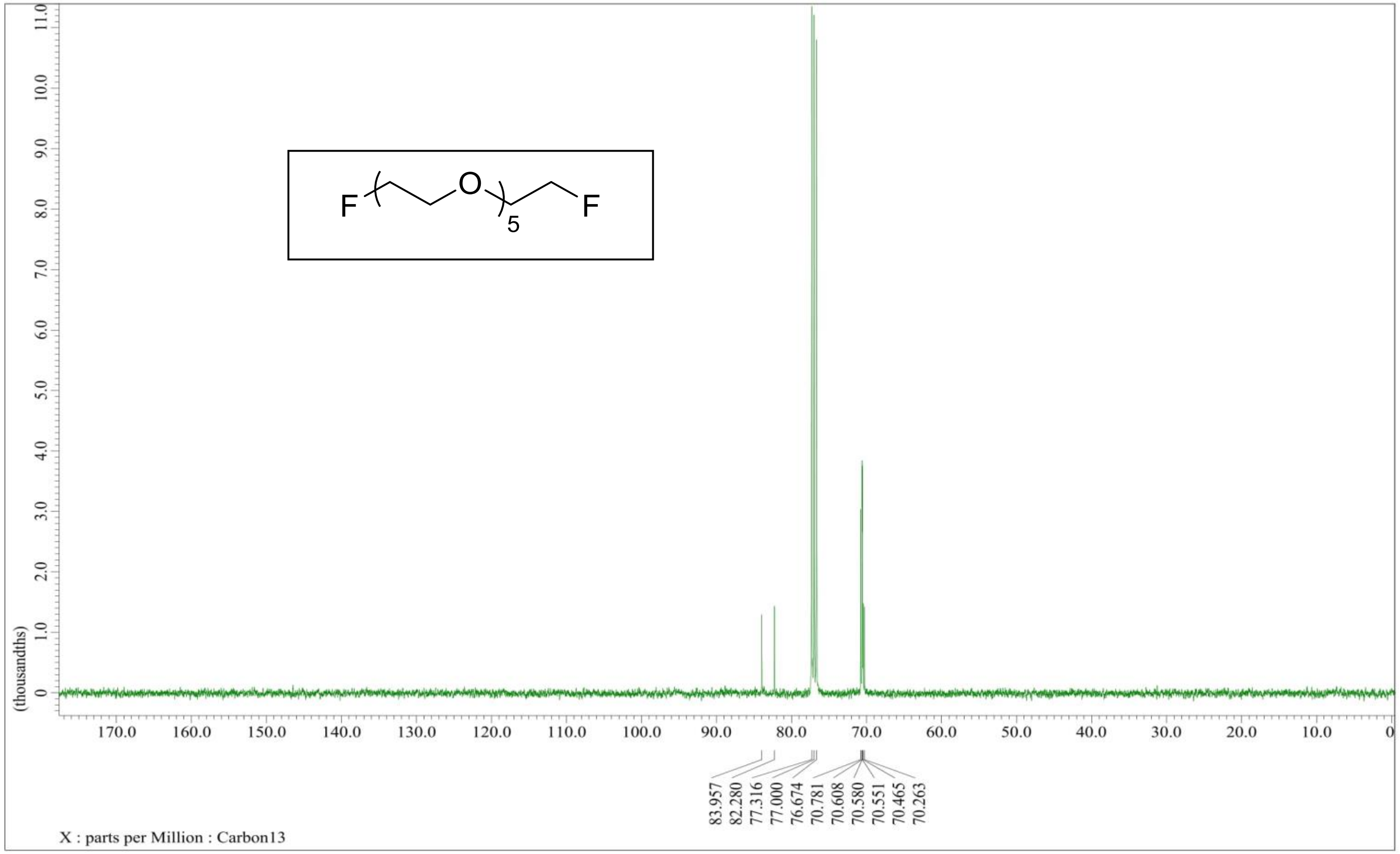

1-(3-fluoropropoxy)-4-nitrobenzene (9).

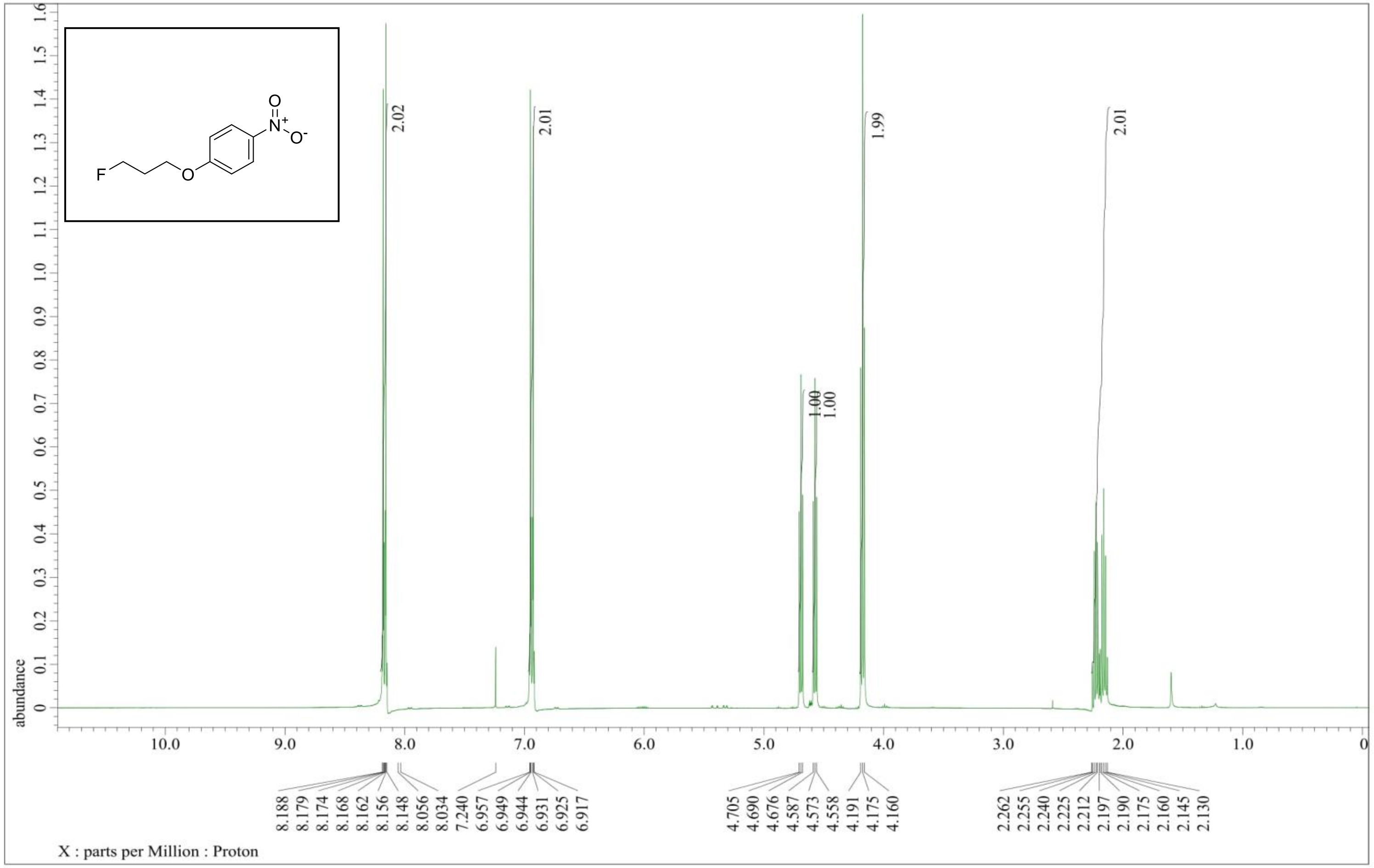

1-(3-fluoropropoxy)-4-nitrobenzene (9).

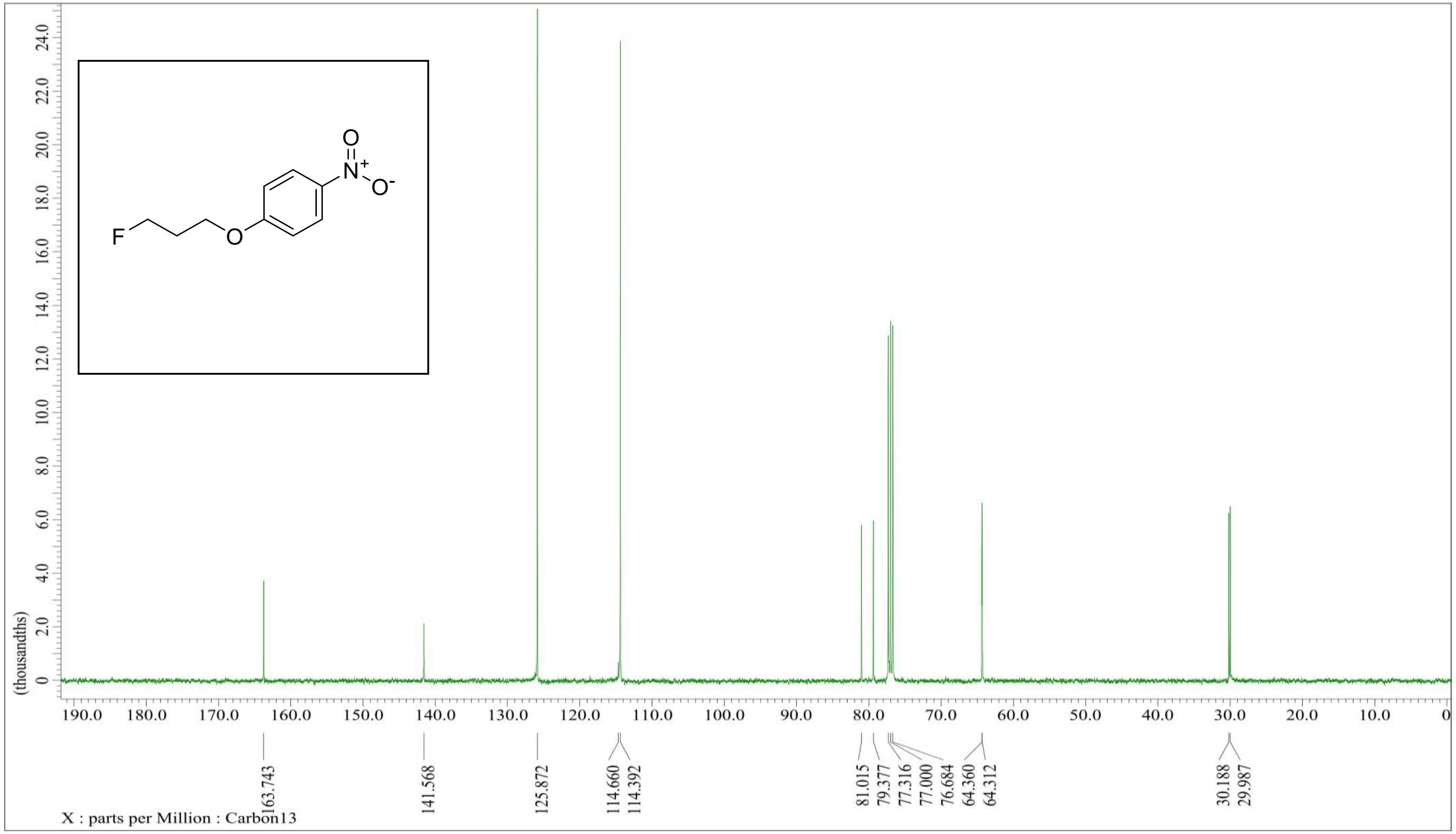

1-(3-Fluoropropyl)-4-nitroimidazole (10).

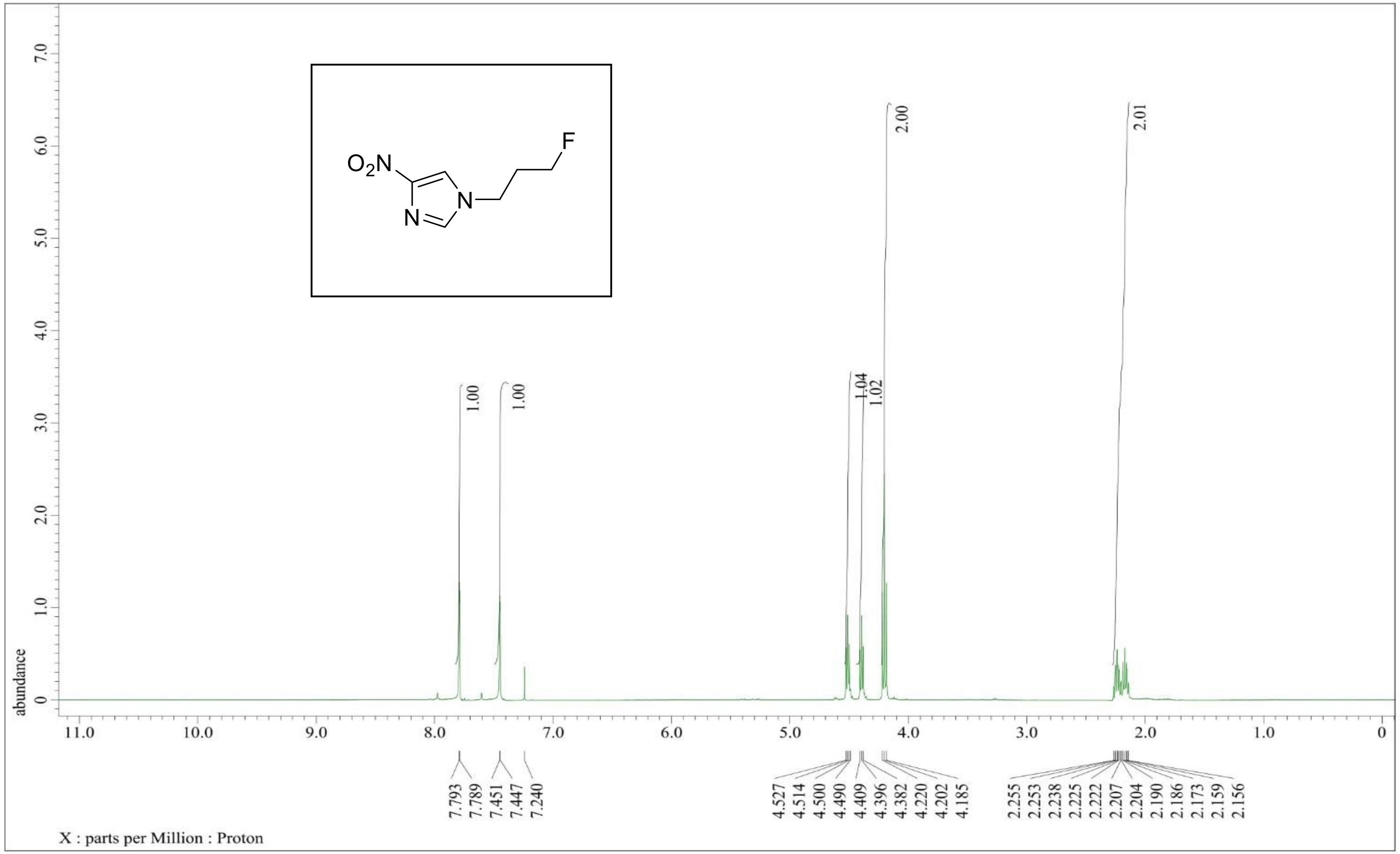

1-(3-Fluoropropyl)-4-nitroimidazole (10).

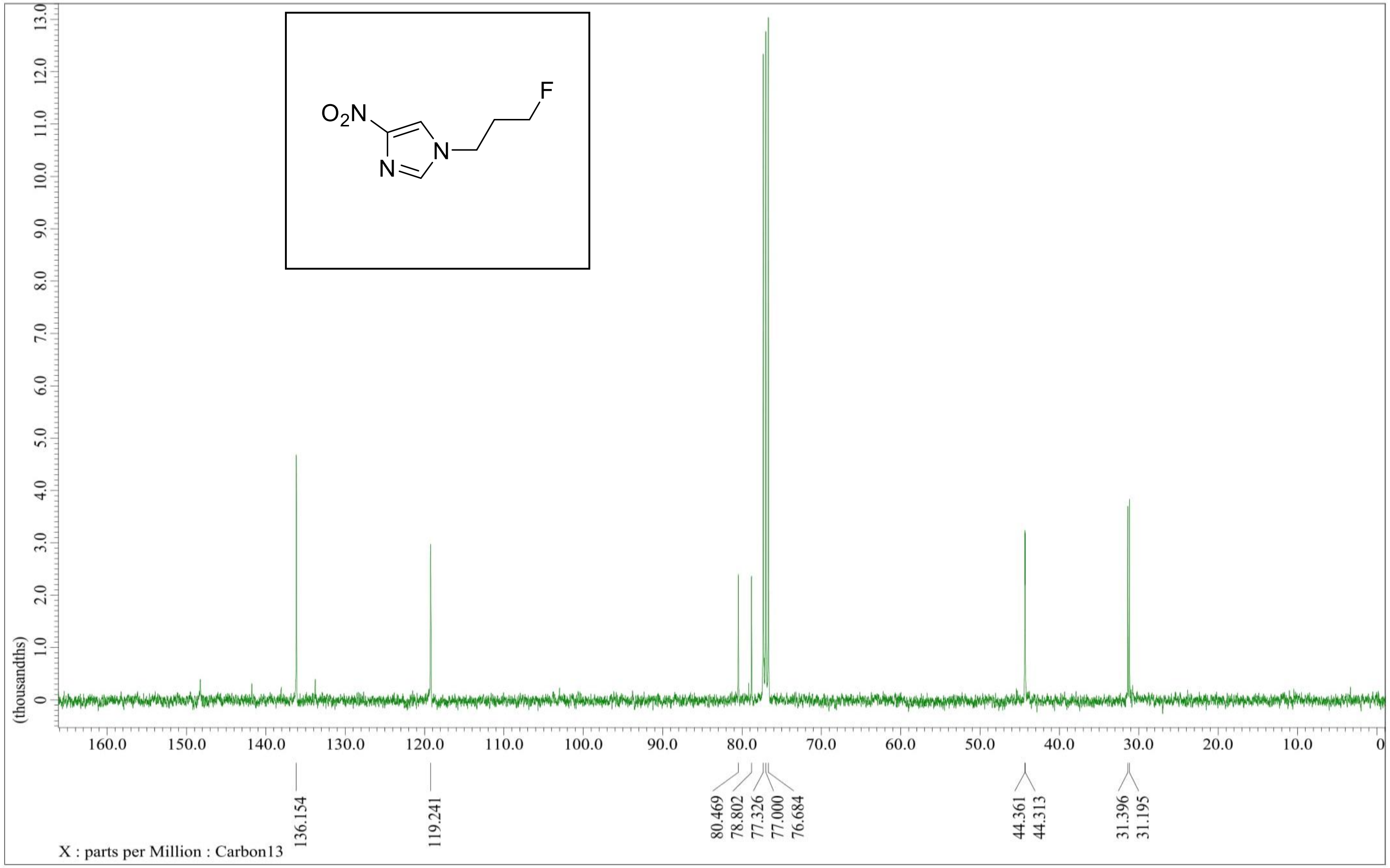

1,2:3,4-Di-*O*-isopropylidene-6-fluoro-6-deoxy-α-D-galactopyranose (11).

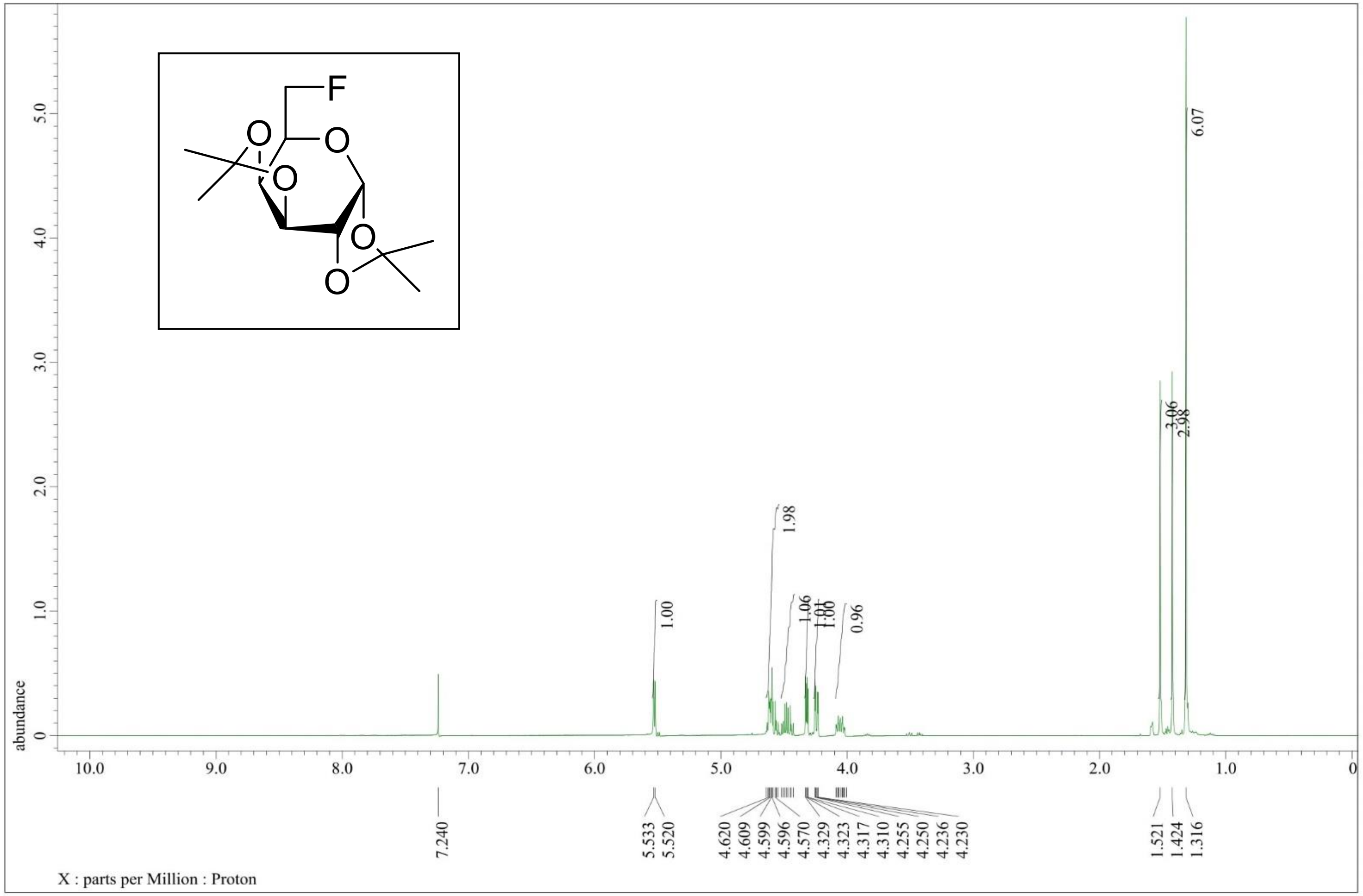

1,2:3,4-Di-*O*-isopropylidene-6-fluoro-6-deoxy-α-D-galactopyranose (11).

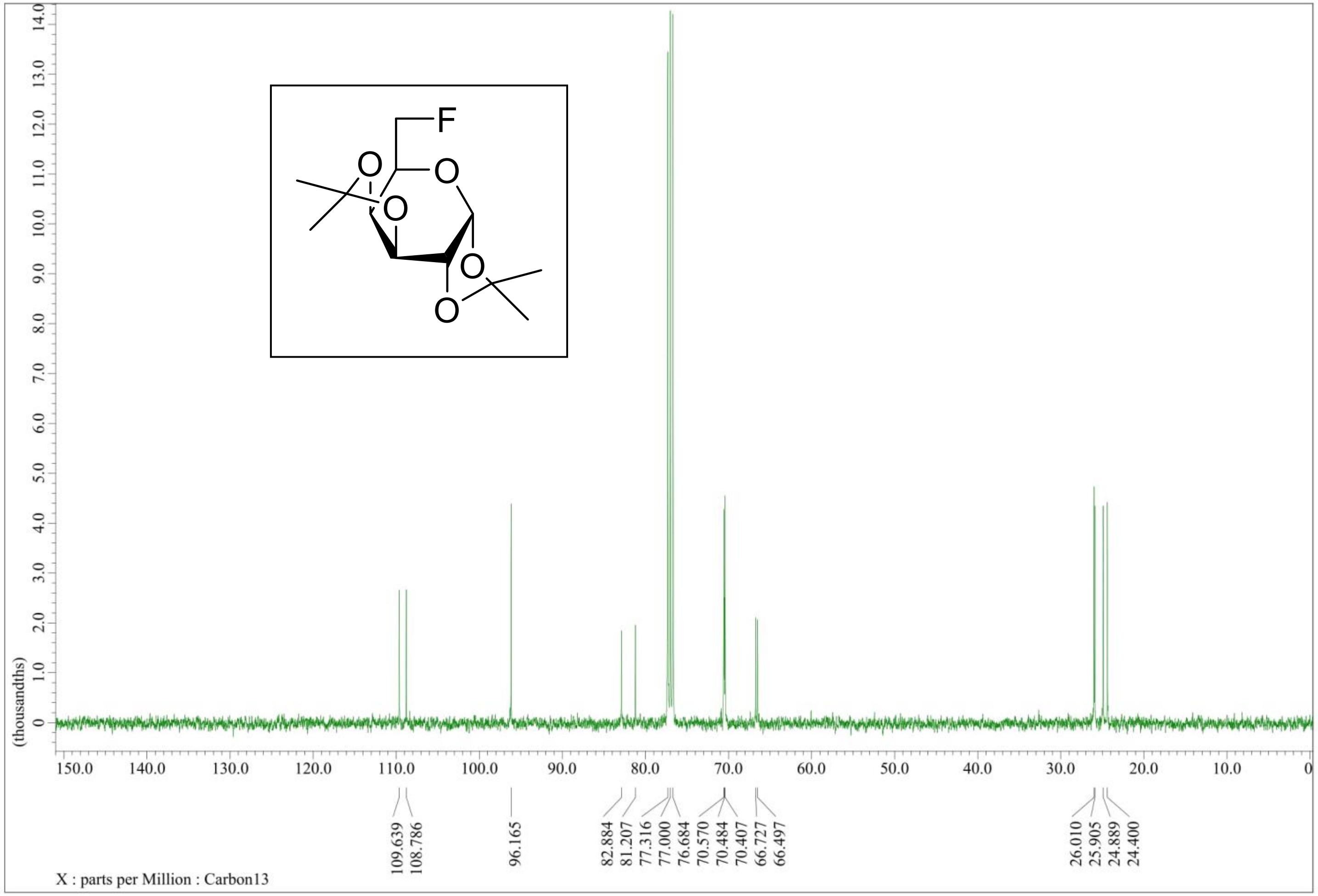

3-Fluoro-picoline *N*-Oxide (14).

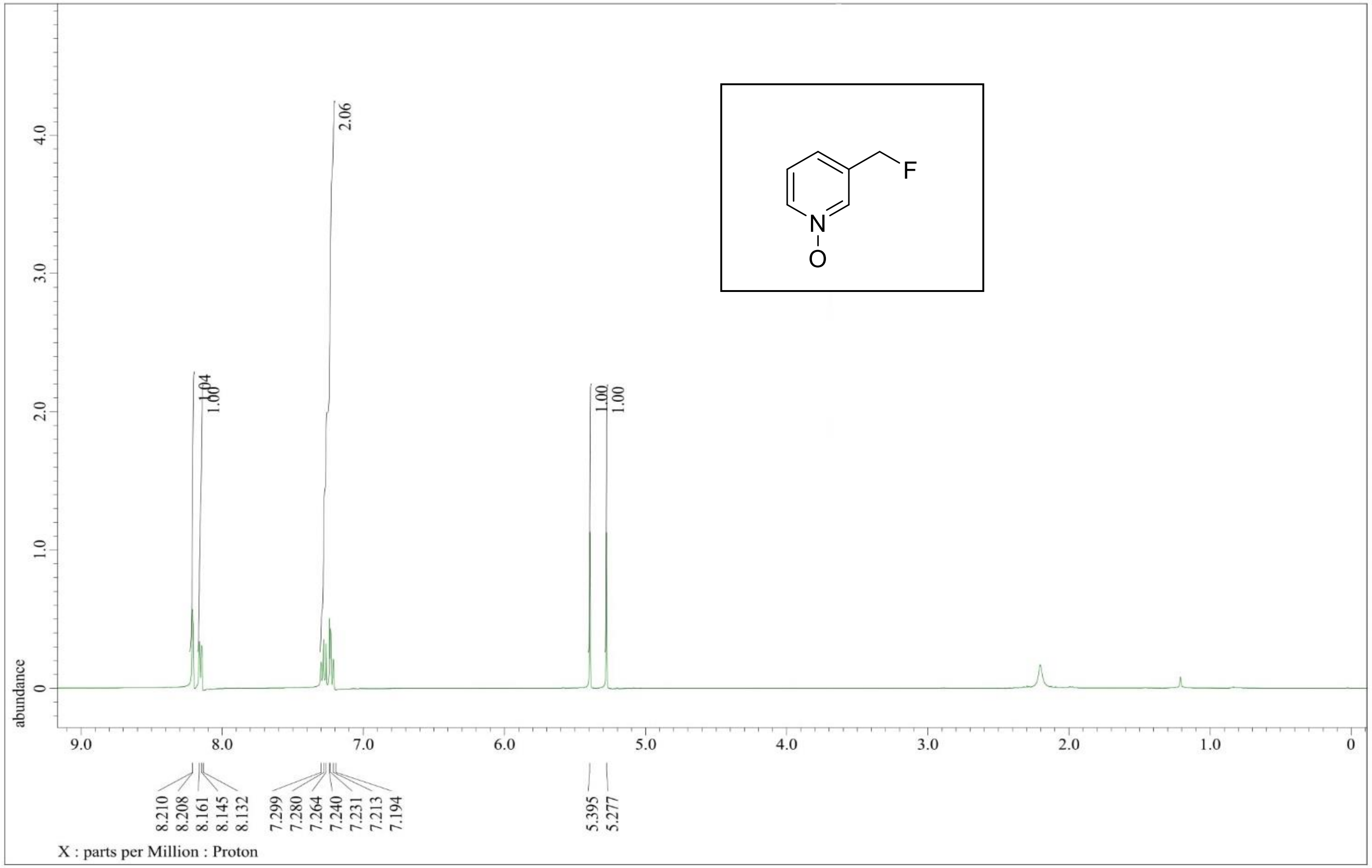

3-Fluoro-picoline *N*-Oxide (14).

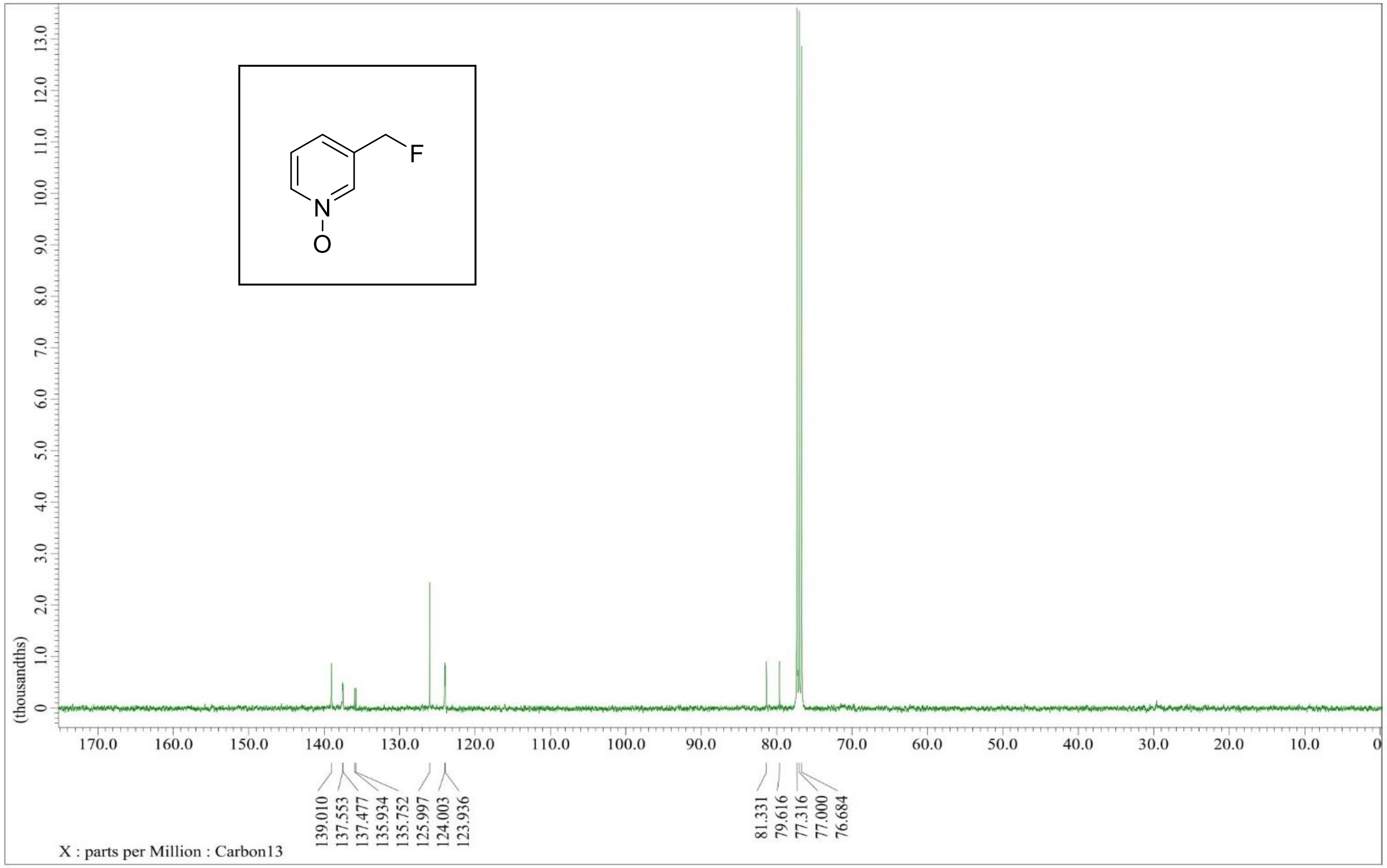

2-Fluoro-2'-acetonaphthone (**15**).

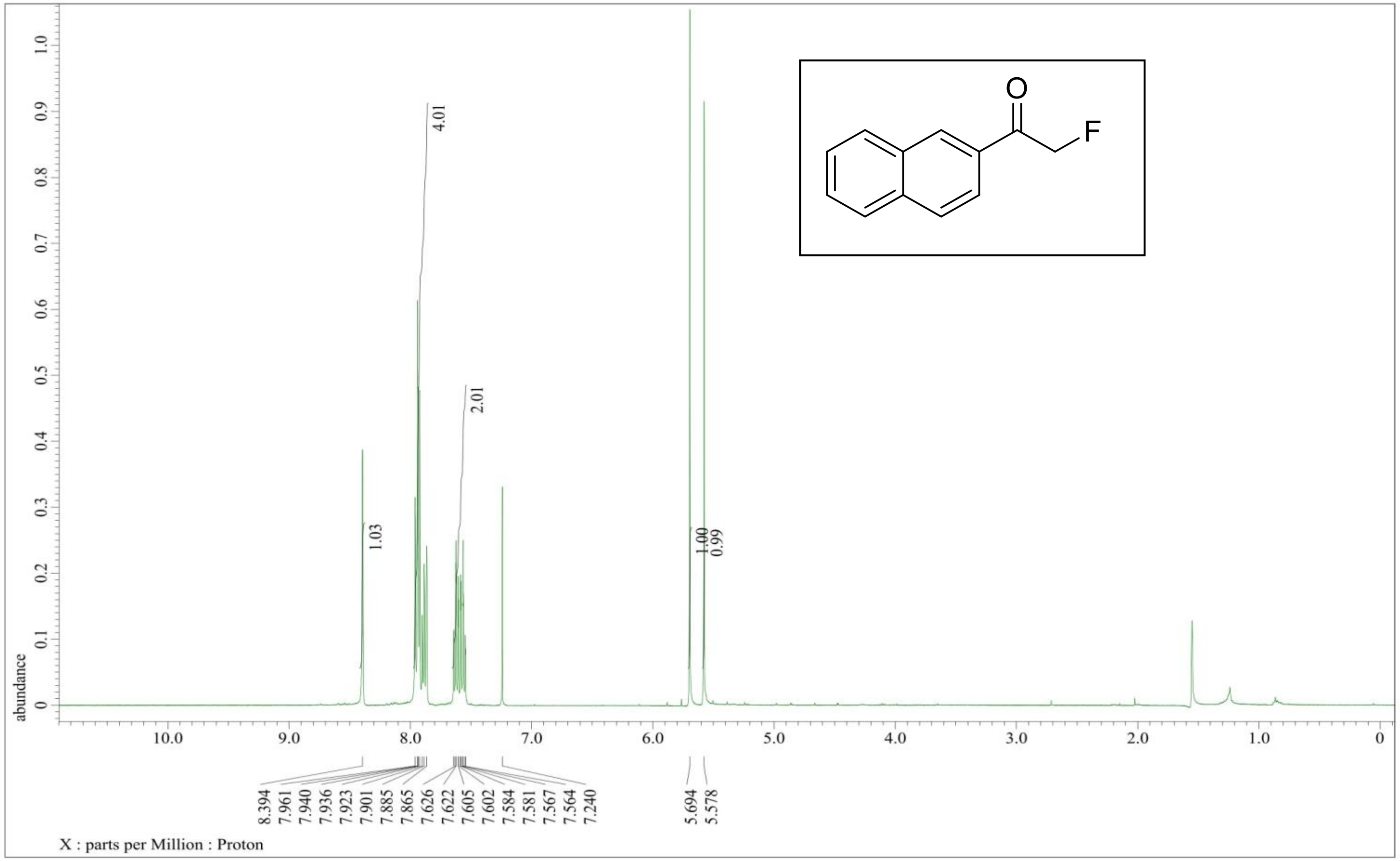

2-Fluoro-2'-acetonaphthone (15).

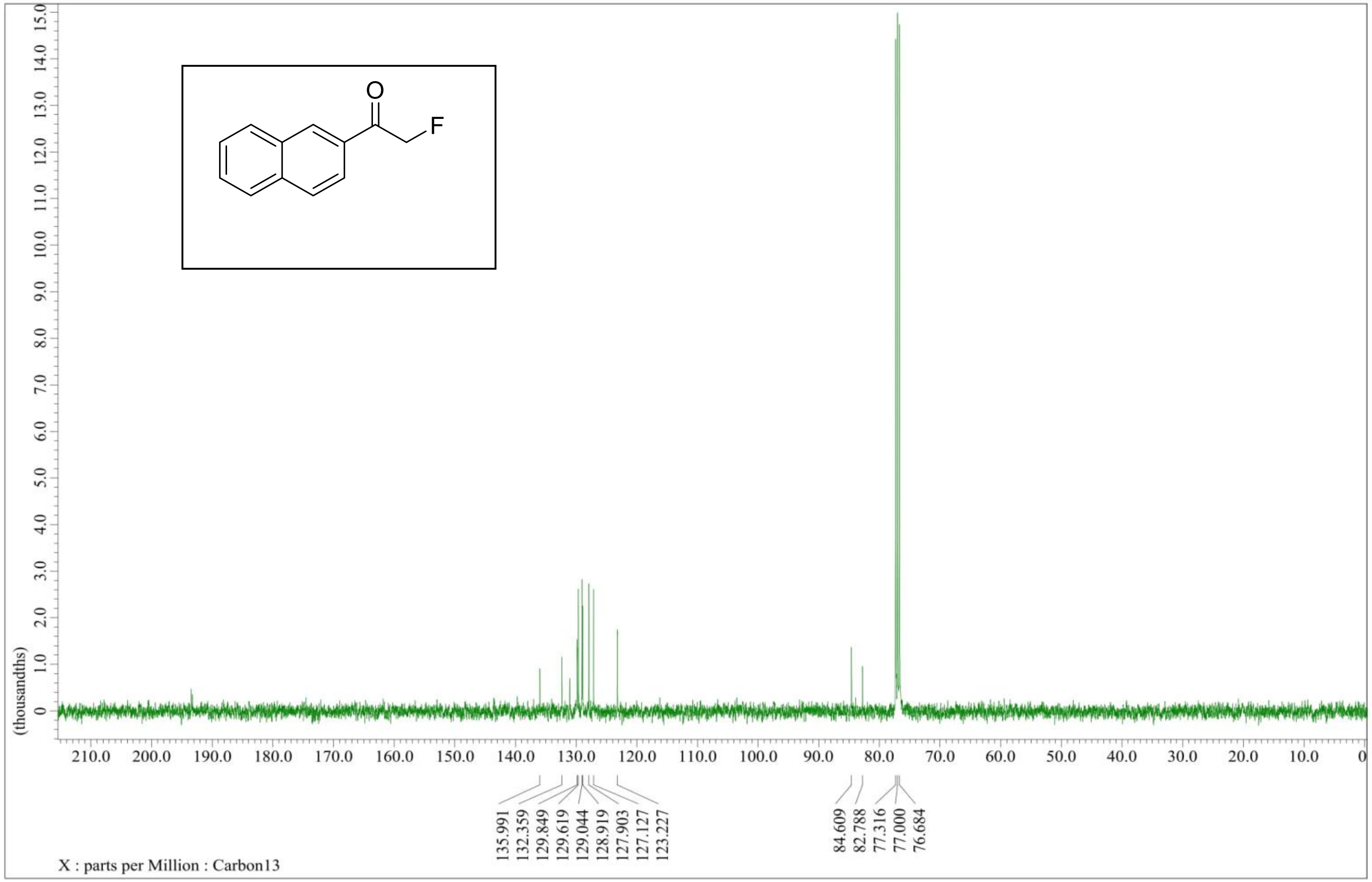

3-*O*-(3-Fluoropropyl)estrone (16).

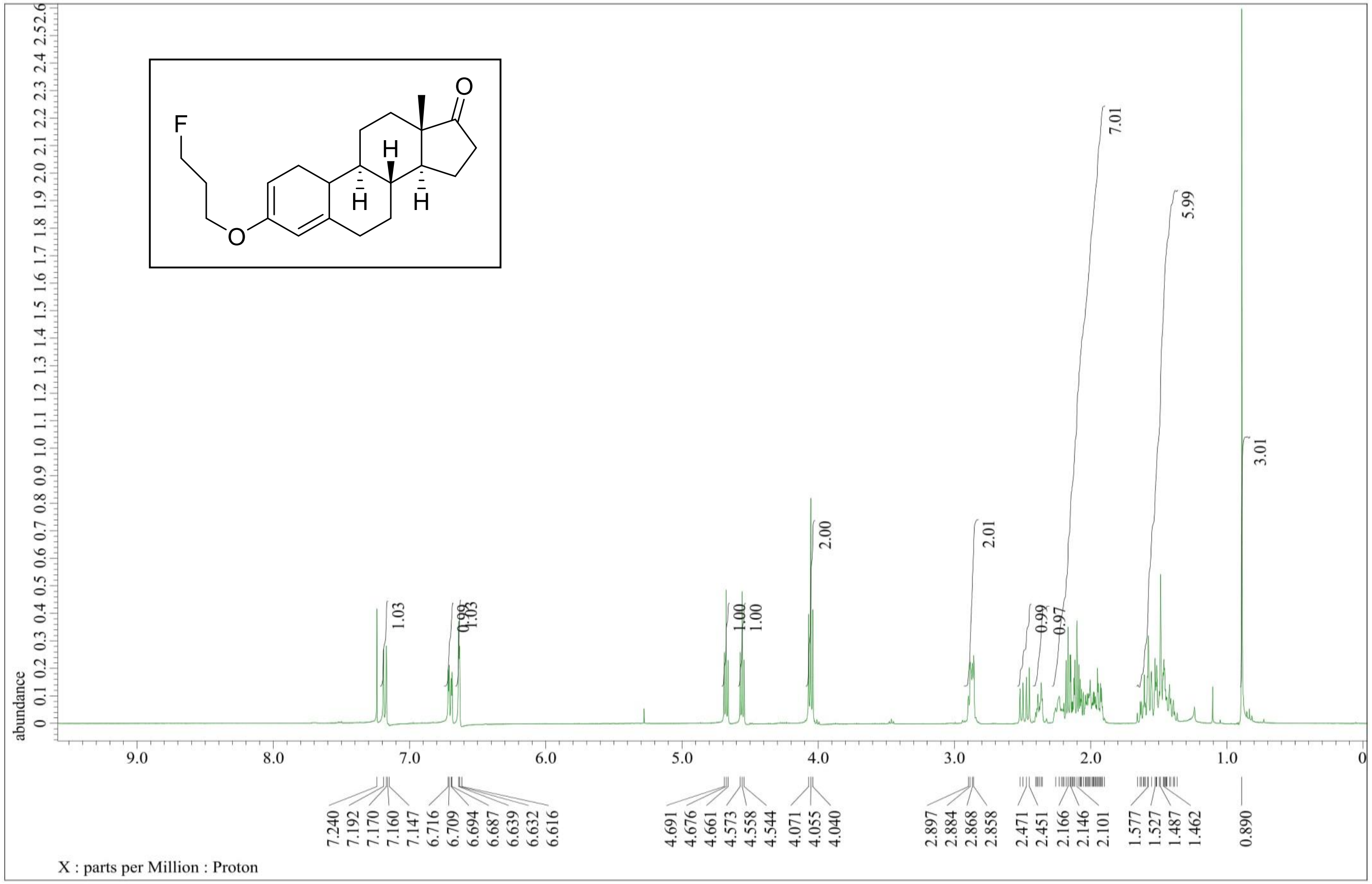

3-*O*-(3-Fluoropropyl)estrone (16).

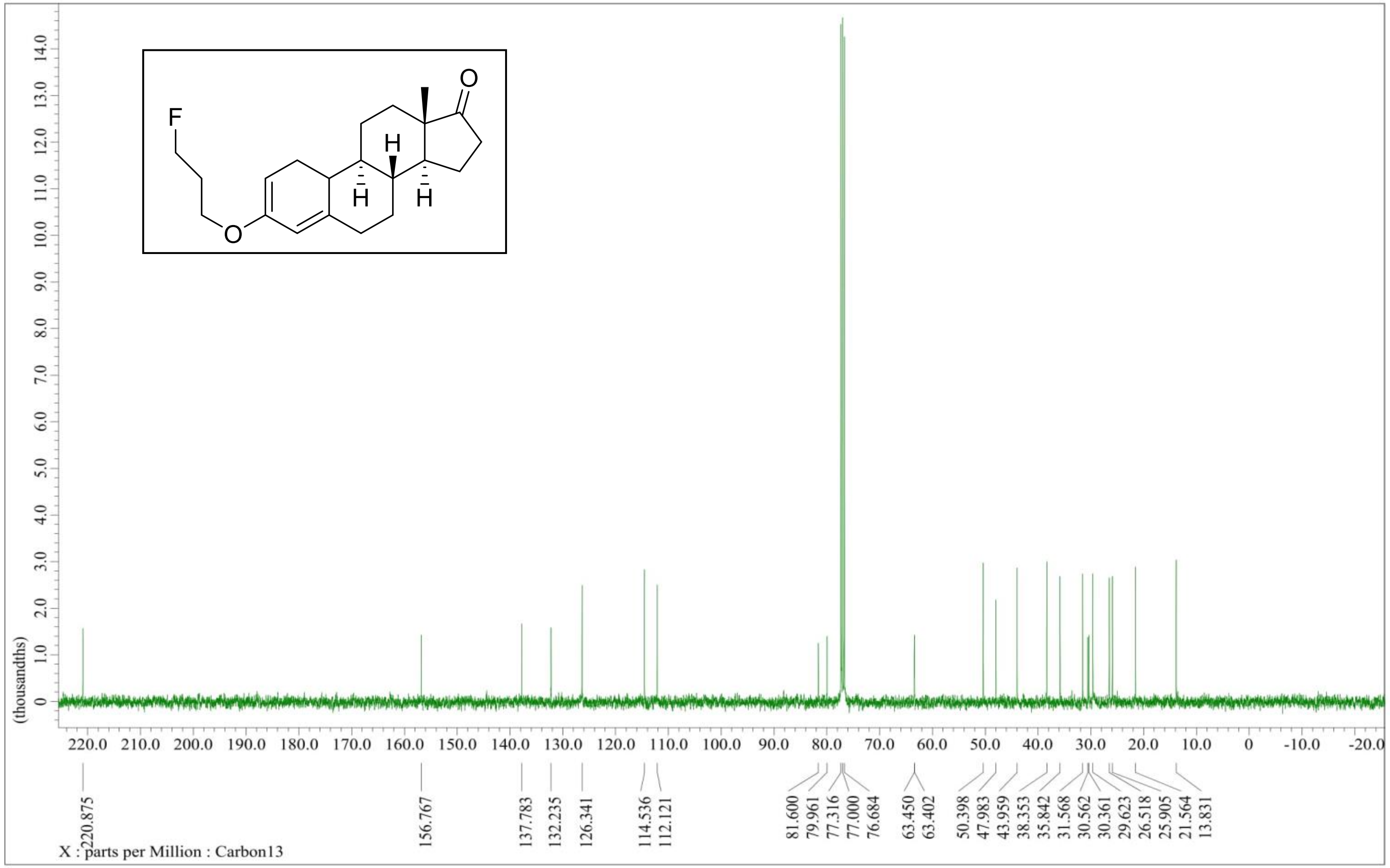

^{1}H and ^{13}C NMR spectra of substrates.

2-(3-Methanesulfonyloxypropoxy)naphthalene (3).

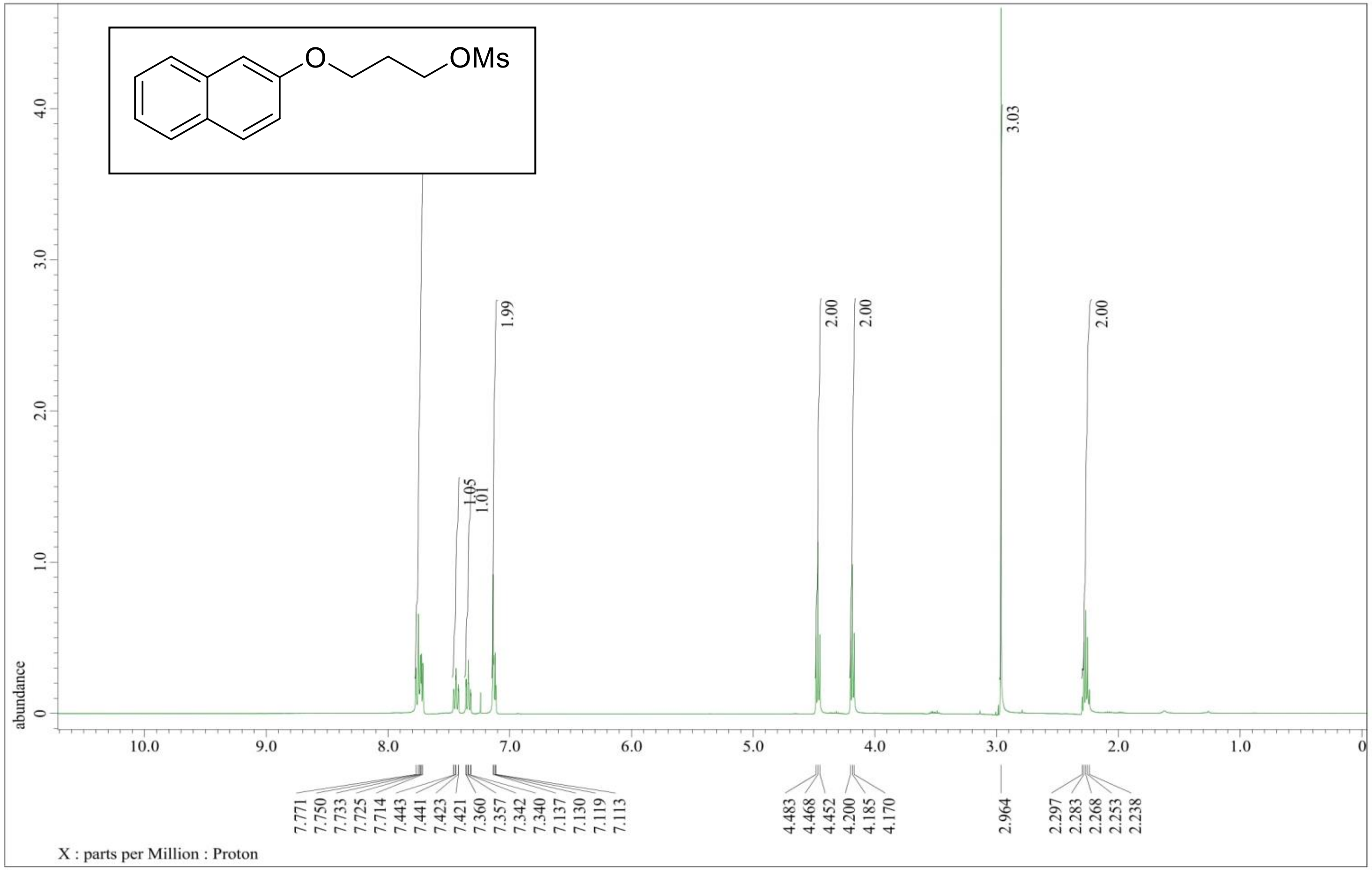

2-(3-Methanesulfonyloxypropoxy)naphthalene (3).

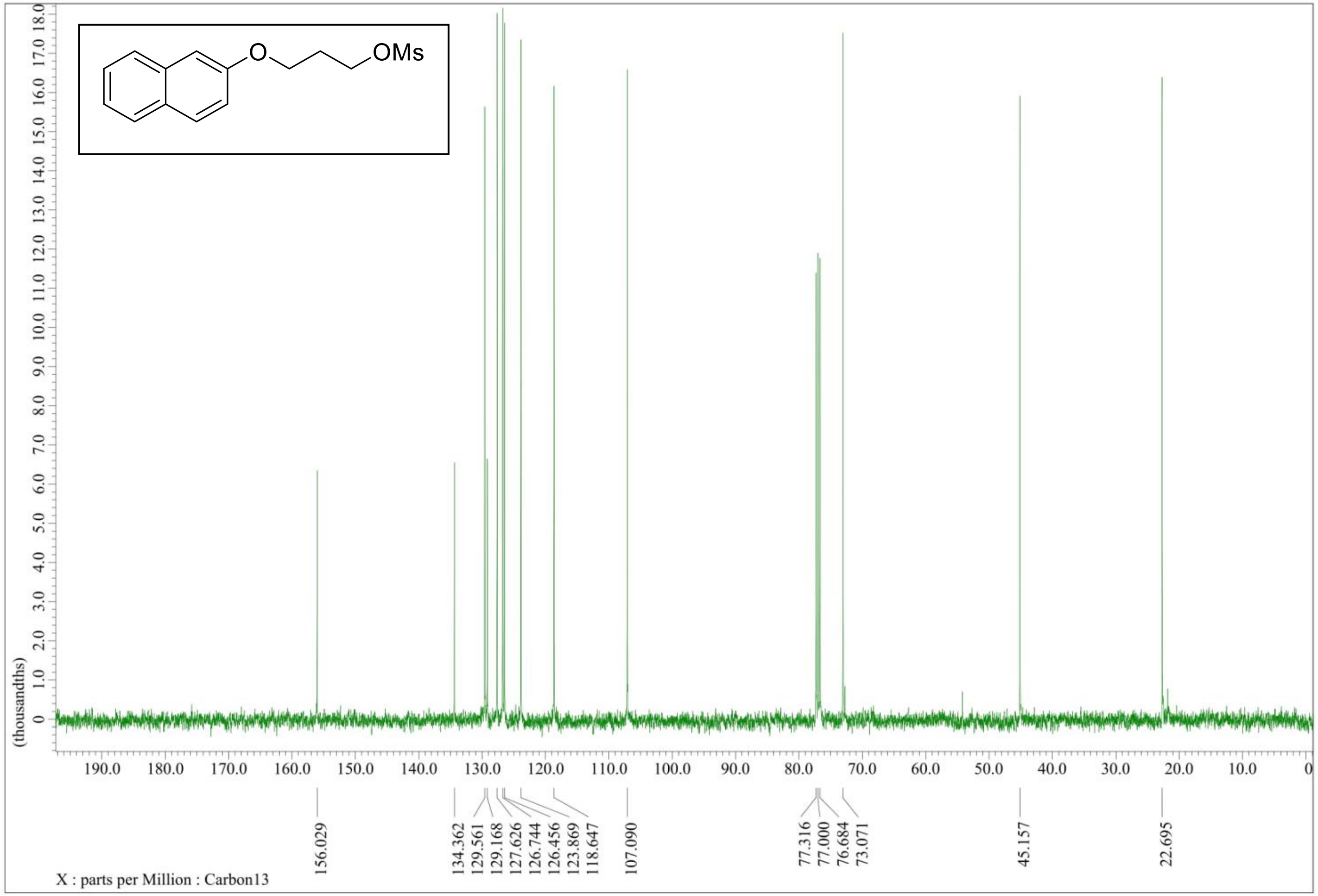

2-(2-Bromopropoxy)naphthalene (entry 1 and 2 in Table 2)

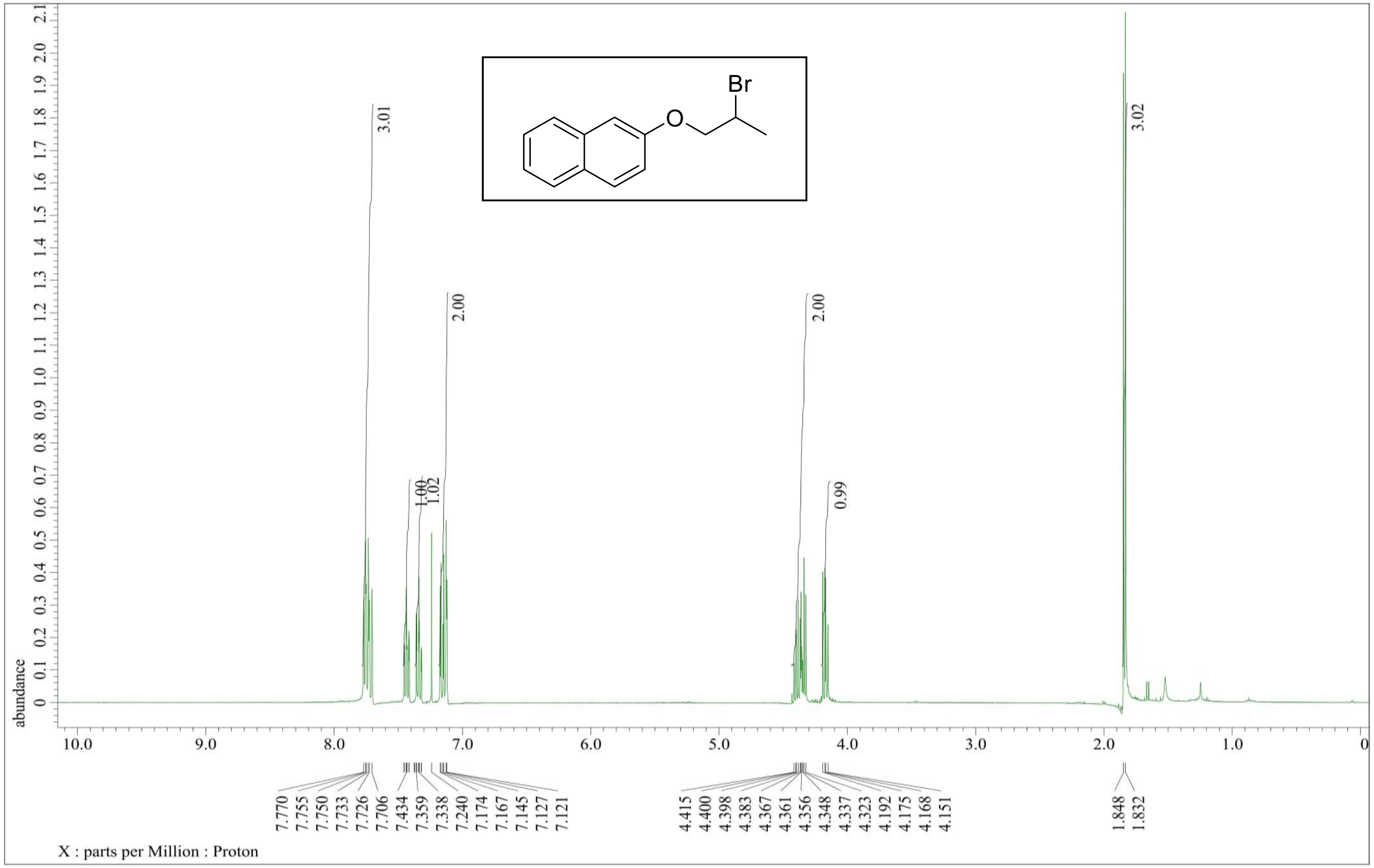

2-(2-Bromopropoxy)naphthalene (entry 1 and 2 in Table 2)

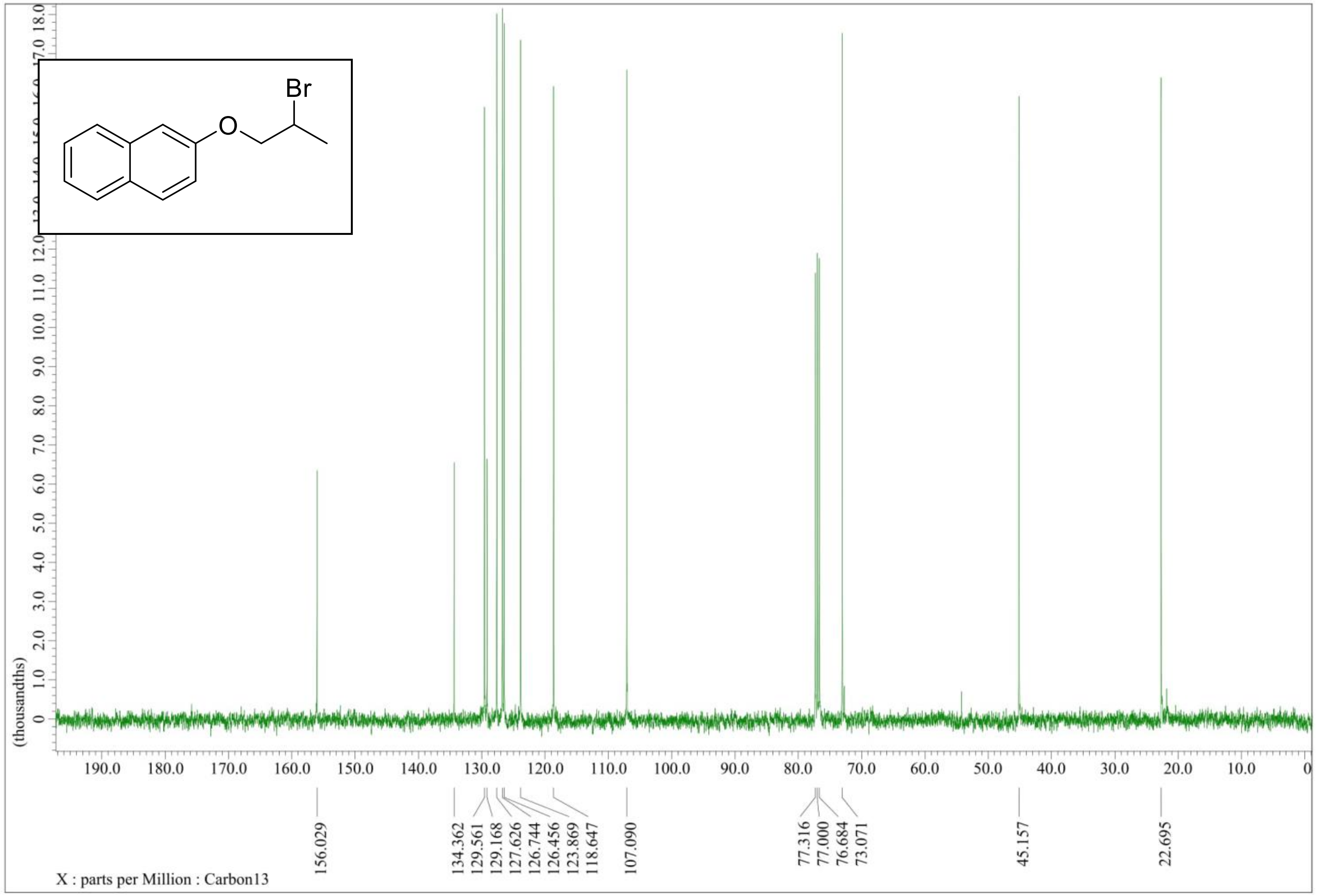

1-(naphthalen-2-yloxy)propan-2-yl methanesulfonate (entry 2 and 3 in Table 2)

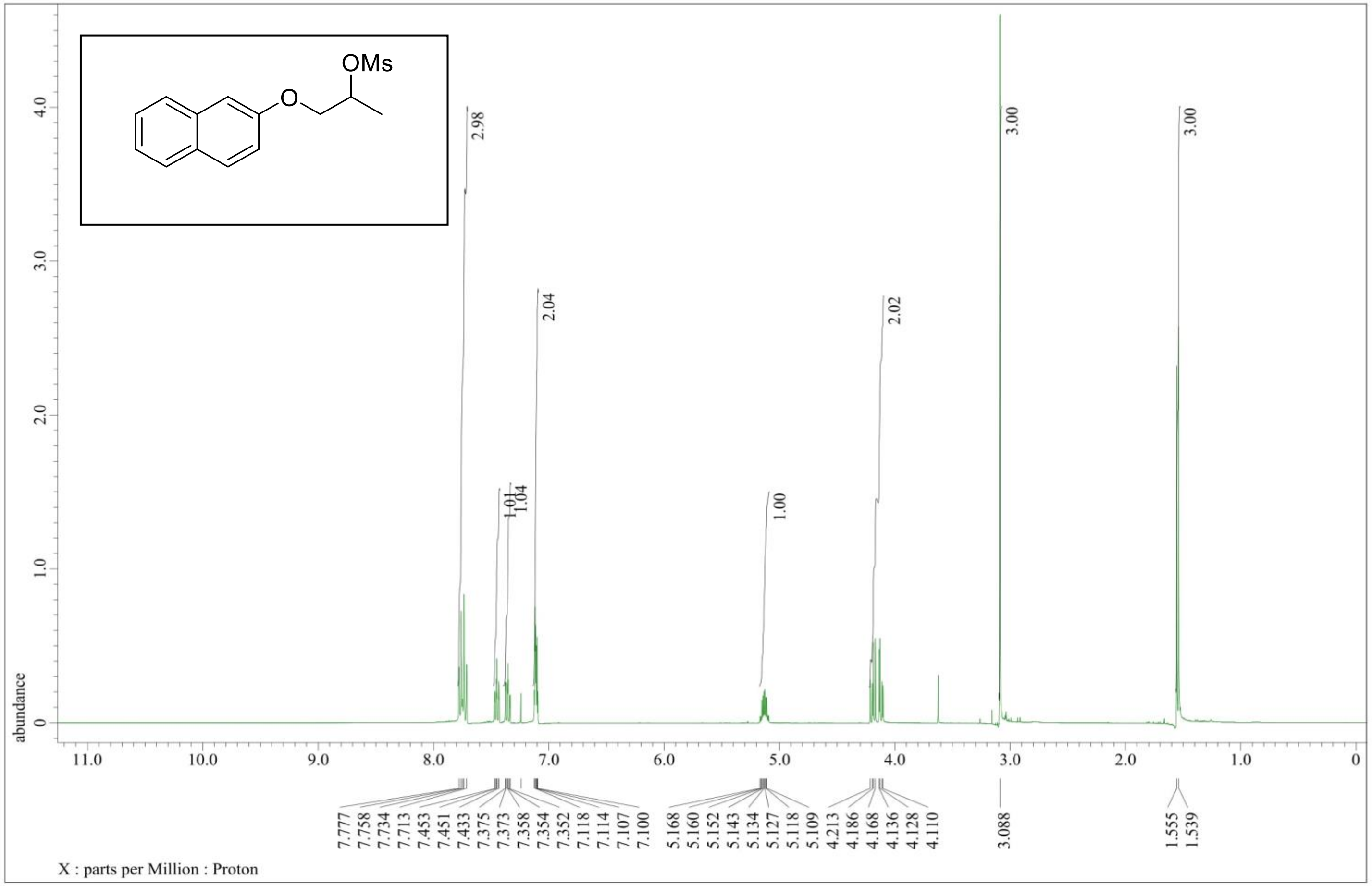

1-(naphthalen-2-yloxy)propan-2-yl methanesulfonate (entry 2 and 3 in Table 2)

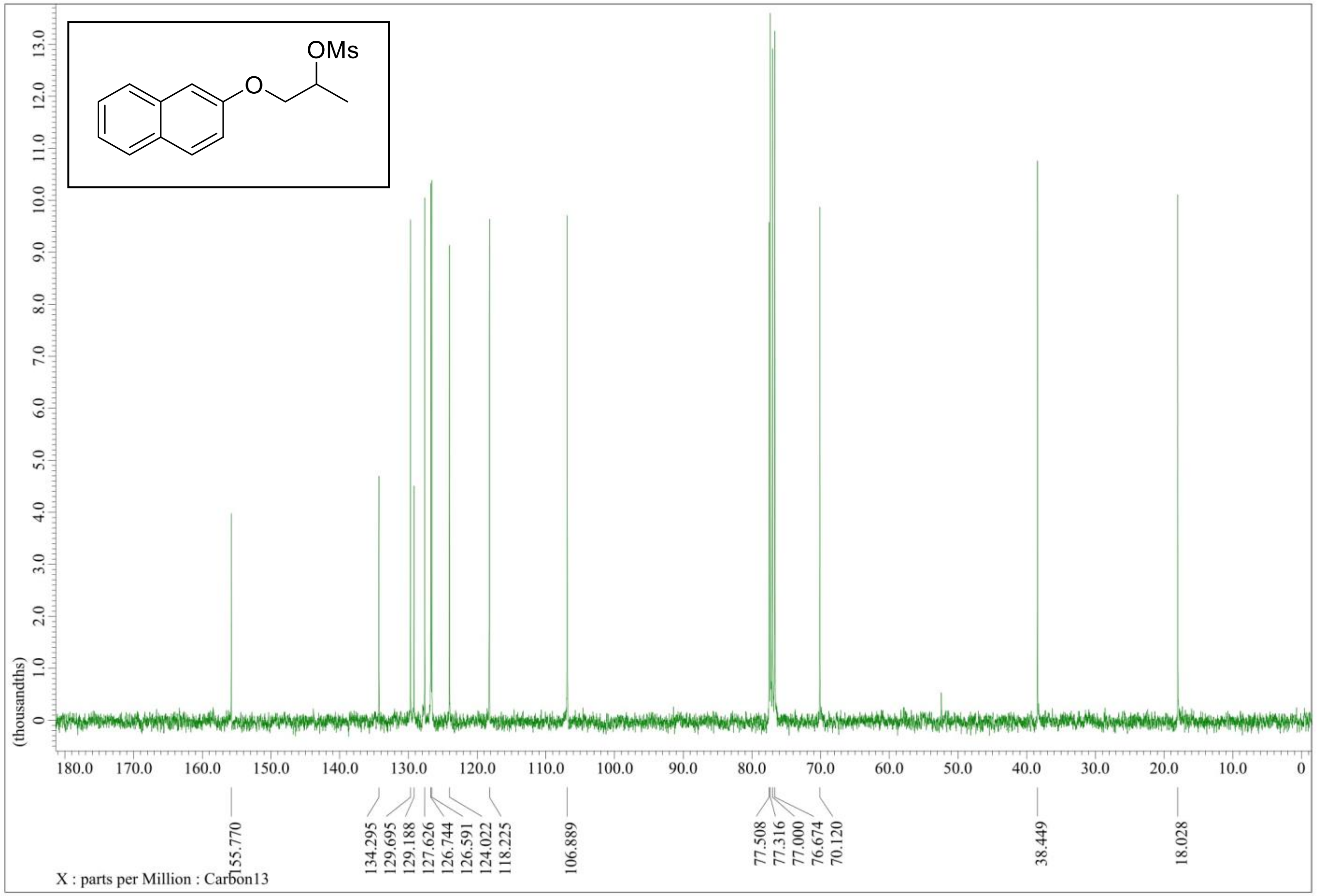

1-(2-Methanesulfonylethyl)naphthalene (entry 5 and 6 in Table 2)

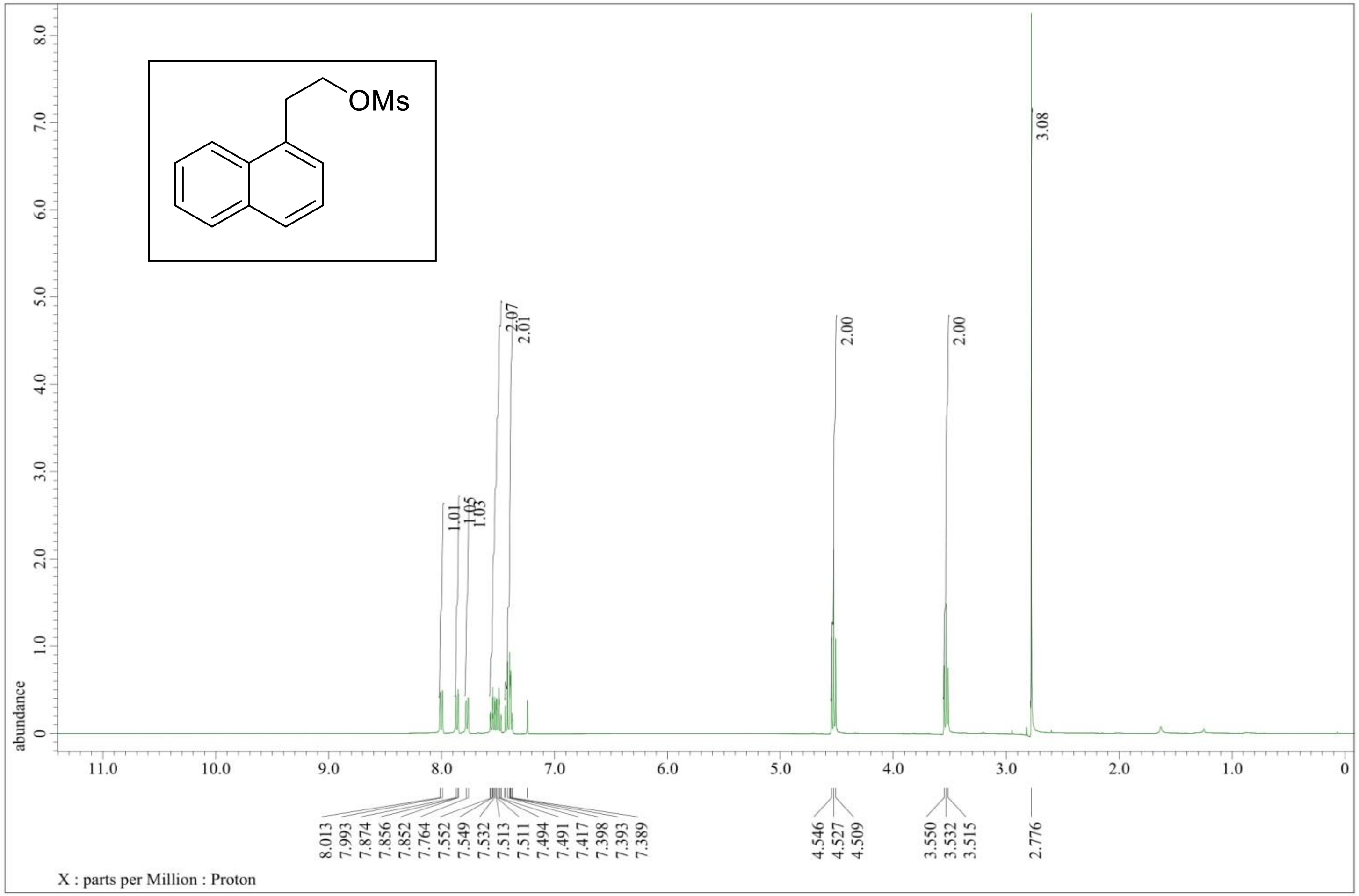

1-(2-Methanesulfonylethyl)naphthalene (entry 5 and 6 in Table 2)

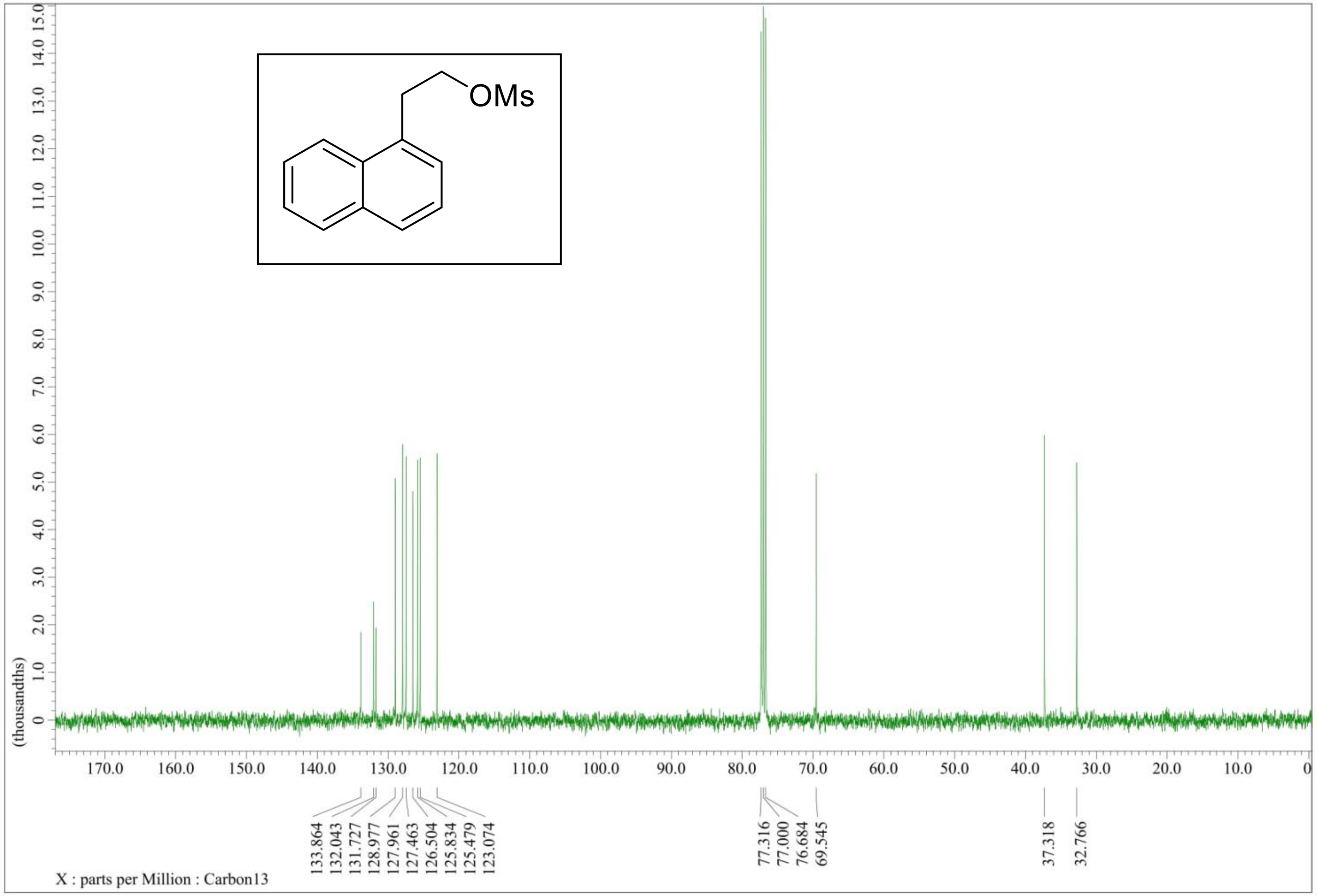

1,17-dibromo-3,6,9,12,15-pentaoxaheptadecane (entry 10 in Table 2)

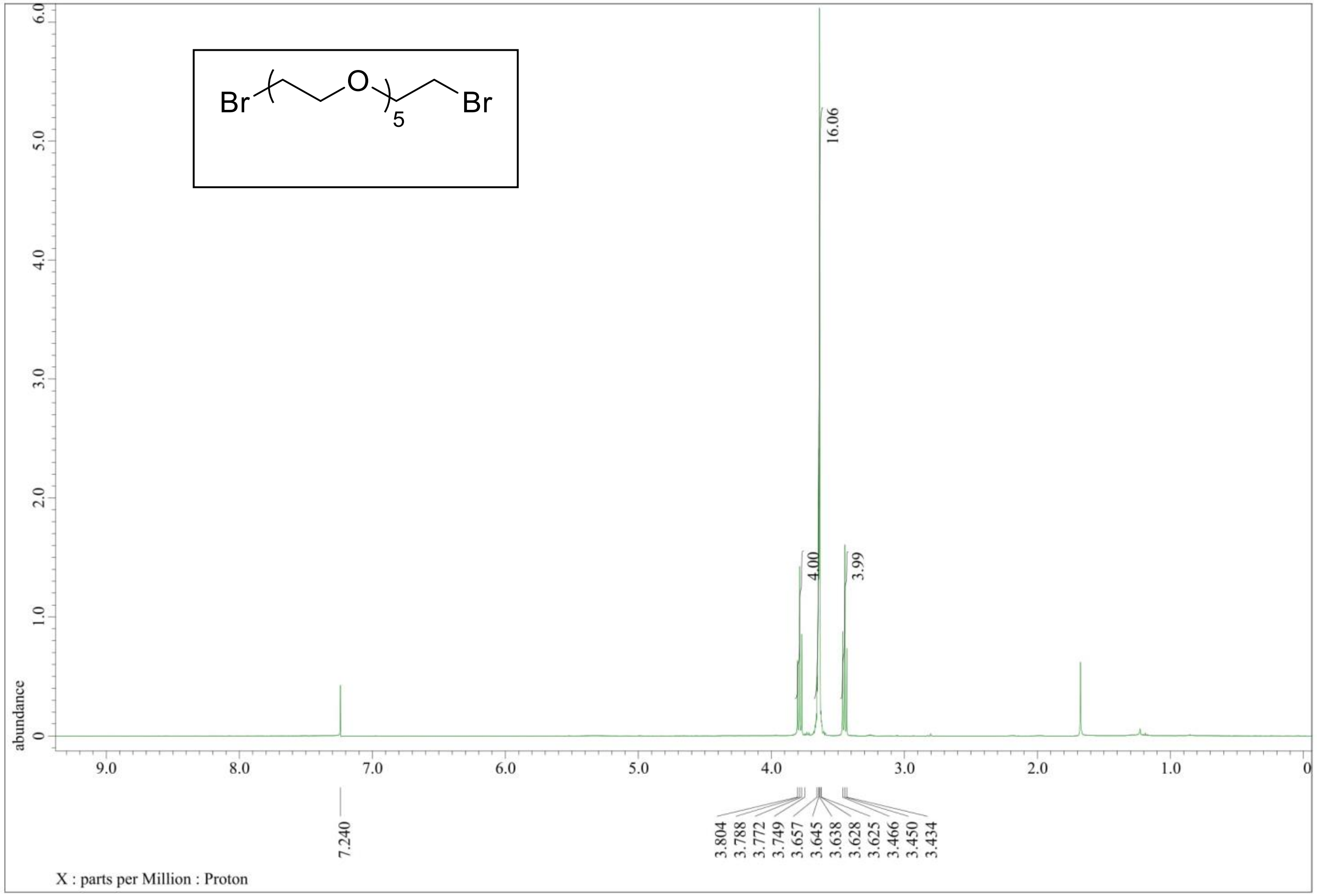

1,17-dibromo-3,6,9,12,15-pentaoxaheptadecane (entry 10 in Table 2)

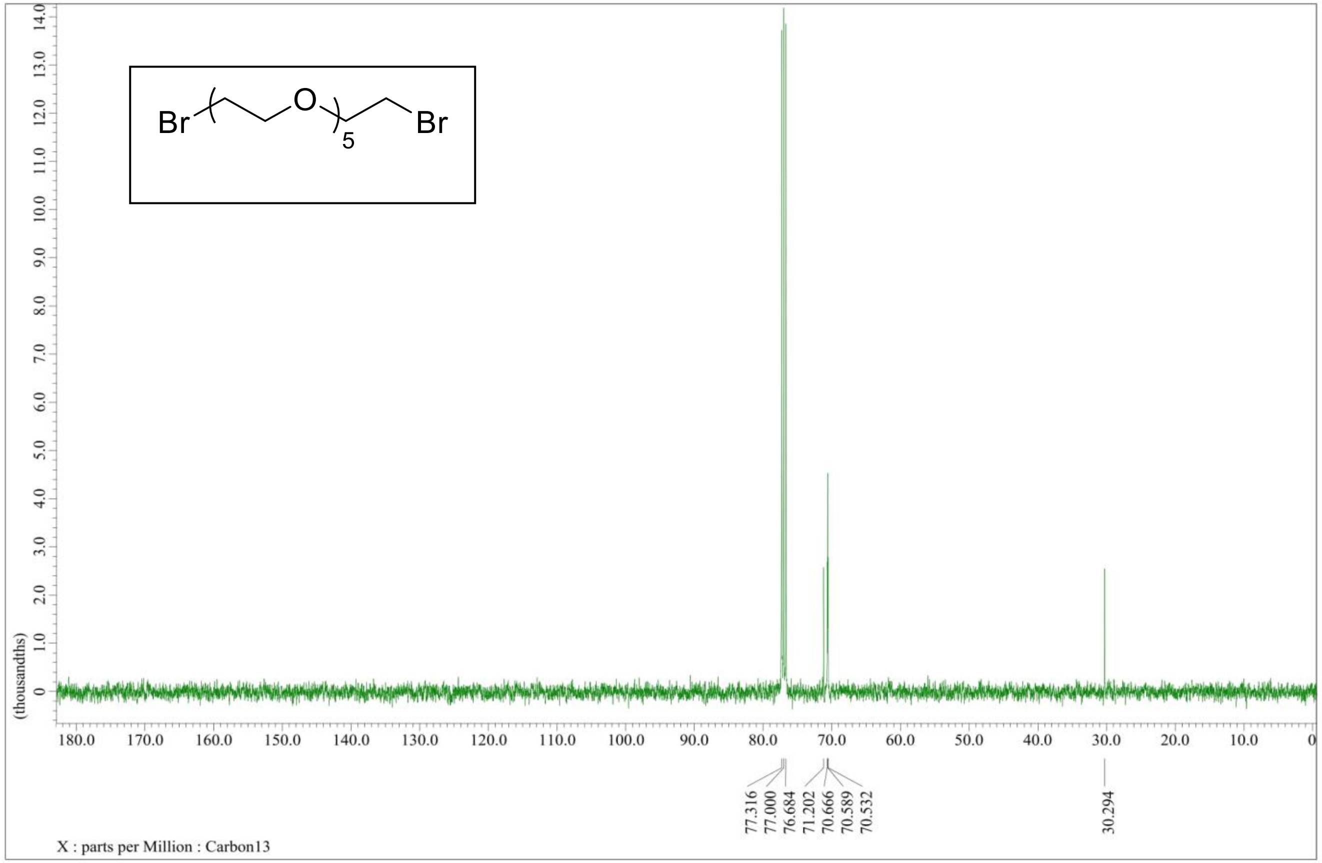

3-((2-oxo-2H-chromen-4-yl)oxy)propyl methanesulfonate (entry 7 in Table 2)

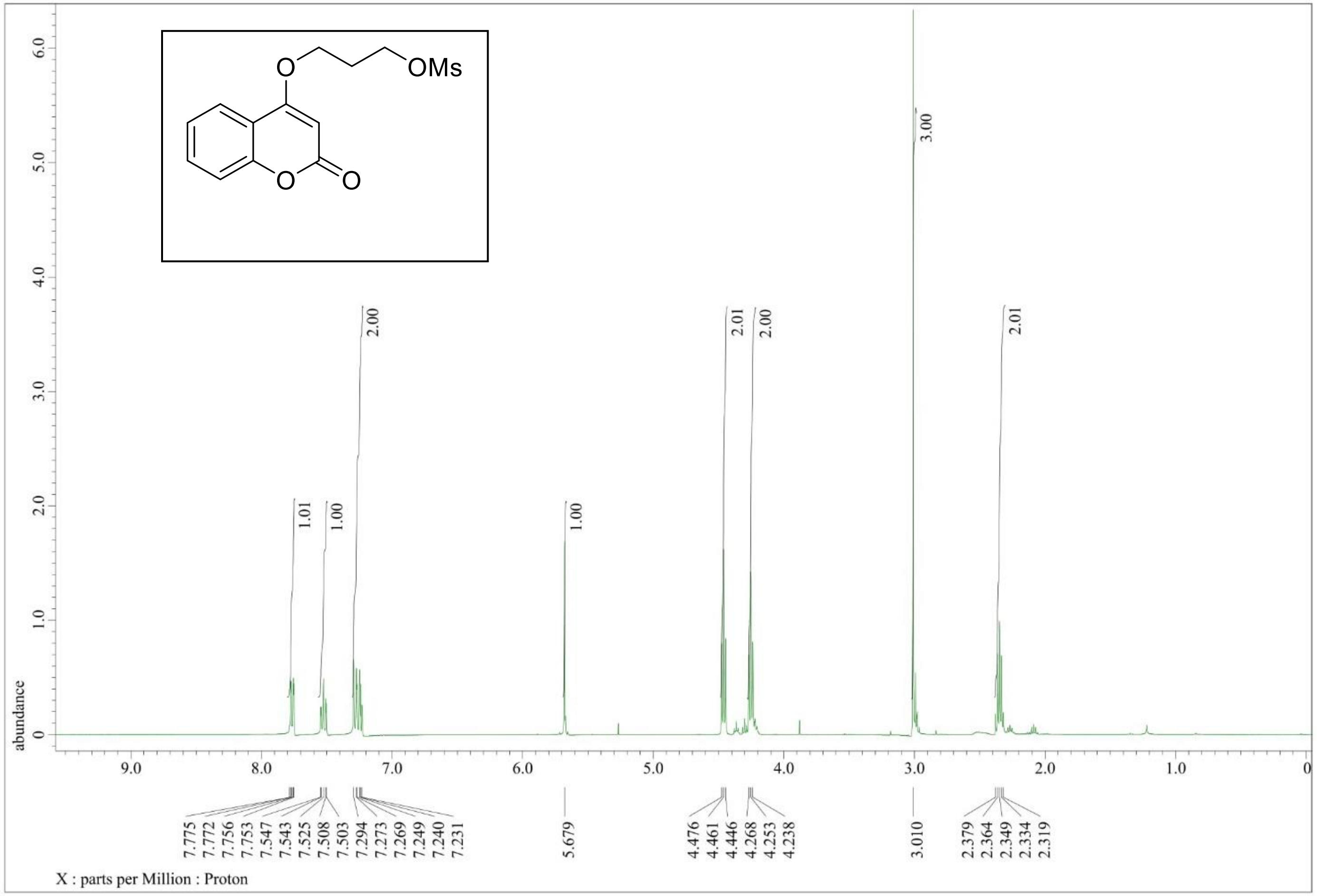

3-((2-oxo-2H-chromen-4-yl)oxy)propyl methanesulfonate (entry 7 in Table 2)

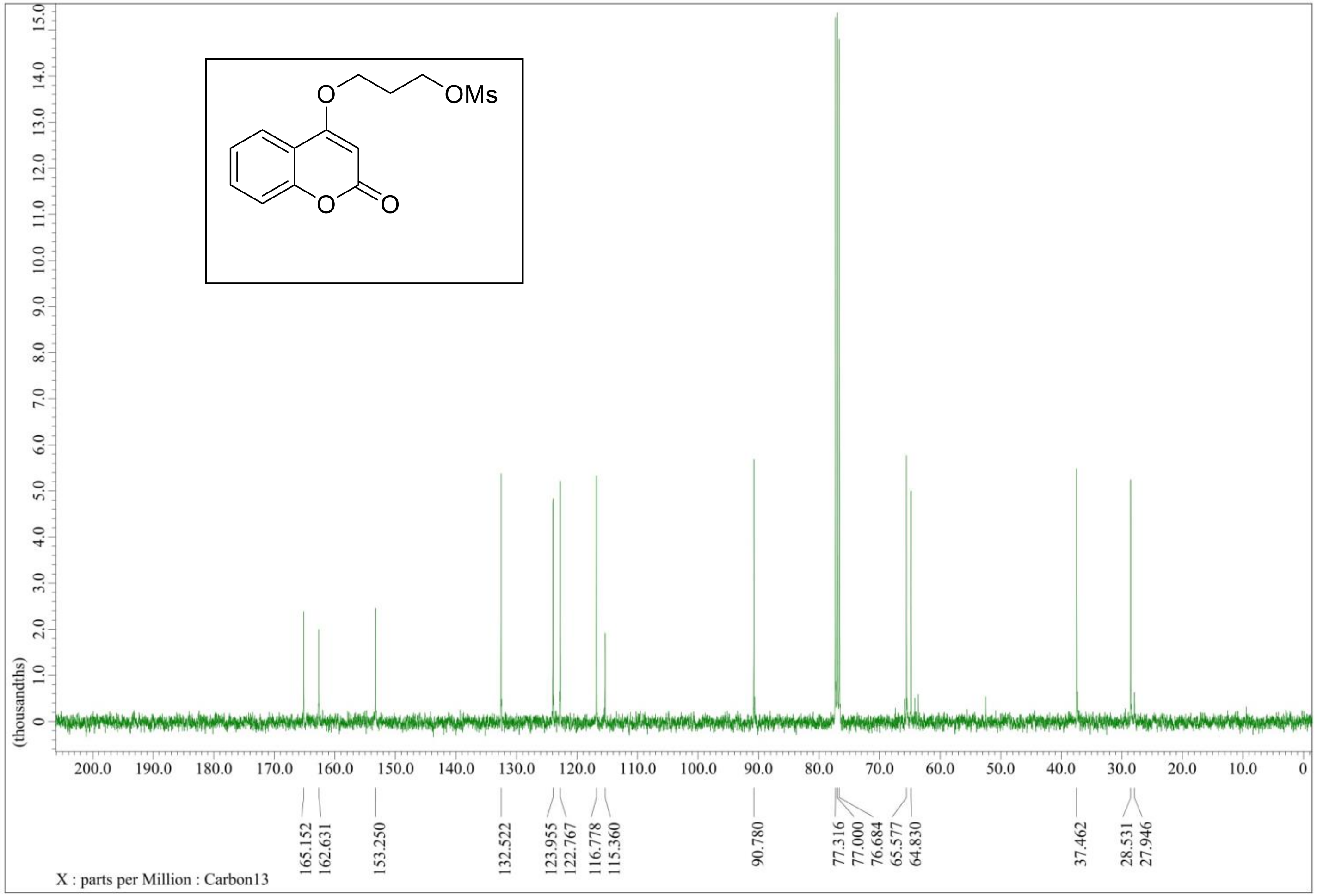

4-(3-bromopropoxy)-2H-chromen-2-one (entry 8 in Table 2)

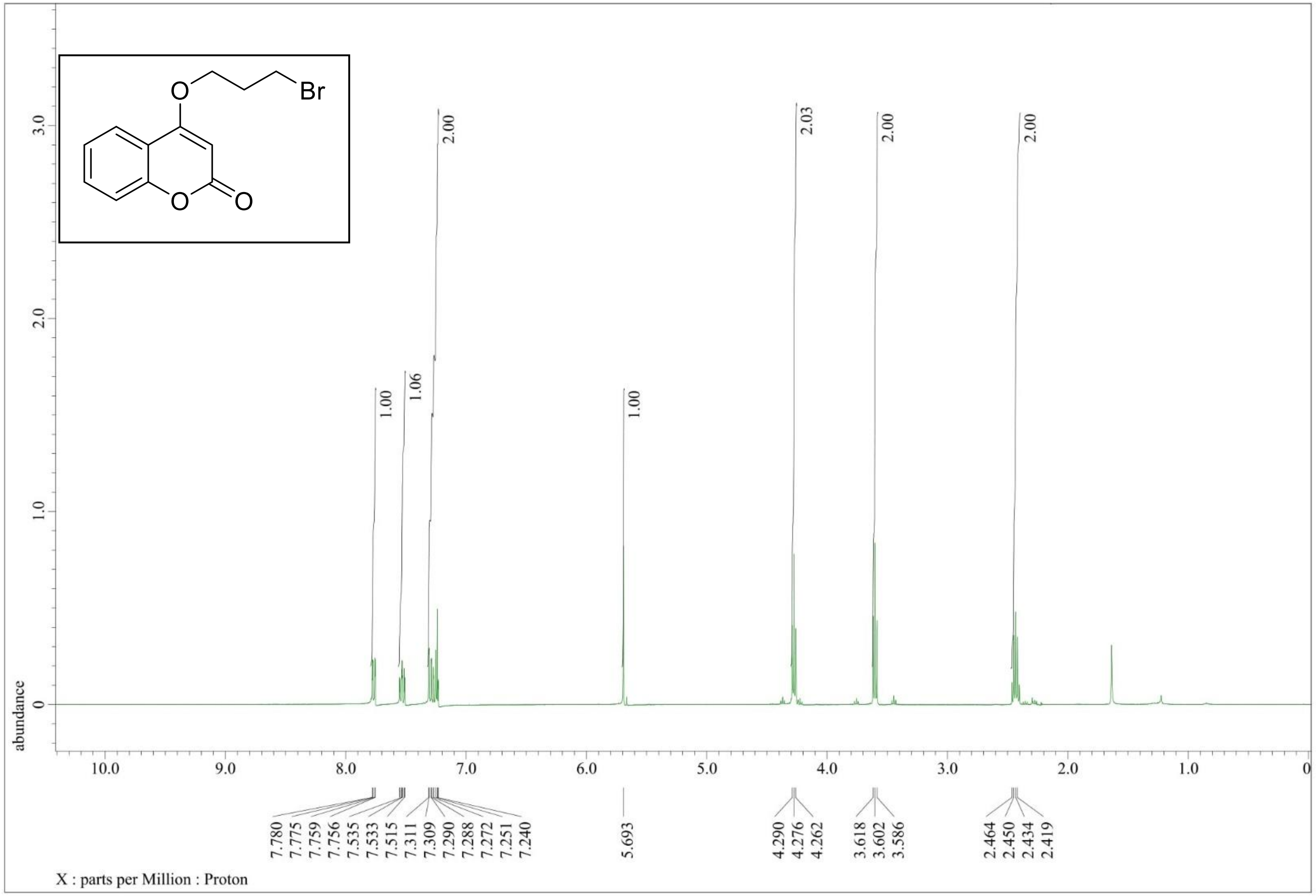

4-(3-bromopropoxy)-2H-chromen-2-one (entry 8 in Table 2)

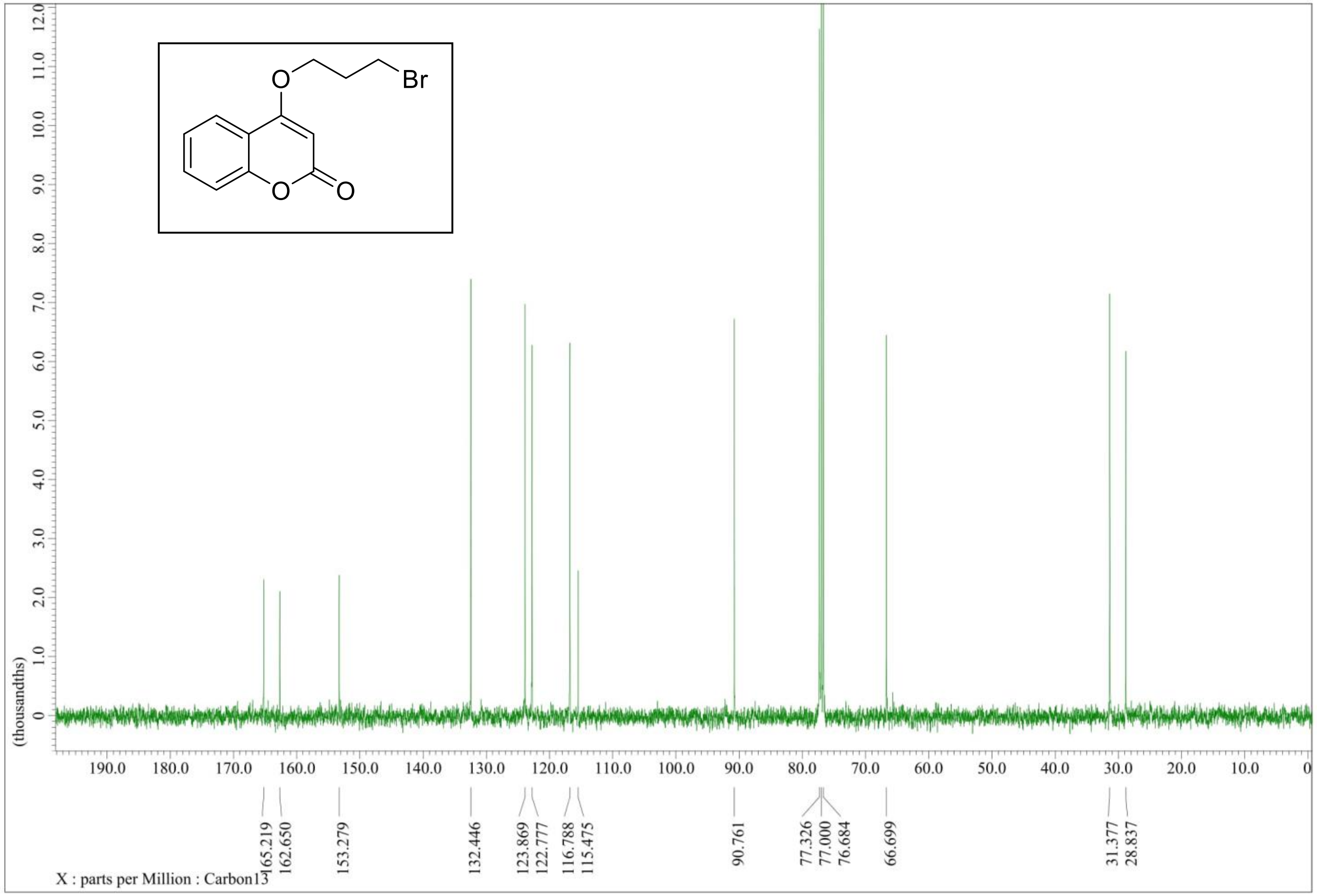

4-(3-iodopropoxy)-2H-chromen-2-one (entry 9 in Table 2)

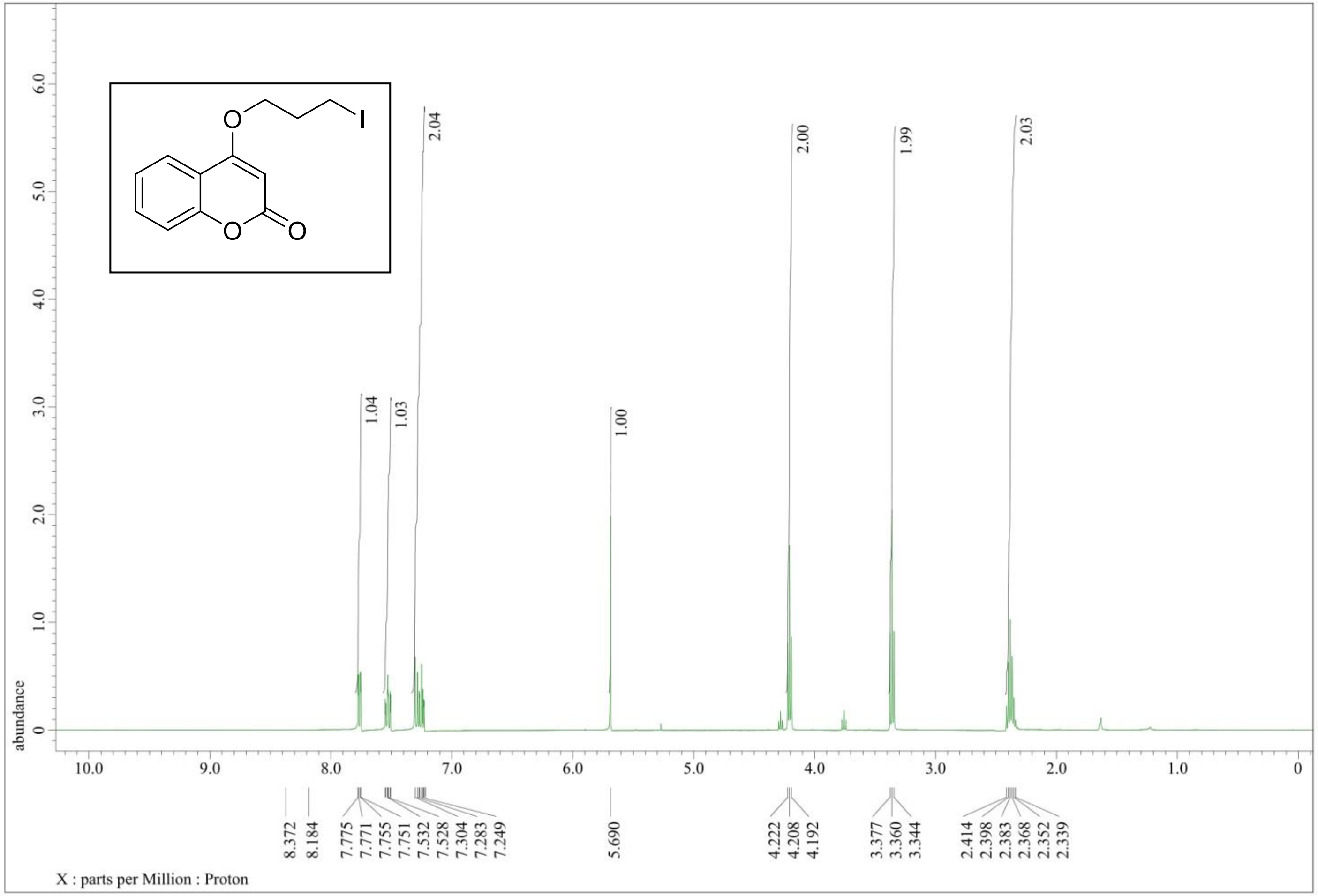

4-(3-iodopropoxy)-2H-chromen-2-one (entry 9 in Table 2)

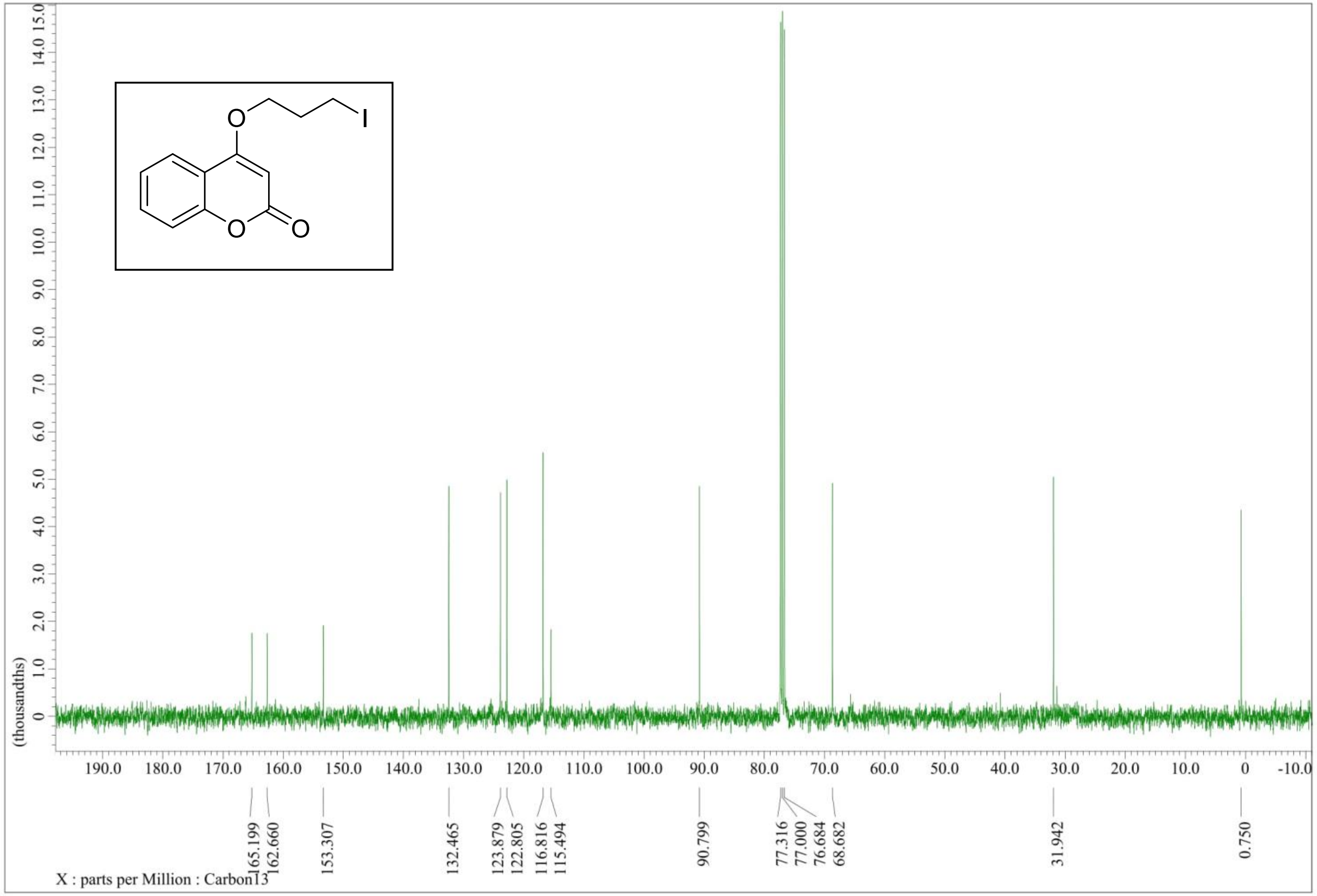

3-(4-nitrophenoxy)propyl methanesulfonate (entry 11 in Table 2)

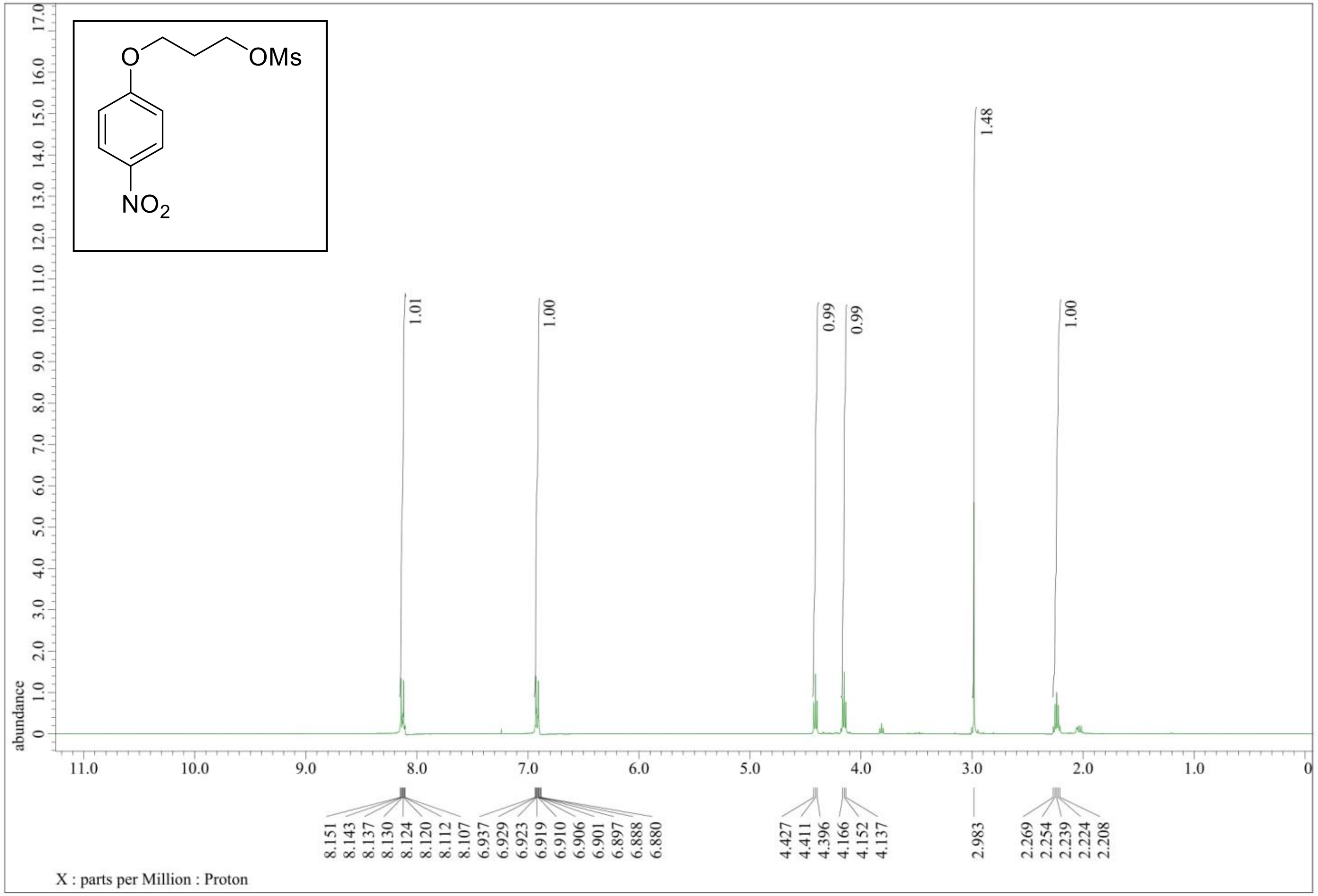

3-(4-nitrophenoxy)propyl methanesulfonate (entry 11 in Table 2)

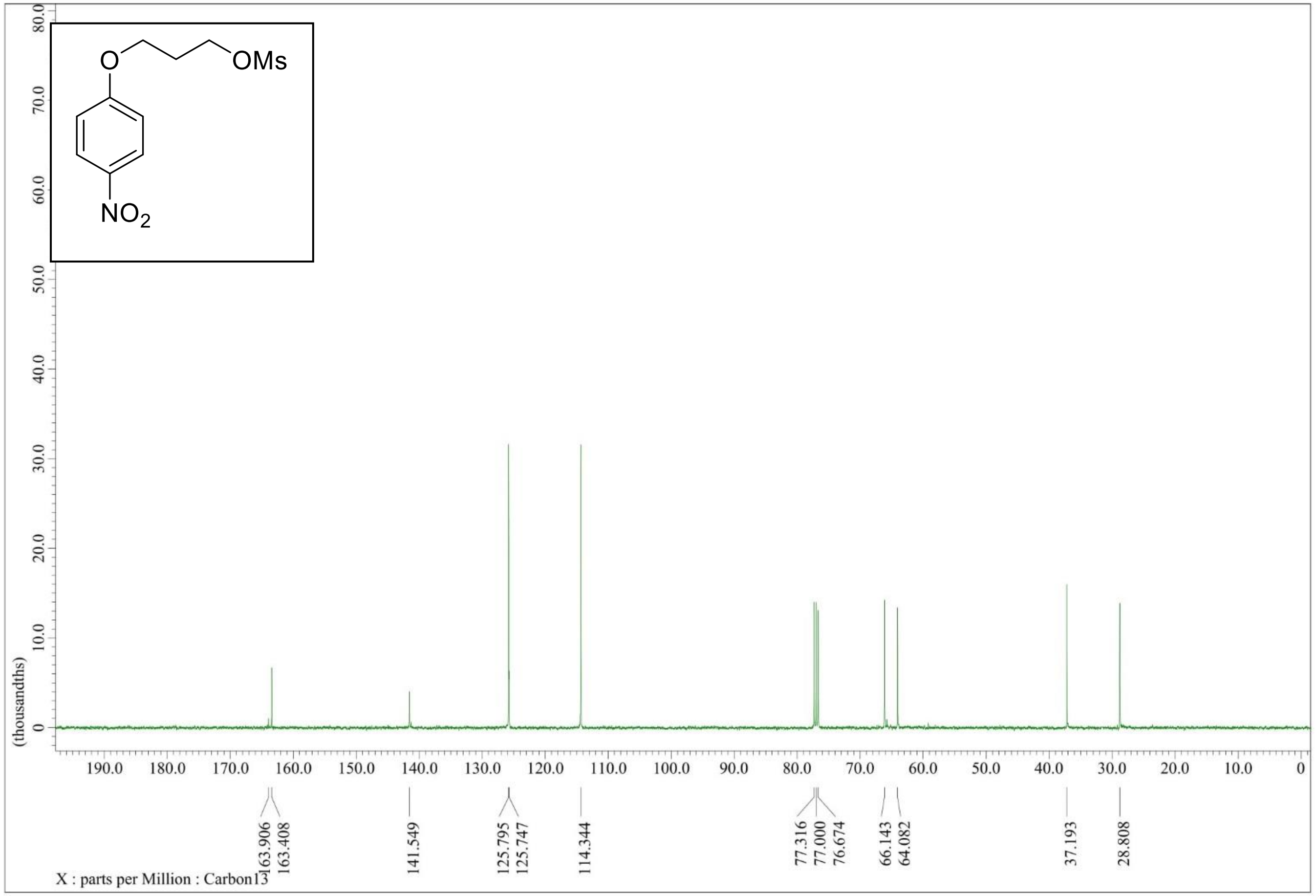

1-(3-Bromopropyl)-4-nitroimidazole (entry 12 in Table 2):

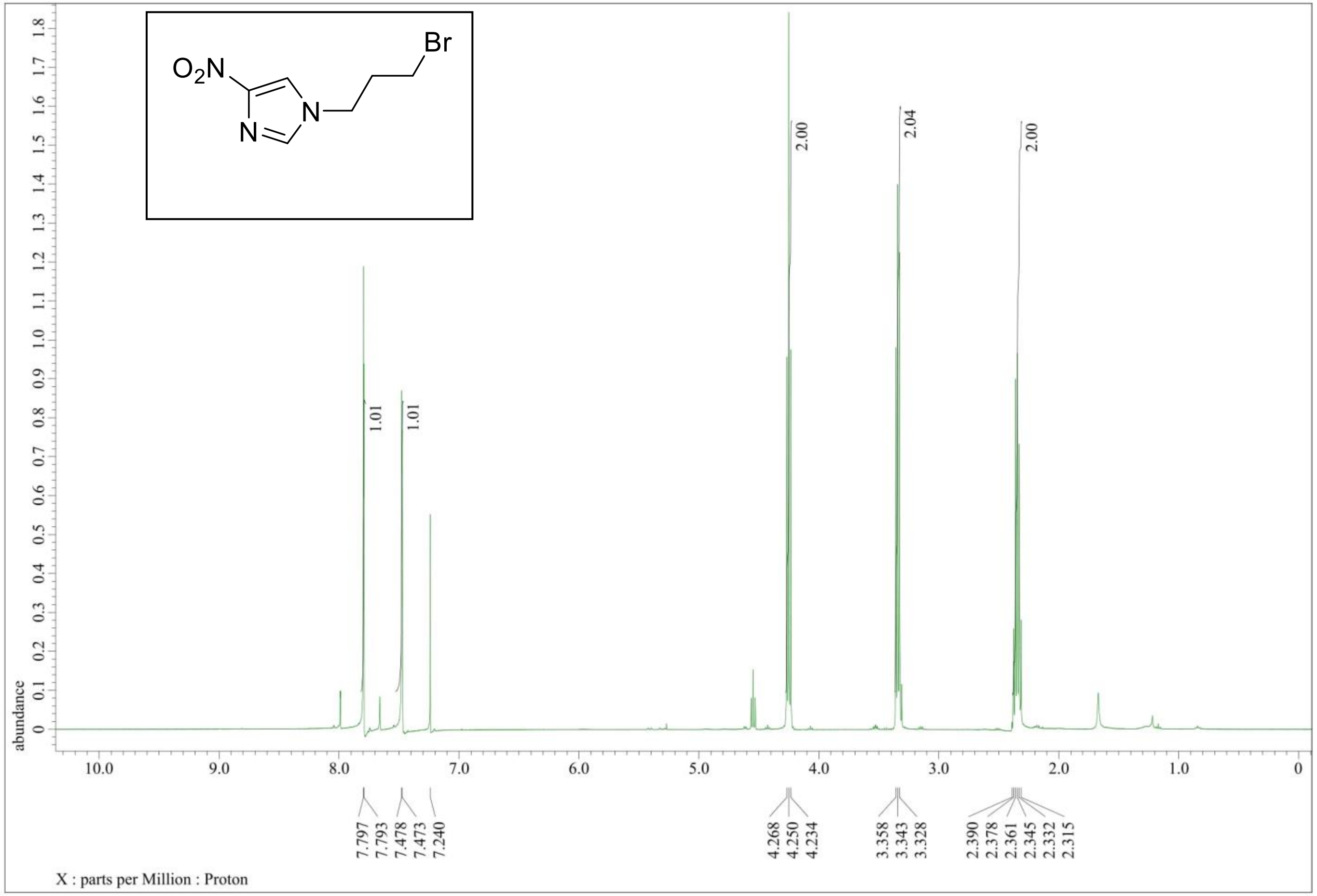

1-(3-Bromopropyl)-4-nitroimidazole (entry 12 in Table 2):

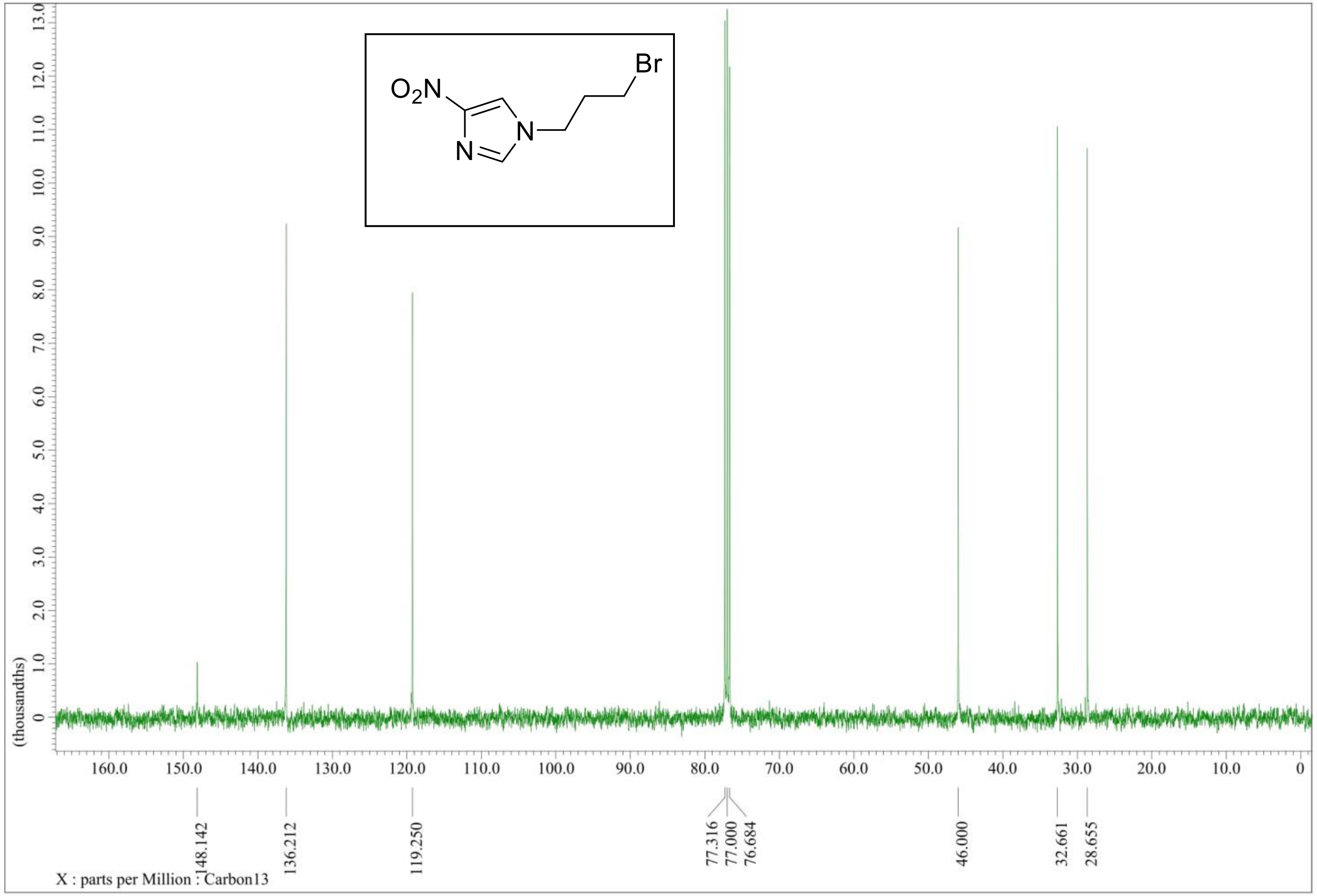

1,2:3,4-Di-*O*-isopropylidene-6- methanesulfonate -6-deoxy-α-D- galactopyranose (entry 13 in Table 2)

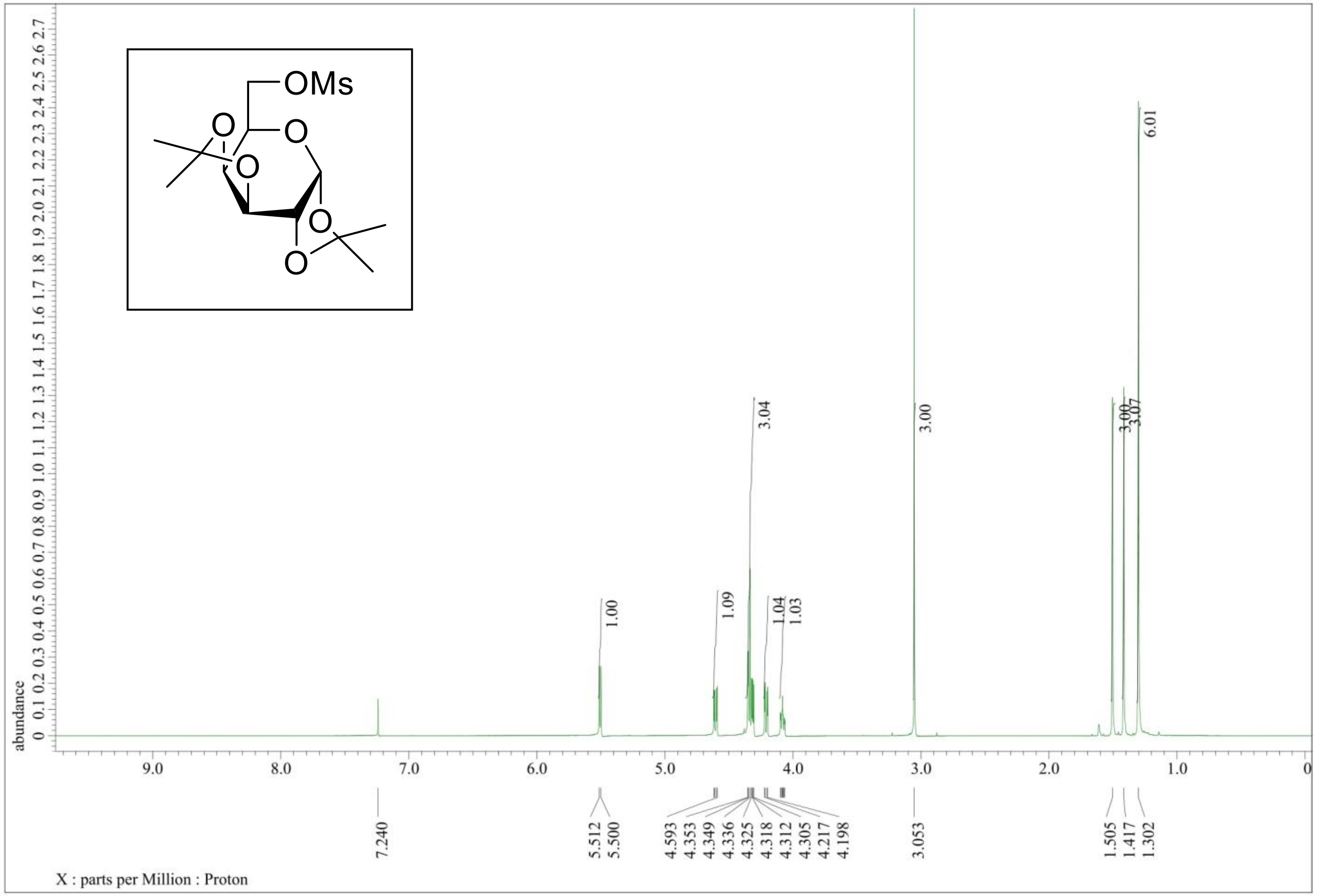

1,2:3,4-Di-*O*-isopropylidene-6- methanesulfonate -6-deoxy-α-D- galactopyranose (entry 13 in Table 2)

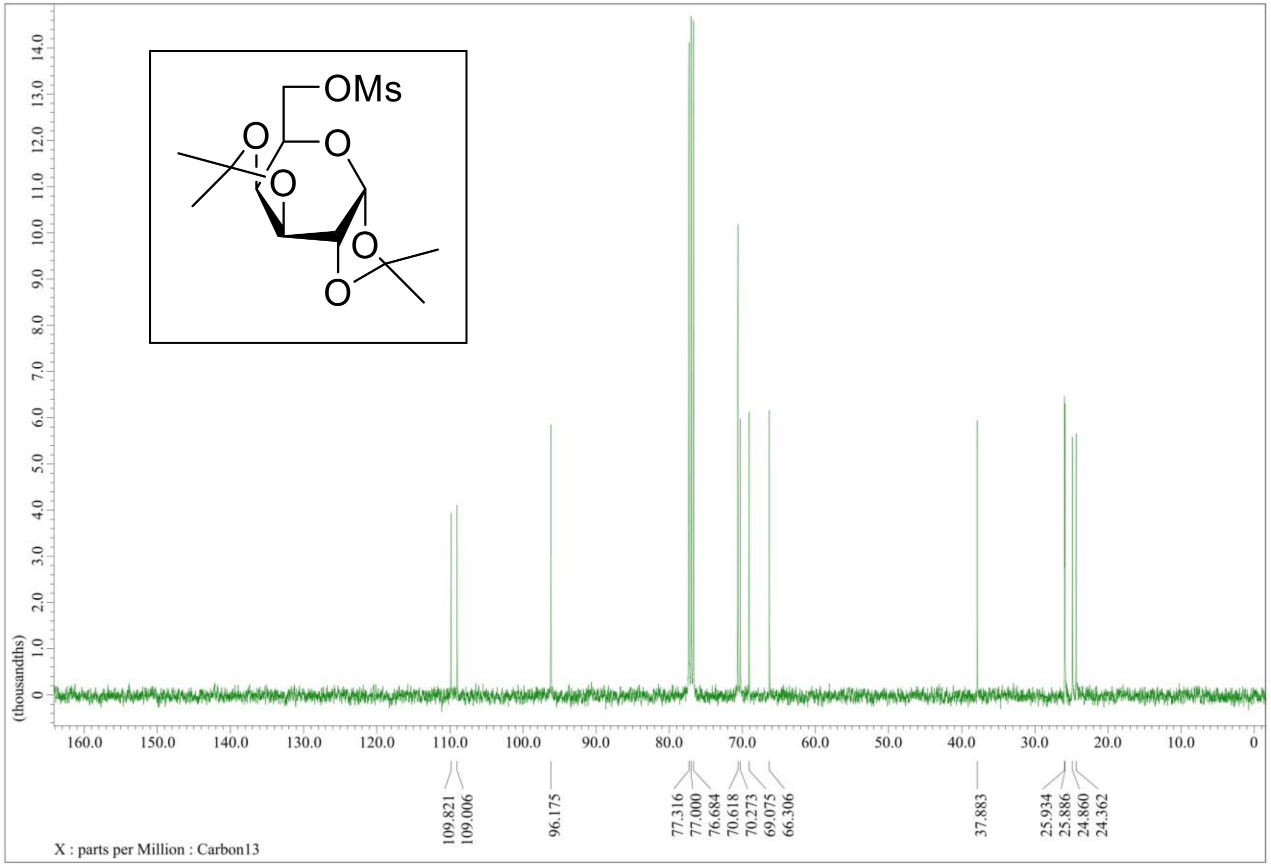

3-Chloro-picoline N-oxide (entry 14 in Table 2)

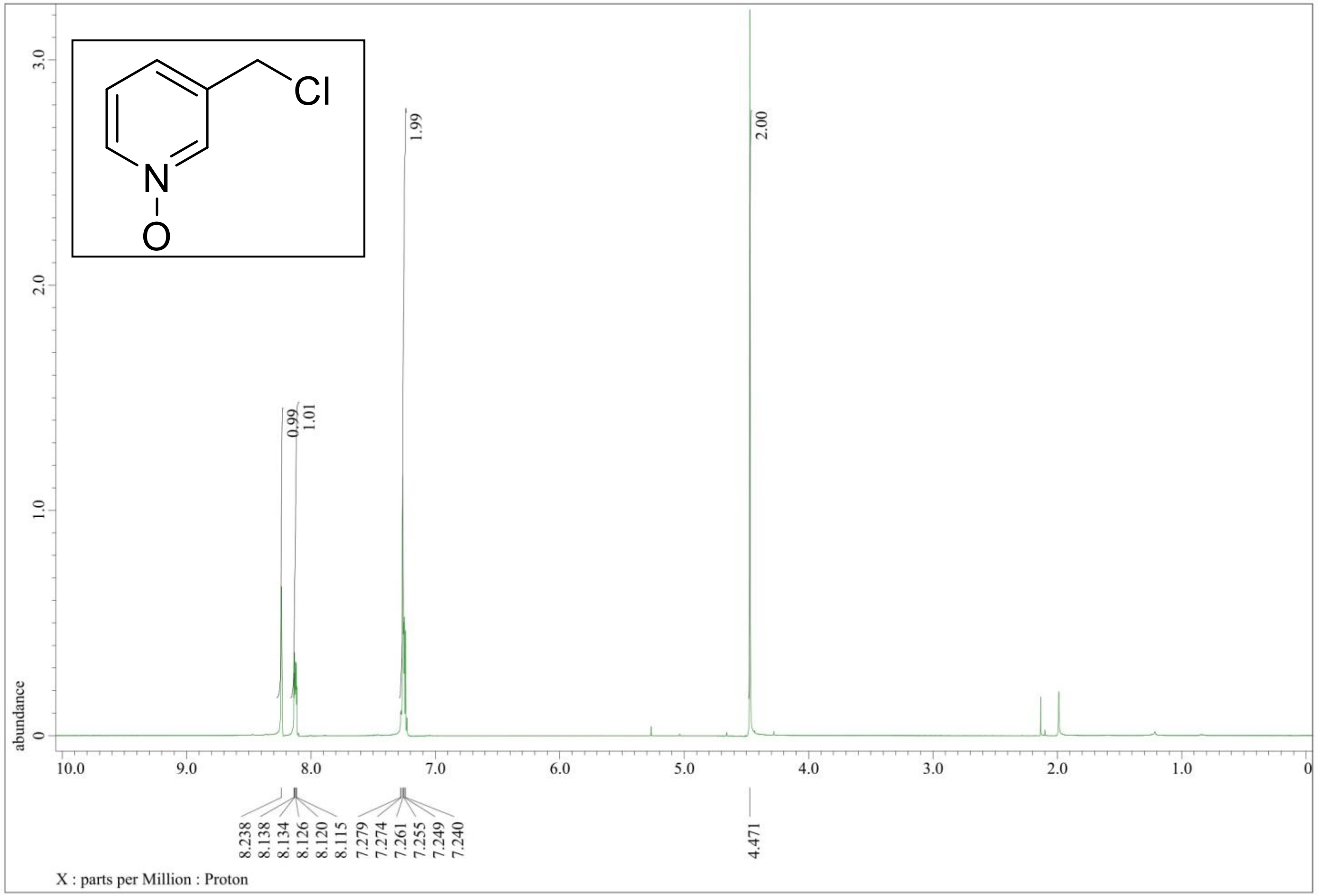

3-Chloro-picoline N-oxide (entry 14 in Table 2)

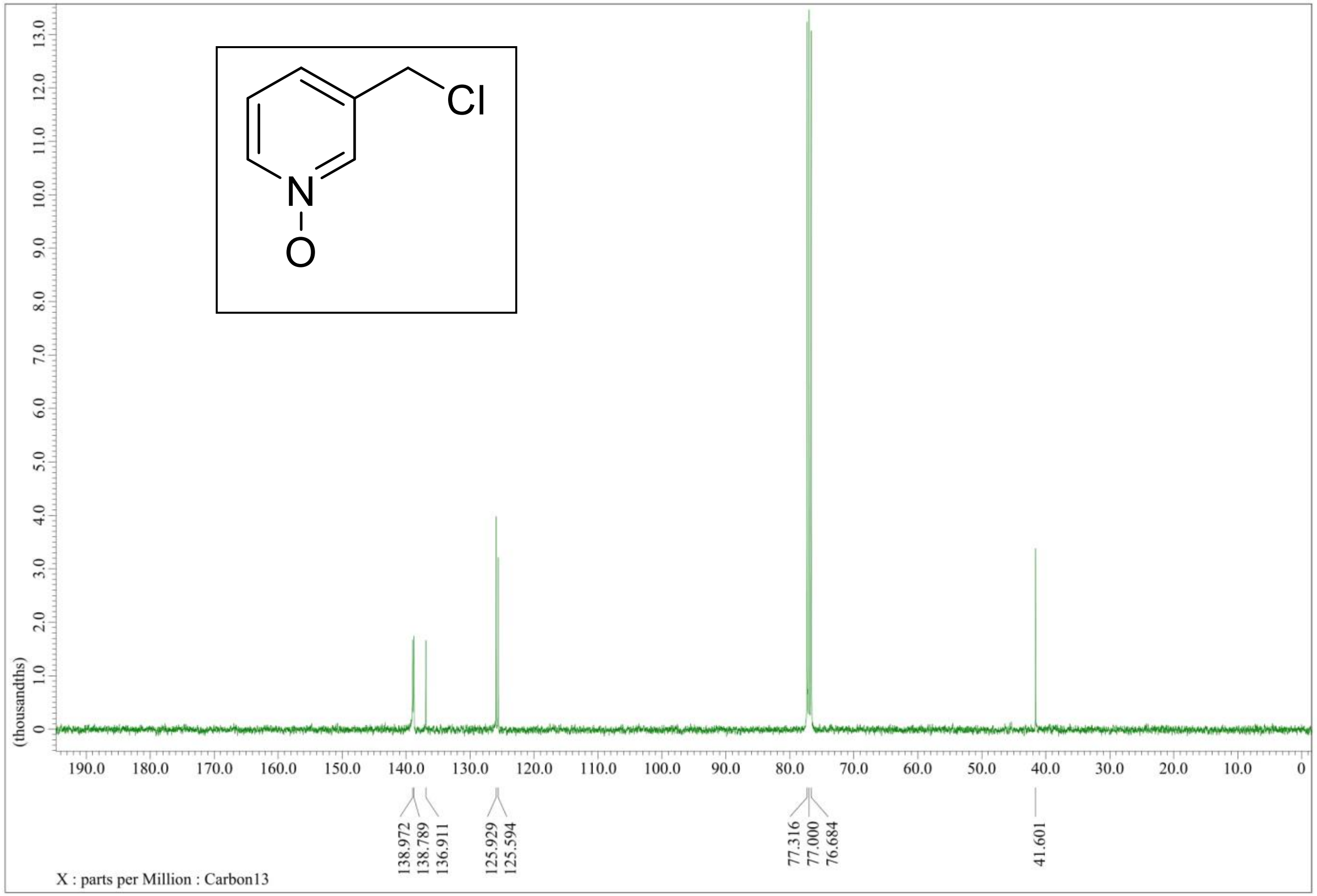

3-*O*-(3-Methanesulfonylpropyl)estrone (entry 16 in Table 2)

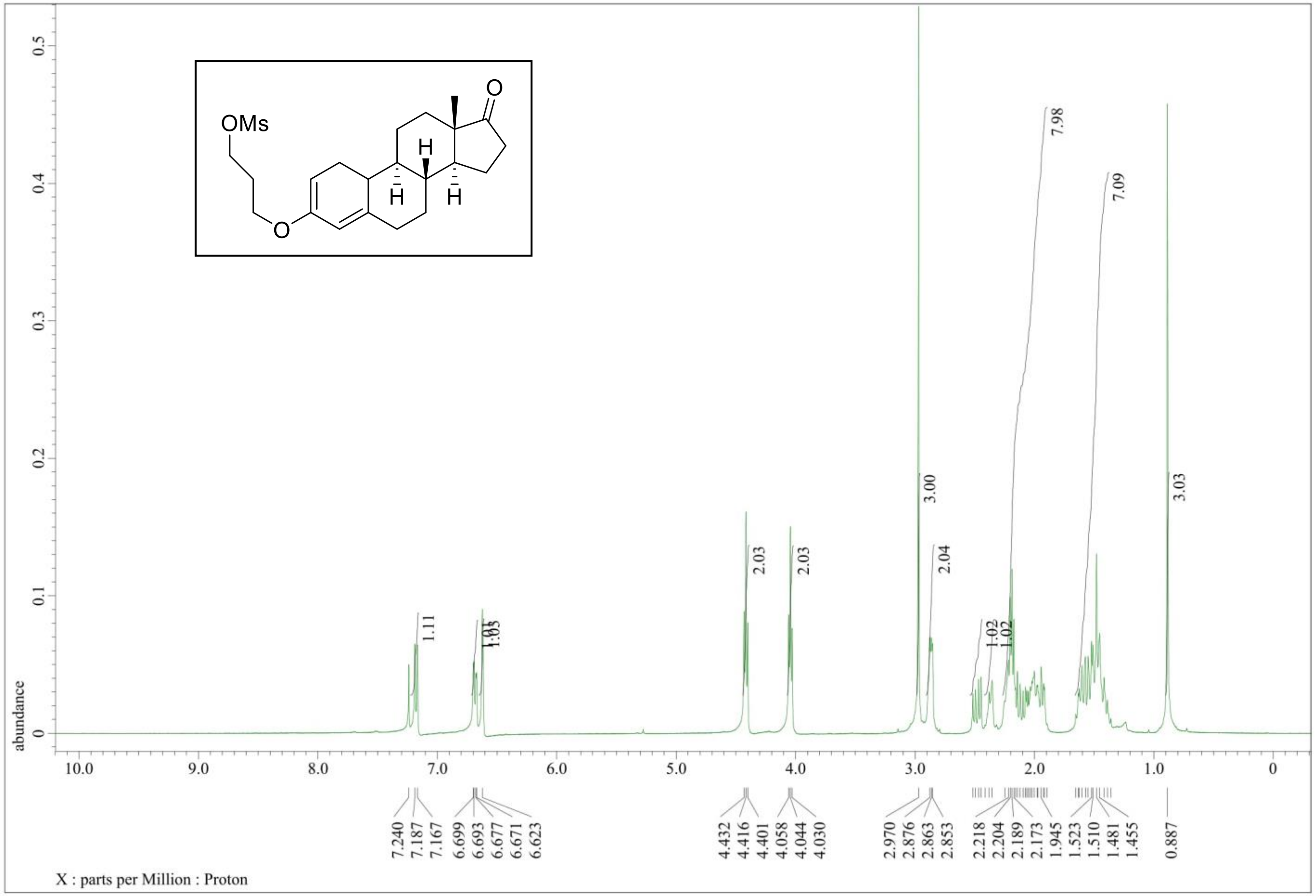

3-*O*-(3-Methanesulfonylpropyl)estrone (entry 16 in Table 2)

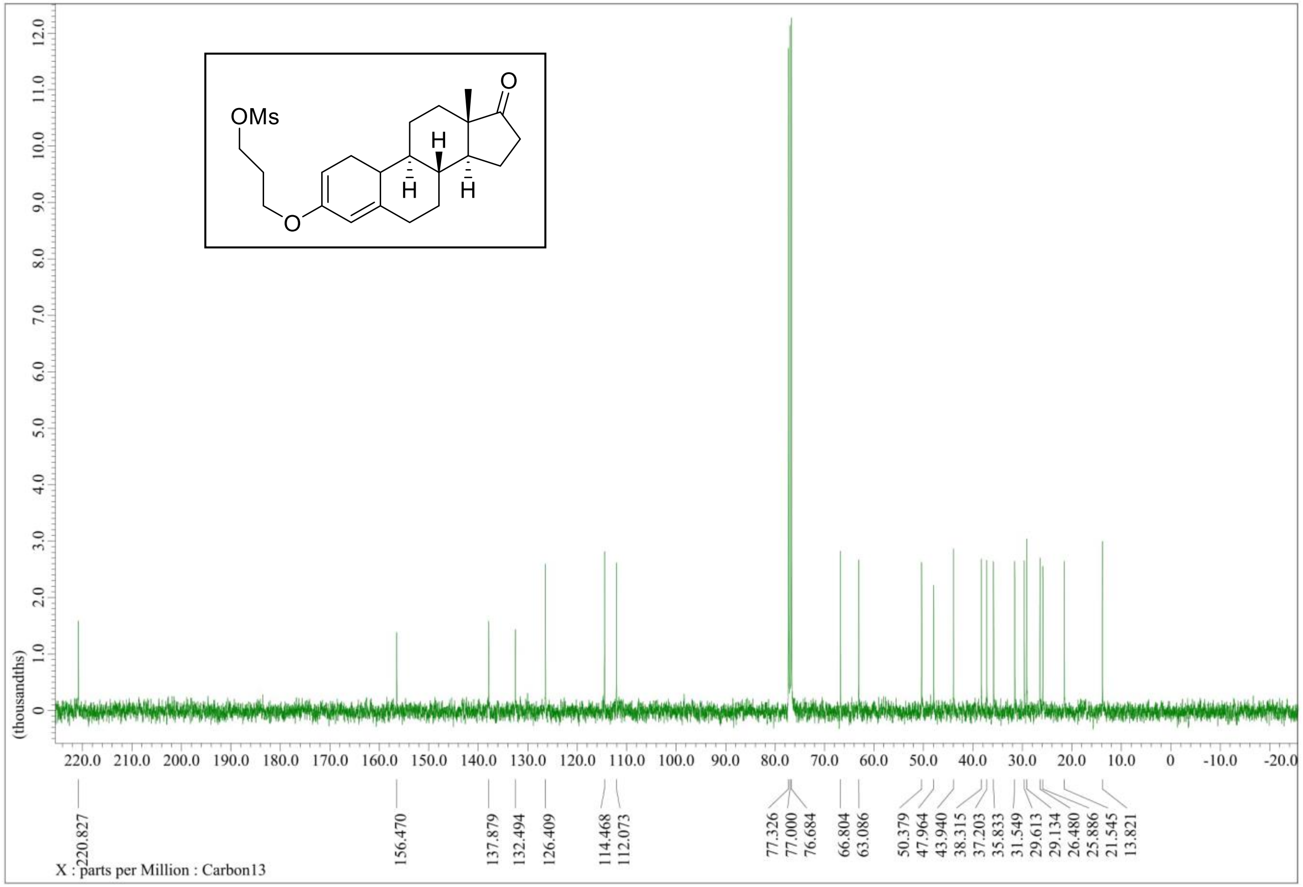

Supplement: Supplementary file 1 [file molecules-28-06747-s001.zip › molecules-2586742-supplementary.pdf]
